# Establishing the cell biology of apomictic reproduction in diploid *Boechera stricta* (Brassicaceae)

**DOI:** 10.1093/aob/mcy114

**Published:** 2018-07-06

**Authors:** Joanna Rojek, Małgorzata Kapusta, Małgorzata Kozieradzka-Kiszkurno, Daria Majcher, Marcin Górniak, Elwira Sliwinska, Timothy F Sharbel, Jerzy Bohdanowicz

**Affiliations:** 1 Department of Plant Cytology and Embryology, Faculty of Biology, University of Gdańsk, Poland; 2 Department of Molecular Evolution, Faculty of Biology, University of Gdańsk, Poland; 3 Laboratory of Molecular Biology and Cytometry, Department of Agricultural Biotechnology, UTP University of Technology and Life Sciences in Bydgoszcz, Poland; 4 Global Institute for Food Security, University of Saskatchewan, Saskatoon, Saskatchewan, Canada

**Keywords:** Apomixis, arabinogalactans, *Boechera*, *Boechera stricta*, callose, cell biology, flow cytometry, heterochrony, hybrid, microsatellites, meiosis, sexual reproduction

## Abstract

**Background and aims:**

In the Brassicaceae family, apomictic development is characteristic of the genus *Boechera.* Hybridization, polyploidy and environmental adaptation that arose during the evolution of *Boechera* may serve as (epi)genetic regulators of apomictic initiation in this genus. Here we focus on *Boechera stricta*, a predominantly diploid species that reproduces sexually. However, apomictic development in this species has been reported in several studies, indicating non-obligate sexuality.

**Methods:**

A progressive investigation of flower development was conducted using three accessions to assess the reproductive system of *B. stricta*. We employed molecular and cyto-embryological identification using histochemistry, transmission electron microscopy and Nomarski and epifluorescence microscopy.

**Key Results:**

Data from internal transcribed spacer (ITS) and chloroplast haplotype sequencing, in addition to microsatellite variation, confirmed the *B. stricta* genotype for all lines. Embryological data indicated irregularities in sexual reproduction manifested by heterochronic ovule development, longevity of meiocyte and dyad stages, diverse callose accumulation during meiocyte-to-gametophyte development, and the formation of triads and tetrads in several patterns. The arabinogalactan-related sugar epitope recognized by JIM13 immunolocalized to one or more megaspores. Furthermore, pollen sterility and a high frequency of seed abortion appeared to accompany reproduction of the accession ES512, along with the initiation of parthenogenesis. Data from flow cytometric screening revealed both sexual and apomictic seed formation.

**Conclusion:**

These results imply that *B. stricta* is a species with an underlying ability to initiate apomixis, at least with respect to the lines examined here. The existence of apomixis in an otherwise diploid sexual *B. stricta* may provide the genomic building blocks for establishing highly penetrant apomictic diploids and hybrid relatives. Our findings demonstrate that apomixis *per se* is a variable trait upon which natural selection could act.

## INTRODUCTION

### Current knowledge about apomixis in flowering plants

Apomixis is an asexual reproductive strategy in plants whereby seeds are produced via modified or absent meiosis and fertilization, to produce embryos that are genetically identical to the mother (Tucker and Koltunow, 2009). Hybridization and polyploidization are common characteristics of asexual (apomictic) plants and (parthenogenetic) animals, and there is evidence that these traits can act as inducers and/or stabilizers of asexuality relative to sexual ancestors (Neiman *et al.*, 2014; Bicknell and Catanach, 2015; Mau *et al.*, 2015 and references therein). Gametophytic and sporophytic apomixis is heritable, but these phenomena are generally expressed facultatively in individual plants, along with sex (Ozias-Akins and van Dijk, 2007). The coexistence of both apomixis and sexual reproduction within individual plants suggests that apomixis and sexuality are not mutually exclusive traits, but rather that apomixis is reversibly superimposed upon the sexual pathway (Hand and Koltunow, 2014; Hojsgaard *et al.*, 2014).

Reproduction in certain sexual plant species is reminiscent of the apomictic pathway, with occasional fertilization-independent development of the embryo, endosperm, maternal seed coat and fruit (e.g. Koltunow, 1993; Curtis and Grossniklaus, 2008; Brownfield and Köhler, 2011). Research on the initiation of apomixis in sexuals has been focused on the mechanisms responsible for the disruption or inhibition of meiosis in megaspore mother cells (MMCs), and the factors that stimulate unfertilized cells to undergo development (Ravi *et al.*, 2008; Eckardt, 2011; Crismani *et al.*, 2013). For example, mutation of the *POLYCOMB REPRESSIVE COMPLEX 2* (*PRC-2*) genes in *Arabidopsis* confers a degree of haploid embryo development (Guitton and Berger, 2005) and/or autonomous endosperm development (Ohad *et al*., 1996, 1999; Kiyosue *et al.*, 1999; Luo *et al.*, 1999; Vinkenoog and Scott, 2001). Autonomous endosperm can also be induced in wild *Arabidopsis* genotypes and other taxa within the Brassicaceae using chemical agents *in vitro* (Rojek *et al*., 2005, 2013, 2015; Kapusta *et al.*, 2007; Figueiredo *et al.*, 2015).

Considering various cytological traits of sexual sporogenesis, deviation from the meiotic pattern can be a hallmark of apomictic seed development. The accumulation of callose and arabinogalactans at appropriate sites and the timing of megasporogenesis underlie sexual reproductive development in *Arabidopsis* and likely in other angiosperms (Barcaccia and Albertini, 2013; Tucker and Koltunow, 2014 and references therein). The classical arabinogalactan protein 18 (AGP18) marks germ-line cell types during megasporogenesis in basal/early-divergent angiosperms (Lora *et al.*, 2017) and exerts active regulation over the selection and survival of megaspores in both basal angiosperms and *Arabidopsis*, suggesting that AGPs play an important role in the development of a single megaspore into a gametophyte (Demesa-Arévalo and Vielle-Calzada, 2013).

Callose deposition patterns may vary between species (Rodkiewicz, 1970), but in most cases callose is present in the mature MMC and then in the transverse walls that separate the megaspores during meiosis. After meiosis, callose persists in degenerating megaspores and in the part of the cell wall of the functional megaspore (FM) that is closest to the degenerating megaspores, while diminishing elsewhere, possibly due to the activity of β-1,3-glucanase enzymes, which target callose for degradation (Tucker *et al.*, 2001; Tucker and Koltunow 2009; Levy *et al.*, 2007). Mutants lacking glycosyl transferase 48 (GT48) glucan synthase-like (GSL) gene activity, which has been implicated in callose biosynthesis, exhibit defects in male fertility (Shi *et al.*, 2015) and disturbances in the ploidy of both male and female gametes via the induction of cell wall defects and induction of polyploidy (de Storme *et al.*, 2013; Tucker and Koltunow, 2014).

Whether the presence of callose or its deposition in a particular pattern around the megaspores influences their development remains unclear, but, interestingly, differences in callose accumulation are found between meiotic and apomeiotic megaspore formation pathways (e.g. Albertini and Barcaccia, 2007; Musiał *et al.*, 2015, and see references therein for review). The absence of callose deposition in walls of apomictic initial cells (AICs) seems a rule in diplosporous and aposporous ovules, and is inferred as a consequence of apomeiosis (Albertini and Barcaccia, 2007; Musiał *et al.*, 2015 and references therein). However, callose presence during diplosporous-type meiosis in two *Taraxacum* species (Musiał *et al.*, 2015) and *Chondrilla juncea* (Musiał and Kościńska-Pająk, 2017) and the callose-rich cell walls of MMCs in sexual and apomictic *Hieracium* subgenus *Pilosella* species (Tucker *et al.*, 2001) together suggest that temporary and local callose distribution fulfils a role during both sexual and apomeiotic megasporogenesis, and points to mechanisms that imprint functional cell (MMC/AIC or FM/AIC) identity (see also Okada *et al.*, 2013; Schmidt *et al.*, 2014).

### Sexuality and apomixis in *Boechera*

In the Brassicaceae, apomictic development is characteristic of the genus *Boechera* (formerly *Arabis*; Al-Shehbaz, 2003), occurring in North America, Greenland and the Russian Far East, with the highest number of species found in the western USA (Al-Shehbaz and Windham, 2010). The *Boechera* genus has a history of 2–5 million years and currently contains ±83 sexual diploid taxa, of which at least 64 have been involved in the hybrid genesis of hundreds of additional taxa in North America (Kiefer *et al*., 2009; Li *et al.*, 2017 and references therein). Many *Boechera* species are diploid and predominantly inbreeding, as indicated by genetic and molecular analyses (Schranz *et al.*, 2005; Song and Mitchell-Olds, 2007). Recent data indicate that nearly 50 % of the members of this genus are polyploid (mainly triploid) and characterized by apomictic reproduction (Koch, 2015). Furthermore, *Boechera* is the best-studied example of diploid apomixis in angiosperms (Dobeš *et al.*, 2006; Ozias-Akins and van Dijk, 2007). Apomictic diploid *Boechera* therefore provide a unique opportunity to examine evolutionary questions related to asexuality, independent of the effects of polyploidy, in relatively undisturbed habitats (Rushworth *et al.*, 2011).

**Fig. 1.**
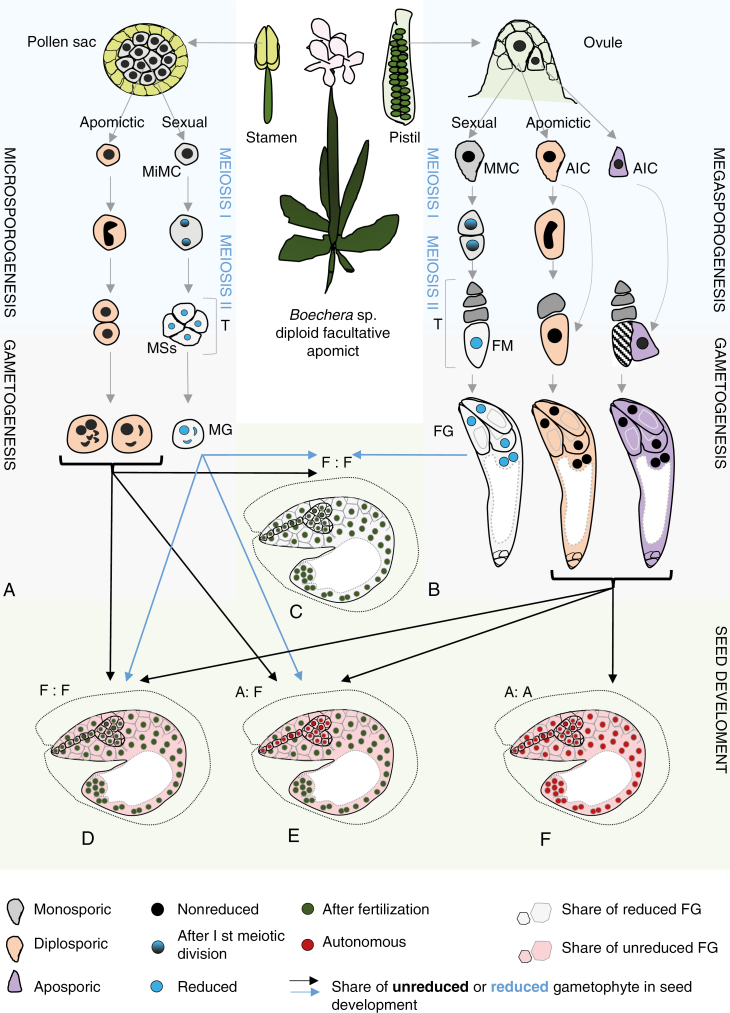
Summary of sexual and apomictic reproduction in *Boechera*. Diploid *Boechera* plants exhibit highly variable modes of seed formation, from obligate sexuality, through variable relative levels of sexual and apomictic seed formation in individual taxa, populations and plants, to obligate apomixis. (A, B) Germline development starts with the differentiation of sporophytic cells into spore mother cells [megaspore mother cell (MMC) in the ovule; microspore mother cell (MiMC) in the pollen sacs of the anther]. In the sexual pathway, MMC and MiMC undergo meiosis to give rise to a tetrad (T) of haploid spores. The four microspores (MSs) separate, grow and undergo two mitotic divisions to form trinuclear pollen [male gametophyte (MG)]. At the end of female sporogenesis three of the four spores degenerate, leaving one functional megaspore (FM), which undergoes mitotic divisions followed by nuclear migration and cellularization, eventually leading to the formation of a mature *Polygonum*-type female gametophyte (FG). In the apomictic pathway, different mechanisms can lead to the formation of unreduced gametes in *Boechera*. In *Taraxacum*-type diplospory, the apomictic initial cell (AIC) may initiate meiosis but restitution results in the formation of two unreduced cells, one of which degenerates; the second becomes an FM-like cell. *Antenaria* type diplospory completely omits meiosis and the AIC directly becomes an FM-like cell. By contrast, in apospory an FM-like cell is generated directly from a sporophytic nucellar cell in the ovule that is at a position different from that of the MMC. The unreduced AICs then develop into unreduced FGs. Apomeiosis on the male side is expressed in the interruption of the first and/or second meiotic division, which leads to formation of a heterogeneous (diploid, aneuploid) pollen population. (C–F) Seed development in facultative *Boechera* apomicts depends on sexual events since the meiotically derived FG and the central cell of the apomictic FG commonly require fertilization. Embryo and endosperm formation after fertilization [blue arrows; fertilized egg:fertilized central cell (F:F)] are characteristic of sexual seed production in *Boechera* (C). However, reduced female gametes may be fertilized by unreduced apomictic sperm cells (black arrow), increasing seed embryo and endosperm ploidy. Unreduced (via apomeiosis) female gametophytes develop mostly into pseudogamous seeds, i.e. they produce an embryo by parthenogenesis and endosperm after central cell fertilization (A:F in panel E). Endosperm ploidy depends on the variant of pollen (reduced or unreduced) that participates in fertilization. Further, an unreduced egg cell can be sporadically fertilized by reduced pollen or unreduced (one or two) sperm cells (e.g. from unreduced pollen grain containing four sperm cells and two vegetative cells; Voigt *et al.*, 2007), hence leading to paternal contribution to the embryo (D). In addition to pseudogamy, autonomous seeds may be produced (autonomous apomixis), which contain a parthenogenetically formed embryo and endosperm derived from an unfertilized central cell [autonomous embryo:autonomous endosperm (A:A) in panel F].

Diploid *Boechera* exhibit highly variable modes of seed formation (Fig. 1A–F), from obligate sexuality, through varying levels of sexual and facultative apomictic seed formation in individual taxa, populations and plants (and even within a single ovary/anther; Böcher, 1951; Naumova *et al.*, 2001; Aliyu *et al.*, 2010) to obligate apomixis, where seeds are exclusively derived from meiotically unreduced gametes and parthenogenetic embryo development (Naumova *et al.*, 2001; Aliyu *et al.*, 2010; Corral *et al.*, 2013; Mau *et al.*, 2013; Mateo de Arias, 2015). Triploid *Boechera* display a relatively uniform mode of obligate apomictic seed formation (Aliyu *et al.*, 2010; Voigt-Zielinski *et al.*, 2012). Tetraploid *Boechera* are rare and are characterized by both apomictic and sexual reproduction (Li *et al.*, 2017). Furthermore, male and female apomeiosis can occur together or separately, leading to the production of fertile meiotically reduced or unreduced embryo sacs and/or pollen (Mau *et al.*, 2013). While rare, pollination of sexual diploid plants with reduced pollen to transfer apomixis (epi-)genetic factors could contribute to the ubiquity of diploid apomictic hybrids across the genus (according to Lovell *et al.*, 2017 and also Aliyu *et al.*, 2010; Lovell *et al.*, 2013; Mau *et al.*, 2015).

Because *Boechera* apomicts coexist and cross with their sexual parents or relatives, within and between populations and species, taxonomic classifications are problematic and constantly updated. Interestingly, the first described apomict, formerly *B. holboellii* (*Arabis holboellii*; Böcher, 1951), which was previously considered to be a highly morphologically diverse and widespread species, has been split into five species, comprising *B. collinsii*, *B. pendulocarpa*, *B. retrofracta* and *B. polyantha*, with the remaining *B. holboellii* found only in Greenland (Rushworth, 2015). Another example, *B. divaricarpa*, which was previously considered to be the hybrid between *B. stricta* and *B. holboellii*, was in fact a ‘trash can’ of hybrids between *B. stricta* and countless other species, and is currently ascribed to hybrids between *B. stricta* and *B. sparsiflora*. Hybrids between *B. stricta* and *B. retrofracta* await updated nomenclature, and currently recognized and/or reclassified species are described in a number of papers (Windham and Al-Shehbaz 2006, 2007*a*, *b*; Al-Shehbaz and Windham 2010; Alexander *et al*., 2015; Rushworth 2015; Windham *et al.*, 2015).

### The origin of apomixis in *Boechera*

The genesis of apomixis in *Boechera* is highly associated with intra- and interspecific crosses (Carman, 1997; Koch *et al.*, 2003; Dobeš *et al.*, 2004*a*, *b*, 2007; Schranz *et al.*, 2005; Beck *et al*., 2012; Lovell *et al*., 2013, 2017; De Storme and Geelen, 2013; Mau *et al.*, 2015). Of the 485 taxa included in the *Boechera* microsatellite database (Li *et al.*, 2017), 400 are confirmed or inferred apomictic hybrids between divergent sexual diploid taxa.

Analyses of large numbers of single seeds (Aliyu *et al.*, 2010) support the multilocus control of apomixis, as suggested for other species (van Dijk, 2003; Ozias-Akins and van Dijk, 2007; Záveský *et al.*, 2007), and provide a framework for selecting genotypes for comparative omics analyses of different apomixis components (Sharbel *et al*., 2009, 2010).

The expression of two alleles, *APOLLO* (apomixis-linked locus) (Corral *et al.*, 2013) and *UPGRADE2* (unreduced pollen grain development) (Mau *et al.*, 2013), is highly correlated with apomeiotic egg and pollen formation in *Boechera*, and the biogeographical distribution of *APOLLO* demonstrates that its repeated introduction into novel sexual genetic backgrounds is associated with multiple independent origins of the apomixis phenotype (Mau *et al.*, 2015). Direct evidence for these alleles’ position on the heterochromatic *Het* chromosomes of *Boechera* is still lacking (according to Koch, 2015). A study comparing transcriptomes of microdissected MMCs from sexual *Arabidopsis* and apomictic *Boechera gunnisoniana* found multiple differences in pathways related to transcriptional regulation, cell cycle control, epigenetic regulation and hormone production (Schmidt *et al.*, 2014). Drought and heat stress have also been implicated in the switch between apomeiosis and meiosis (Project no. UTA01127 2013–2018, HATCH; https://reeis.usda.gov/web/crisprojectpages/1000552-cytological-and-molecular-characterizations-of-reproduction-in-sexual-and-apomictic-boechera-brassicaceae.html; Mateo de Arias, 2015), and implies that apomixis in *Boechera* is an epigenetically determined and potentially conserved polyphenism of sex.

Further information about the origin of apomictic *Boechera* has come from cytogenetic analyses. Mandáková and co-workers (2015; but also Sharbel *et al.*, 2004, 2005; Kantama *et al.*, 2007) showed that the largely heterochromatic *Het* chromosome is derived from *Boechera* chromosome 1 (BS1). Rearrangements of this *Het* chromosome, including centric fission and pericentric inversion, lead to the formation new *Het′* and *Del* chromosomes, explaining the cytogenetic characteristics of apomictic 2*n* = 15 species. The *Het′* and *Del* chromosomes are potential markers for the apomixis trait, and are hypothesized as possible elements that enable the transmission of apomictic factors into new sexual genetic backgrounds (Sharbel *et al*., 2004, 2005). As expected, apomictic *Boechera* lineages accumulate mutations at otherwise conserved genome sites more often than sexuals (i.e. Muller’s ratchet), implying that such mutations may be harmful to asexually reproducing populations (Lovell *et al.*, 2017). Taking the above findings together, the precise genetic basis of apomixis in *Boechera* remains unknown, but evidence implies that apomixis is heritable and caused by a number of processes (according to Rushworth, 2015).

### Cell biology of apomixis in *Boechera*

Apomictic *Boechera* species are characterized by gametophytic apomixis, whereby a female gametophyte (FG) (i.e. embryo sac; Reiser and Fisher, 1993) is formed from a diploid cell in the ovule, bypassing meiosis (i.e. apomeiosis; Hand and Koltunow 2014). *Taraxacum*-type diplospory, *Antennaria*-type diplospory and apospory have all been described in *Boechera* based on limited cytological analyses of female sporogenesis (Böcher, 1951; Naumova *et al.*, 2001; Taskin *et al*., 2009; Sharbel *et al.*, 2010; Schmidt *et al.*, 2014; Mateo de Arias, 2015; Windham *et al.*, 2015; Shah *et al.*, 2016; Osadtchiy *et al.*, 2017).

In the most frequently occurring *Taraxacum* type, an AIC at the position of the MMC undergoes meiosis I without completing the reduction phase, followed by normal meiosis II, leading to a dyad of unreduced megaspore-like cells, each of which exhibits the same ploidy as the mother plant. The micropylar megaspore-like cell of the diplosporous dyad degenerates, while the chalazal megaspore-like cell increases in size, undergoes vacuolization and three subsequent karyokineses, and finishes in a *Polygonum*-type unreduced embryo sac (Fig. 1B; Böcher, 1951; Naumova *et al.*, 2001; Mateo de Arias, 2015; Osadtchiy *et al.*, 2017). *Antennaria*-type diplospory, where meiosis is completely eliminated and the AIC directly forms an unreduced embryo sac (Koltunow, 1993; Crane, 2001), was infrequently observed only in *B. retrofracta* × *stricta* (Project no. UTA01127 2013-2018, HATCH; https://reeis.usda.gov/web/crisprojectpages/1000552-cytological-and-molecular-characterizations-of-reproduction-in-sexual-and-apomictic-boechera-brassicaceae.html).

There is evidence of apospory in *Boechera*: a type of gametophytic apomixis that involves the formation of an unreduced FG directly from nucellar or integument cells (Fig. 1B; see also Hand and Koltunow, 2014; Crane, 2001). Carman and co-workers (Project no. UTA01127 2013-2018, HATCH; https://reeis.usda.gov/web/crisprojectpages/1000552-cytological-and-molecular-characterizations-of-reproduction-in-sexual-and-apomictic-boechera-brassicaceae.html; Mateo de Arias, 2015) discovered that apospory occurs frequently in *B. crandallii*, *B. gracilipes*, *B. laevigata*, *B. lignifera*, *B. lincolnensis* hybrids, *B. microphylla*, *B. pulchra*, *B. retrofracta* hybrids and *B. thompsonii* hybrids. Furthermore, mixed apospory and diplospory has also been described in some accessions (Carman, 2007; Shah *et al.*, 2016; Mateo de Arias, 2015 and references therein), implying that apospory may be more common in *Boechera.*

As observed in many asexual taxa, microsporogenesis is irregular in apomictic individuals (Fig. 1A), and normal reduced, non-reduced and aneuploid pollen can be found within and between different genotypes (Böcher, 1951; Dobeš *et al.*, 2006; Sharbel *et al.*, 2005; Voigt *et al.*, 2007; Voigt, 2009; Mau *et al.*, 2013). The meiotic chromosome behaviour of apomicts producing unreduced pollen is primarily asynaptic (asyndetic according to Böcher, 1951) as the first meiotic division results in restitution without crossover. Univalents remain together during meiosis I and disjoin in meiosis II, when sister chromatids are equationally separated to opposite poles, leading to two balanced diploid chromosome sets in dyads (Kantama *et al.*, 2007; Mau *et al.*, 2013). As this mechanism is not fully penetrant in apomictic accessions, other events, such as unequal sister chromatid segregation during meiosis II, but also normal meiosis, can lead to the formation of heterogeneous pollen populations.

Although embryo development is almost exclusively parthenogenetic, apomictic seed production in *Boechera* is dependent on sexual events since the central cell commonly requires fertilization for proper endosperm formation (pseudogamy) (Fig. 1C–F). Pseudogamy in diploid apomictic *Boechera* is characterized mainly by a 2C (autonomous embryo):6C (pseudogamous endosperm) genome ratio in seeds, indicating a 100 % maternally derived embryo and a hexaploid endosperm produced via fertilization with an unreduced pollen cell. Thus, pseudogamously derived endosperm in diploid [4 maternal(m):2 paternal(p) genomes] and also triploid (6m:3p) apomicts support the endosperm balance hypothesis, where the meiotically unreduced ovule requires balanced (2 maternal:1 paternal) endosperm (e.g. Jullien and Berger, 2010; Aliyu *et al.*, 2010; Hand and Koltunow, 2014 and references therein). However, pseudogamous endosperm ploidies in obligate and facultative apomicts can range from 5C [i.e. fertilization of an unreduced (binuclear, 4C) central cell by reduced pollen with a haploid (C) male gamete] to 10C [i.e. fertilization of unreduced binuclear central cell by hexaploid (6C) pollen] (Aliyu *et al.*, 2010; Voigt-Zielinski *et al.*, 2012). More rarely, some apomictic *Boechera* produce autonomous seeds (autonomous apomixis) that containing parthenogenetically formed embryo and endosperm derived from an unfertilized central cell (Naumova *et al.*, 2001; Voigt, 2009; Aliyu *et al.*, 2010), and an unreduced (2C) egg cell can be sporadically fertilized by unreduced (2C) or reduced (C) pollen (Fig. 1D; for details see Voigt-Zielinski *et al.*, 2012).

The basic chromosome number of *Boechera*, *x* = 7 (Koch *et al.*, 1999; Koch, 2015 and references therein), has evolved from an ancestral karyotype (*n* = 8) of Brassicaceae lineage I. Comparative chromosome painting allowed Mandáková *et al.* (2015) to characterize an aberrant chromosome *Het* and its two derivatives (*Het′* and *Del*; previously defined as B chromosomes; Camacho *et al.*, 2000; Sharbel *et al.*, 2004, 2005; Kantama *et al.*, 2007) in apomictic diploid (2*n* = 14), triploid (2*n* = 21) and aneuploid (2*n* = 15, 2*n* = 22) apomictic *Boechera*, showing that new chromosomes can be formed by a centric fission and can be fixed in populations through apomixis (for more details see Mandáková *et al.*, 2015).

Despite rich cytogenetic data, cytoembryological analysis (mainly based on pollen microsporogenesis) is still lacking for many taxa/accessions, with scant knowledge with respect to the female reproductive pathway. The *Boechera* Microsatellite Website (http://sites.biology.duke.edu/windhamlab/) has the most cytogenetic data, but only for 5 % of all accessions in the database (4428 specimens encompassing >95 % of all named taxa) including both published (summarized in Dobeš *et al.*, 2006) and unpublished chromosome studies (according to Li *et al.*, 2017). Inferring the reproductive mode from the remaining accessions requires additional observations.

### *Boechera stricta* and apomixis

*Boechera stricta* (Graham) Al-Shehbaz (previously *Arabis drummondii*) has been shown to be predominantly diploid (2*n* = 2*x* = 14) and sexual, and can easily form hybrids with other *Boechera* species that are facultative and highly variable apomicts with respect to ploidy, morphology and genetic polymorphisms (Windham *et al.*, 2015). Most of the currently named *B. stricta* accessions have been (re-)established based on microsatellite identification (*Boechera* Microsatellite Website; Li *et al.*, 2017), with the SAD12 (ES06, Schranz *et al.*, 2007; ‘East’ subspecies, Lee and Mitchell-Olds, 2011, 2013) and LTM (‘West’ subspecies, Lee and Mitchell-Olds, 2011, 2013) reference accessions that are commonly used for the investigations of hybridization, speciation and apomixis. These two accessions show significant ecological differentiation across local environmental gradients involving multiple ecologically important traits, including flowering differences that are expected to increase reproductive isolation between them (Lee *et al.*, 2017).

Some populations or single individuals of *B. stricta* have been described as showing variable levels of apomixis (Aliyu *et al.*, 2010; Mau *et al.*, 2015). Reproduction was drastically altered in a few triploid *B. stricta* accessions in which apomictic development is obligate and autonomous formation of endosperm is possible (Aliyu *et al.*, 2010; Mau *et al.*, 2015), while *B. stricta* hybrids (e.g. *B. stricta* × *B. retrofracta* and *B. stricta* × *Boechera spatifolia*) have ~93–100 % frequencies of apomixis (Mau *et al.*, 2015).

This article presents the results of the molecular identification and extensive cyto-embryological studies involving three diploid *B. stricta* accessions: ‘East’ ES512 and ES655 (Schranz *et al*., 2005, 2007; Aliyu *et al.*, 2010; Mau *et al.*, 2015) and ‘West’ LTM (Lee and Mitchell-Olds, 2011, 2013). We performed a progressive investigation of ovule, gametophyte, embryo and endosperm development, and demonstrate incomplete penetrance of the apomixis-like phenotype rather than complete sexuality. These findings will be valuable for further investigations aimed at understanding the establishment of apomixis in *Boechera*.

## MATERIALS AND METHODS

*Boechera* (Brassicaceae) is a model genus for cytological and evolutionary research; however, efforts to realize the full potential of this model system have been thwarted by problems with species identification and placement of them in an appropriate evolutionary context (Li *et al.*, 2017). There is substantial progress in identifying the sexual and apomictic *Boecher*a diploids, thanks to in-depth morphological, cytological (cytogenetics and embryology) and molecular analyses, with special emphasis on microsatellite data.

A summary of the methods used in this study is included in Supplementary Data Table S1.

### Plant material and growth conditions

Seeds of diploid *B. stricta* lines from North America with the original ES designations ES512 (progeny of SAD12, ES6, Taylor River, a reference line used for crossing and genome sequencing) and ES655 (progeny of ES52, Cloudland; Schranz *et al*., 2005, 2007) were obtained from the Sharbel laboratory (IPK Gatersleben). Seeds of *B. stricta* LTM (a reference sexual line for microsatellite data) were obtained from Tom Mitchell-Olds (Department of Biology, Duke University, USA). The plants (Supplementary Data Fig. S1) were grown from the original seeds and two generations were used for the experiments. Seeds were placed on moist filter paper in Petri dishes, cold-treated at 4 °C for 3 weeks in the dark and then transferred to pots containing soil mixed with sand. Plants from each accession were separated from one another to minimize any chance of cross-pollination by adjacent plants, although *Boechera* is highly self-fertile. Twenty plants of ES655 and ES512 accessions and five plants of LTM were grown in a temperature-controlled greenhouse at 18/20 °C (night/day) under a long-day regime, with a 16-h photoperiod under a photon flux density of 100 μmol m^−2^ s^-1^ and 65 % humidity. On day 28, the plants were vernalized for 6 weeks at 4 °C as described by Schranz *et al.* (2005) for flowering and seed setting. Approximately 50 seedlings from each accession were hydroponically cultured (as described by Tocquin *et al.*, 2003) and used for germination tests and chromosome counting.

### Phenotype identification

All specimens included in embryological analyses were assessed for qualitative and quantitative morphological characters that have proved useful for recognizing *B. stricta* (Schranz *et al.*, 2005; Flora of North America Web Site: http://www.efloras.org/; Go Botany: https://gobotany.newenglandwild.org/). Macro- and/or microscopic analyses were performed for leaf trichomes, flower and pollen morphology, number of seeds produced, and seed coloration and size.

### Genotype identification

Total genomic DNA for ES512, ES655 and LTM was extracted from 20 mg of silica-dried leaves (Chase and Hills, 1991) using a DNA kit (FastDNA™ Spin Kit no. 116540600; MP Biomedicals) following the manufacturer’s protocol. Nuclear ribosomal DNA [internal transcribed spacer (ITS) ITS1-5.8S-ITS2], a plastid region containing the *trn*L(UUA) intron and *trn*L(UUA)-*trn*F(UUC) intergenic spacer (IGS) (referred to as *trn*L-F) and 18 microsatellite loci [ICE3, ICE14, a1, a3, b6, c8, e9, BF3, BF9, BF11, BF15, BF18, BF19, BF20, Brdu266 (according to Li *et al.*, 2017); H34/ICE4, d3, H105/SLL2 (Clauss *et al.*, 2002; Dobeš *et al.*, 2004)] were used for molecular identification (Supplementary Data Table S2).

The ITS was amplified using primers 101AB and 102AB (Sun *et al.*, 1994) followed by cloning of primary PCR products into the Easy Clone Jet (Thermo Scientific). The *trn*L(UUA) intron and *trn*L(UUA)-*trn*F(UUC) IGS was amplified using primers c and f according to Taberlet *et al.* (1991).

Microsatellite loci were amplified using a Qiagen Multiplex PCR Master Mix with FAM- and HEX-labelled PCR forward primers (Sigma) according to protocol from Dobeš *et al.* (2004) (H34/ICE4, d3, H105/SLL2, a1, a3, b6, c8, e9), following the PCR conditions for all remaining loci (ICE3, ICE14, BF3, BF9, BF11, BF15, BF18, BF19, BF20, Brdu266): initial denaturation (95 °C, 5 min) 25 × (95 °C, 30 s; 53 °C, 90 s; 72 °C, 30 s) and final extension (60 °C, 30 min). PCRs for ITS and *trn*L-F were carried out in a total volume of 25 μL using DreamTaq PCR Master Mix (2×) (Thermo Fisher Scientific), 0.2 µmol of each primer and 30–60 ng of DNA. Amplification conditions for ITS were 94 °C for 4 min; 30 × (94 °C, 45 s; 52 °C, 45 s; 72 °C, 1 min) and 72 °C for 7 min. Amplification conditions for *trn*L-F were 94 °C for 4 min, 30 × (94 °C, 45 s; 50 °C, 45 s; 72 °C, 1 min) and 72 °C for 7 min. The PCR products were purified using a High Pure PCR Product Purification Kit (Roche Diagnostics, Germany), and bidirectional sequences were generated on an ABI 3720 automated capillary DNA sequencer. Amplicons were sized using the 500 ROX standard on an Applied Biosystems 3730 DNA Analyser. Alleles were determined using GeneMarker 2.7.0 (SoftGenetics, State College, PA, USA).

The *Boechera* Microsatellite Website (http://sites.biology.duke.edu/windhamlab/) and data from Dobeš *et al.* (2004), Clauss *et al.* (2002) and Schranz *et al.* (2007) were used to compare allelic state and confirm parentage.

To test whether apomixis-specific polymorphisms within the *APOLLO* gene exist, primers Lara 5-F (5′-CCTCATCGTA CCGTTGCTTCTCTC-3′) and TSP1-R (5′-GATAGCCCCAAA CTCCAAAATCGC-3′; Corral *et al.*, 2013) were used for the amplification of a short fragment that contains a 20-nucleotide polymorphic site specific to apomicts. All sequences have been submitted to GenBank under the following reference numbers: KY807649, KY807650, KY807651, KY807652, KY807653 and KY807654.

### Chromosome counting

Young anthers, pistils, shoots from original plants and 14-d-old *F*_1_ seedlings (i.e. seedlings from the first generation of the seeds produced by tested plants) were used to count chromosomes at mitotic (seedlings, roots and sporophytic tissues of stamens) and meiotic (microsporocytes) stages. Samples were fixed in a mixture of 99.8 % ethanol and glacial acetic acid (3:1) and stained according to standard methods with aceto-orcein (Jensen, 1962), Feulgen’s stain (Fukui and Nakayama, 1996) or DAPI (4′,6-diamidino-2-phenylindole) (Ross *et al.*, 1996). Squashed preparations were analysed under a 100× objective and bright-field (Feulgen and aceto-orcein) or UV light (DAPI) with an Epi-Fl Filter Block N UV-2A (EX 330-380, DM 400, BA 435-485).

### Flower analysis

To evaluate megasporogenesis and megagametogenesis, flower buds were divided into three categories: (1) small flower buds [very tightly closed, containing green petals and short stamens (Supplementary Data Fig. S2C)]; (2) mid-size flower buds (tightly closed, containing green petals and light green closed anthers, which were below the stigma); and (3) large flower buds (with milky petals and yellow-green anthers that remained below the stigma) (Supplementary Data Fig. S2A, B). The flower bud stages corresponded to stages 2 (I–V) and 3 (I–VI) of ovule development, as described by Schneitz *et al.* (1995) for *Arabidopsis*.

To evaluate microsporogenesis, very young flower buds were used (stages S3–S11 according to Mau *et al.*, 2013), and pollen grain formation was assessed in flower buds at the same stage as for ovules.

To assess the modes of reproduction, we performed two types of experiment, which were initiated 48 h before anthesis (48 HBA, mid-size flower bud stage). Unpollinated flower buds of randomly selected individuals were marked and either (1) left on the inflorescence for open pollination (in fact self-pollination was the most probable as *B. stricta* is highly self-fertile) to evaluate seed formation ability, or (2) emasculated and isolated against external pollination (using hand-made, half-translucent paper mini-bags) to evaluate autonomous apomixis. Emasculation was performed using a desktop magnifier with a backlight (Newbrand, Transfer Multisort Elektronik, Łódź, Poland) and a stereo microscope (Nikon SMZ 1500).

Megasporogenesis and gametophyte development were checked for 72–24 HBA flower buds. To assess seed development, we analysed flower buds/flowers from 24 HBA up to 7 d after anthesis (DAA) and during the following stages of silique growth until maturity. Emasculated flower buds were examined on the day of emasculation (48–24 HBA) and on days 3 and 7 of the experiment (DAE). The examinations at 3 or 7 DAE corresponded to ~48 h after anthesis (HAA) and 6 DAA, respectively.

### Paraffin sections and tissue clearing technique

Material fixed in acetic alcohol (glacial acetic acid:100 % methanol 1:3; 24 h) was embedded in paraffin, sectioned and stained as described by Rojek *et al.* (2005). For cleared samples, the procedure given by J. Bohdanowicz (unpubl. res.) was applied. After fixation in acetic alcohol, the material was dehydrated [pure methanol for 15 min followed by acidified 2,2-di-methoxypropane (DMP; Sigma-Aldrich, Poland) for 2–4 h], then pre-incubated in DMP:propylene oxide (Sigma-Aldrich) solutions (3:1 and 1:3, v/v) and finally in pure propylene oxide (15 min per step). Samples were then incubated in a propylene oxide:cedar oil (Merck) mixture (10:1 v/v; in closed Eppendorf tubes for 1 h, followed by open Eppendorf tubes overnight for propylene oxide evaporation). Ovules/seeds were isolated from cleared flower buds/flowers/young siliques in a drop of pure cedar oil, mounted under a coverslip and examined with differential interference contrast (DIC) optics.

### Callose detection

Decolorized aniline blue (DAB, Polyscience C.I. 42755) was used to detect the presence of callose in the ovules at the sporogenesis and gametogenesis stages, as described previously by Smith and McCully (1978) and Musiał *et al.* (2015), with modification. After incubation in 1 N NaOH (3 h at 37 °C) and washing in distilled water, the young flower buds were stained overnight with 0.05 % DAB in 0.1 m KH_3_PO_4_. The flower buds were then placed in 0.1 m KH_3_PO_4_, and the ovules were dissected and observed under UV light using a Nikon Eclipse E 800 microscope with an Epi-Fl Filter Block N UV-2A (EX 330-380, DM 400, BA 435-485).

### Ultrastructural analysis

The procedure for preparing the samples for transmission electron microscopy was as described earlier (Kozieradzka-Kiszkurno *et al.*, 2011). The material was dehydrated in a series of graded acetone and embedded in Spurr’s low-viscosity resin (Polysciences, Germany). Semi-thin sections (0.5–1 µm) were stained with toluidine blue and mounted with DPX (Sigma, Poland). Ultrathin (60–100 nm) sections were cut with a diamond knife on a Leica EM UC7 ultramicrotome. The sections were stained with uranyl acetate and lead citrate and then viewed using a Philips CM100 transmission electron microscope in the Faculty of Biology, University of Gdańsk (Poland).

### Immunocytochemical analysis

Flower buds and flowers were prepared as described by Krawczyk *et al.* (2016). After dehydration, material was embedded in Steedman’s wax and sectioned at 2–5 µm. Microtubules were visualized using a rat primary antibody against α-tubulin (Ab6161; Abcam, UK) and an anti-rat secondary antibody conjugated with DyLight™ 549 (AS12 2084; Thermo Fisher Scientific). The chromatin of the nuclei was stained with 7 µg ml^−l^ DAPI (Sigma-Aldrich).

Rat monoclonal antibody JIM13 (Plant Probes, UK) was used to recognize arabinogalactan proteins (here, detection of the trisaccharide β-d-glucose A-β (1→3)-d-galactose A-α (1→2)-l-rhamnose; Knox *et al*., 1991; Yates *et al*., 1996). Ten randomly selected small flower buds of both lines were sectioned to 2–5 µm and incubated with JIM13 (dilution 1/20 in PBS). After washes in PBS, the sections were incubated with anti-rat secondary antibody conjugated to FITC (Ab6840, Abcam; dilution 1/500 in PBS). In negative control experiments, the primary or secondary antibodies were omitted. The sections were mounted under coverslips with Mowiol medium and viewed under an epifluorescence microscope.

### Seed viability

To evaluate *F*_1_ (first generation of the seeds produced by tested plants) seed viability, seeds from ten siliques of each accession were counted and used for the tetrazolium chloride test (Supplementary Data Materials and methods S1).

### Flow cytometric seed screening

Two hundred randomly selected seeds from at least five plants per accession were analysed to determine single-seed embryo and endosperm ploidies, using a high-throughput flow cytometric seed screening (FCSS) method (Aliyu *et al.*, 2010) and a conventional method (Matzk *et al.*, 2000) with modification (Supplementary Data Materials and methods S2).

### Statistics

Megasporogenesis was examined embryologically in ~1000 ovules from each line, and additionally 1369 ovules were used for callose detection. Megagametogenesis was assessed in ~500 ovules from each line. For microsporogenesis and pollen analysis, ~50 flower buds of each line were used. A total of 733 seeds of LTM, 934 seeds of ES655 and 1357 seeds of ES512 were assessed for embryo and endosperm development, and corresponding statistics are provided. To determine the occurrence of apomixis, ten (ES655) or 15 (ES512) emasculated flower buds were used for embryological analysis. All calculations were performed in Excel (Microsoft Office package) using standard procedures.

### Image collection and processing

Photographs of flowers and seeds were taken under a Nikon SMZ 1500 stereoscopic microscope equipped with digital DS-Fi1 camera (Precoptic, Warsaw, Poland). Photomicrographs of the paraffin or cleared and immunostained sections were taken with a Nikon Eclipse E800 epifluorescence microscope equipped with DIC optics and a Nikon DS-5Mc CCD camera (Precoptic), or with a fully automated upright fluorescent microscope (Leica DM6000 B). Ultrathin sections were viewed using a Philips CM100 transmission electron microscope. All figures were analysed in Adobe Photoshop (Elements 11 and CS6 versions).

## RESULTS

### Genotype identification confirmed *B. stricta* features

Principal genotype identification was done based on microsatellite identification, using LTM as reference. The analysis of 18 microsatellites (Supplementary Data Table S3) revealed that ES512 and LTM were homozygous at all loci, while ES655 was homozygous at 17 (94 %) loci, the exception being the heterozygous a3 locus. The status of *B. stricta* was confirmed by TESLA allele analysis (with filter for sexual diploids; i.e. heterozygosity <0.5), and with the top hit for LTM being *B. stricta* (with no filter), while ES512 and ES655 had top hits for *B. fendleri* × *B. stricta* and *B. fendleri* × *B. spatifolia* × *B. stricta*, respectively.

Because ES655 and ES512 have so far been little described, ITS, chloroplast DNA (cpDNA) haplotype and the *APOLLO* gene were verified in both genotypes. There were no differences between nuclear ITS sequences between the ES512 and ES655 lineages, both were homozygous, and a comparison with ITS sequences from other *Boechera* species (Koch *et al.*, 2003; Dobeš *et al.*, 2004) confirmed the chloroplast haplotype L.

Alignment of our sequences with the *trn*L intron and *trn*L*-trn*F intergenic spacer data of Dobeš *et al.* (2004) and Schranz *et al.* (2005) indicated that ES512 has haplotype DG and ES655 has haplotype BF, and both are from *B. stricta* lineage II (Schranz *et al.*, 2005).

The ES655 and ES512 lines proved to be homozygous for sex alleles of the *APOLLO* gene (Corral *et al.*, 2013).

Chromosome counting indicated that all tested lines were diploid: 2*n* = 2*x* = 14 (Supplementary Data Fig. S3).

### Phenotypic features distinguished the examined lines

The plants differed in the number and arrangement of sessile and two-rayed (malpighiaceous) trichomes of basal leaves (Supplementary Data Fig. S4), and also in the timing of flower development. ES512 grew and blossomed at a slower rate than LTM and ES655, but also showed accelerated flower ageing and smaller siliques (Supplementary Data Figs S1 and S2A–I). The seeds differed in colour and size, from the smallest seeds of ES512 to the largest of LTM. The siliques of each line contained bulging as well as collapsed seeds (Supplementary Data Fig. S2G–I), with collapsed seeds being frequently observed in ES512. ES655 produced 50–100 seeds per silique, similarly to LTM (63–100), while ES512 plants varied with respect to silique and seed number, ranging from those with many siliques having >100 seeds per seed capsule to some individuals producing single siliques with very few viable seeds (Supplementary Data Fig. S5A–G). Tetrazolium chloride tests for seed viability revealed 100 % viable embryos for LTM and 80–90 % viable embryos in both ES655 and ES512 lines. However one or two specimens per ES-line generation were characterized by lower viability and underdeveloped embryos (Supplementary Data Fig. S6). The assessment of *F*_1_ seed germination revealed almost 100 % viable seedlings in the LTM and ES655 lines, in contrast to ≤60 % viable seedlings in ES512 plants (data not shown).

### Ovule development

While examining the *B. stricta* lines in more detail, three steps of ovule development were considered: (1) megasporogenesis, starting from MMC differentiation in the archespore, and finishing with megaspore formation and FM establishment (FM being a transition stage between sporo- and gametogenesis); (2) gametogenesis, development of the embryo sac from FM to mature FG establishment; and (3) embryo and endosperm development (seed development).

The flower buds contained ovules in the stages ranging from archespore formation to FG maturity. The most noticeable differences between genotypes were observed during megasporogenesis and involved the delay or acceleration of meiotic events and integument growth, with the most variability observed in ES512 (Supplementary Data Table S4). The megasporocytes, dyads and several triad and tetrad variants were common for all genotypes. In general, integument growth accompanied subsequent sporogenic events, from inner and outer integument initials at the MMC stage to integuments almost covering the nucellus at the tetrad stage. However, desynchronization of these two simultaneous processes was marked in ES512 (Supplementary Data Table S4). Gametophyte development appeared to be undisturbed and similar between the lines, although undeveloped ovules without an FG were sporadically observed in ES512.

### Megaspore formation

**Fig. 2.**
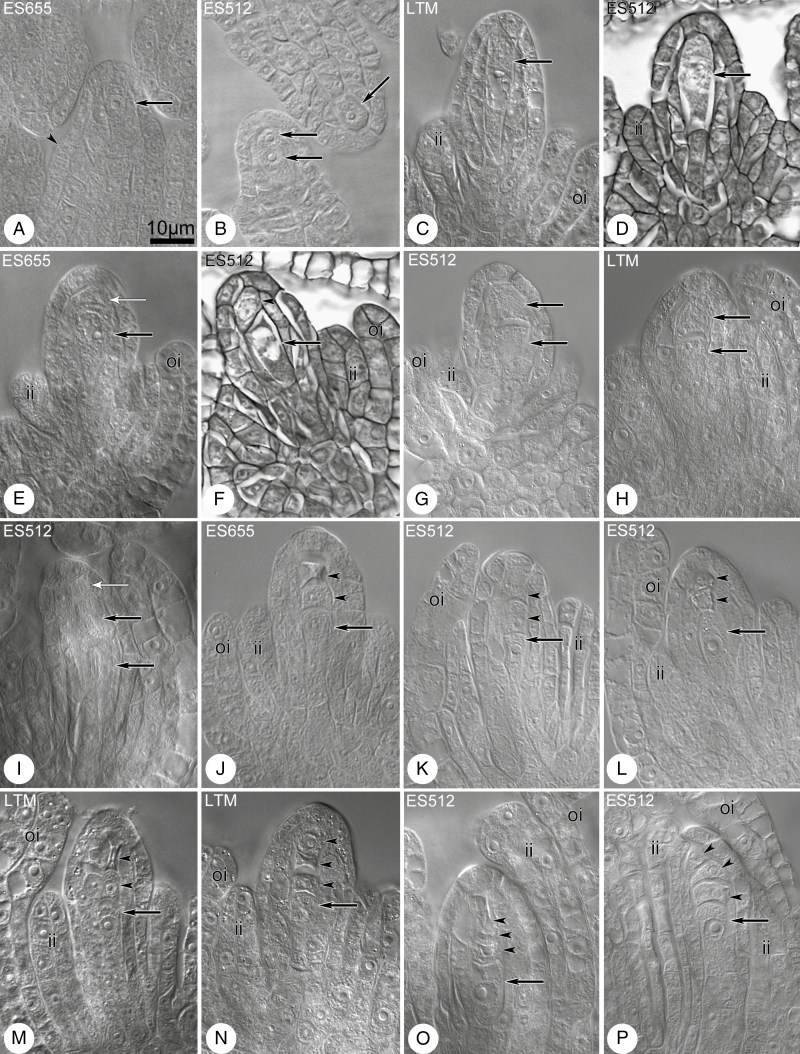
Megasporogenesis in diploid *B. stricta* lines. Cleared material visualized by DIC microscopy. (A) Ovule at stage of MMC differentiation from the archespore (arrow) in the subepidermal region. The inner integument begins to appear (arrowhead). (B) Ovule primordia with a single archespore cell (one arrow) or two-celled archespore (two arrows) in the subepidermal region. (C, D) The MMCs (arrows) in young ovules. The inner integument has been initiated and grows towards the megasporocyte. (E) The MMC (black arrow) below the parietal cell (arrow with white head). (F) The MMC (arrow) accompanied by parietal cell (arrowhead) or at dyad stage. (G–I) Dyad stage. Black arrows indicate cells of the dyad and white arrow indicates the parietal cell. (J–M) Triad and tetrad stages. Arrows indicate the chalazal and enlarged megaspore and arrowheads indicate its sister cell and degenerated micropylar megaspore(s). (N–P) Tetrad stage. The chalazal megaspore is enlarged, in contrast to the remaining small and misshapen megaspores (arrowheads). In ES512, the inner and outer integuments almost enclose the nucellus (O, P). Abbreviations: ii, inner integument; oi, outer integument. Scale bar in (A) applies to all images.

**Fig. 3.**
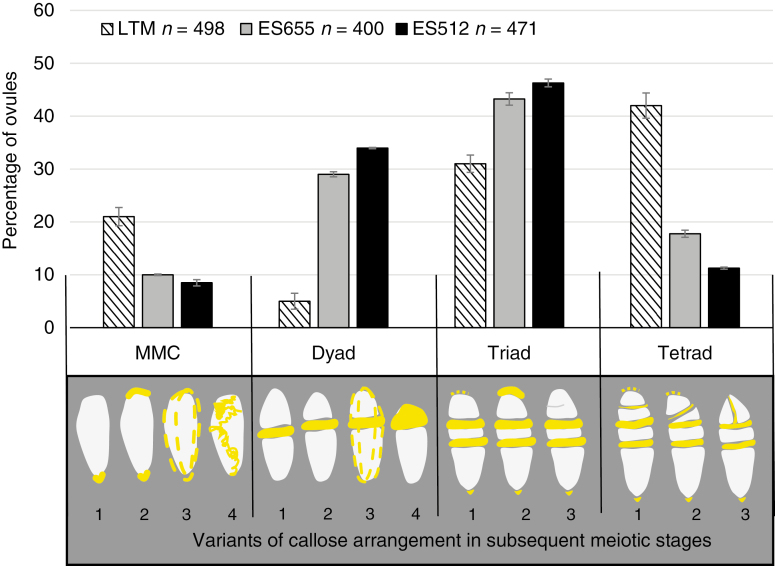
Summary graph of the frequency of subsequent sporogenic stages in LTM, ES655 and ES512 ovules. Bars represent the standard error. *n*, number of ovules analysed. The categories for the meiotic figures are presented below the graph; callose is indicated in yellow. (MMC stage) Callose marks only the micropylar and/or chalazal pole (1, 2), or is punctate in the whole cell wall (3). Irregularly arranged callose deposits (4). (Dyad stage) Callose plate between two cells of the symmetrical (1) and asymmetrical (2) dyad. There is a thick callose plate between two cells of the asymmetrical dyad and discrete callose deposits in the outer walls (3). Callose over the chalazal cell of the dyad, with a degenerated micropylar cell (4). (Triad stage) There is a thick and extensive callose plate between the micropylar megaspore and the two chalazal sister cells (1–3). The chalazal megaspore is enlarged; the second plate that forms after meiosis II contains less callose. Callose deposits were frequently noted at the chalazal and micropylar pole of the triad. The micropylar cell remains undivided (1, 2) or division is unfinished (3). (Tetrad stage) Callose plates separate all megaspores of the linear (1) and T-shaped (3) or irregular (2) tetrad. The first callose plate is thicker and more extensive in comparison with those that are formed subsequently. The callose plate between the two micropylar megaspores is weak and frequently invisible.

**Fig. 4.**
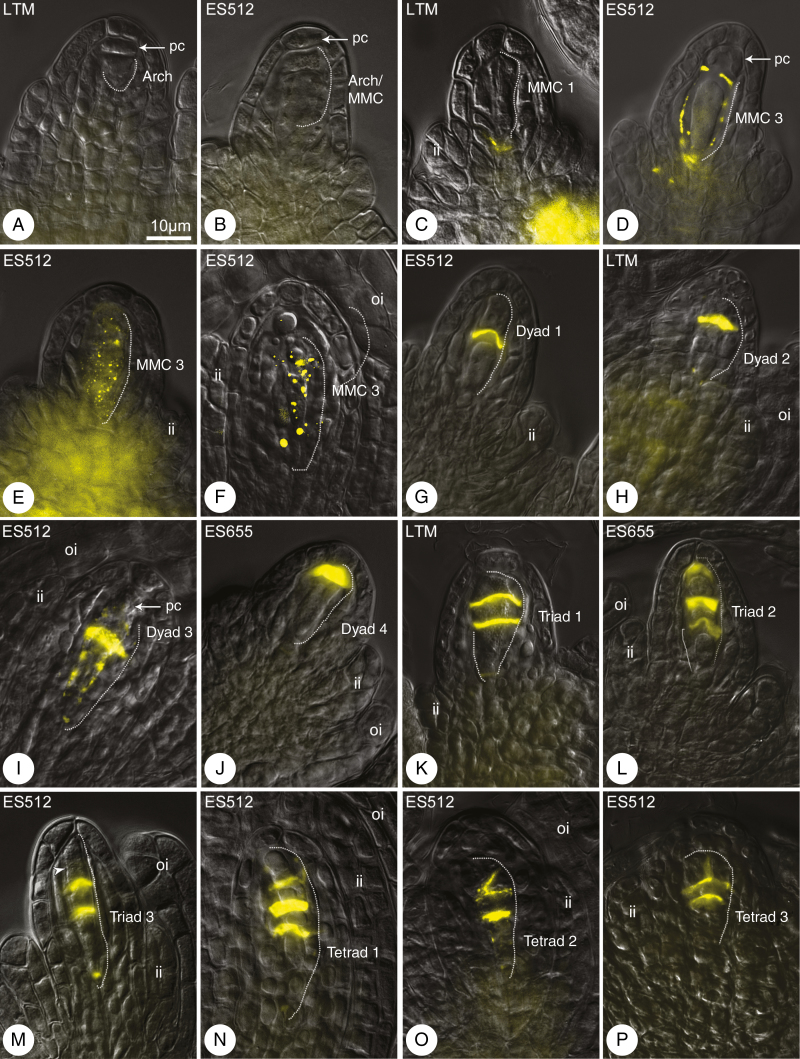
Callose distribution during megasporogenesis. Callose was detected by aniline blue staining (yellow). Merged differential interference contrast microscopy images and DAB detection. Nomenclature of meiotic figures corresponds to categories in Fig. 3. (A, B) Archespore/MMCs lack callose; only a weak callose signal was detected in sporophytic cells of the ovule. (C–F) Callose patterns during the MMC stage, at the base of the MMC (C) or around the MMC (D–F). (G–J) Dyad stage. Callose plaque between equal (G) and unequal (H, I) cells of the dyad. (J) Callose over the chalazal cell of the dyad, in the place of degenerated micropylar cell. (K, L) Triads. Callose plates between the chalazal sister cells and the micropylar cell. (M) Triad/tetrad. A putative third cell wall between micropylar megaspores is free from callose (arrowhead). (N–P) Three types of tetrad: linear (N), irregular, with the cell wall arranged diagonally between micropylar cells (O), or T-shaped (P). The variable rate of integument development is shown, from weak formation (M, P) to almost covering the nucellus (N, O). Abbreviations: arch, archespore cell; ii, inner integument; oi, outer integument; pc, parietal cell. The MMC and following cells are outlined by a dashed line. Scale bar in (A) applies to all images.

Megasporogenesis was preceded by differentiation of the MMC in the archespore, immediately following the differentiation of inner and outer integuments (Fig. 2A, B). The archesporial cell developed directly into the MMC (Fig. 2C, D) or divided to produce the MMC and a parietal cell (Fig. 2E, F). The microtubular cytoskeleton was rich, exhibiting a dense network (Supplementary Data Fig. S7). The meiocyte stage was short in some ovules, and two daughter cells were frequently observed, whereas others remained enlarged but undivided despite the significant progress in ovule development (Fig. 4F). The cells of the dyad were approximately equal in size (Fig. 2G–I), or the chalazal cell was significantly larger than the micropylar one, with the micropylar cell in the dyad showing signs of degeneration (Figs 3 and 4H–J). Several dyads were still visible in late-stage ovules, where integuments almost covered the nucellus (Fig. 4I) (Supplementary Data Fig. S8I). Triads of megaspores were linear in variants containing all viable cells or dead micropylar cells (Fig. 2J–M). The chalazal megaspore of the tetrad (Fig. 2N–P) was most frequently enlarged and became the FM. Variation in integument development was clearly visible at triad and tetrad stages, from triads/tetrads noted in early-stage ovules (Figs 2J and 4K–L; Supplementary Data Fig. S8J, K, M, N) to those occurring in late-stage ovules (Figs 2P and 4N, O; Supplementary Data Fig. S8L, O).

Callose detection revealed the establishment of the MMC cell wall and its derivatives during megasporogenesis (Fig. 3). No callose signal was detected for the archespore at the meiocyte differentiation stage (Fig. 4A, B), though callose was readily detectable during the MMC stage (Fig. 4C–F). Meiocytes exhibited a few patterns of callose: unipolar or bipolar callose (Fig. 3, MMC 1 and 2) (Supplementary Data Fig. S8A–B), the first of which reflected callose being distributed mostly at the chalazal end (Fig. 4A); around the MMC (Fig. 3, MMC 3; Fig. 4D–F) (Supplementary Data Fig. S8C, D) or irregular patterns were observed (Fig. 3, MMC 4) (Supplementary Data Fig. S8E). At the dyad stage, two cells of the dyad were separated by a callose plaque (Fig. 3, dyads 1–3; Fig. 4G–I) (Supplementary Data Fig. S8F, G), and the dyad cells could be surrounded (Fig. 3, dyad 3; Fig. 4I) (Supplementary Data Fig. S8G) or a thick callose cap covered the micropylar cell (Fig. 3, dyad 4; Fig. 4J) (Supplementary Data Fig. S8H). A very few MMCs and dyads of ES512 were callose-free (Supplementary Data Fig. S8I).

The distribution of callose demonstrated that triads were as frequent as tetrads at the end of sporogenesis (Fig. 3). Three patterns of triads were observed, even within a single pistil. In some, a thick plate of callose was noted in the transverse walls separating each megaspore, and a spotted callose layer was observed at the triad poles (Fig. 3, triad 1; Fig. 4K; Supplementary Data Fig. S8J–K). Other triads exhibited a callose cap encompassing the uppermost micropylar cell (Fig. 3, triad 2; Fig. 4L), while the micropylar cell of other triads was elongated and/or looked partly divided (Fig. 3, triad 3; Fig. 4M). Tetrads occurred at the same time as triads. The parietal cell was sometimes still visible above the uppermost micropylar megaspore (Supplementary Data Fig. S8O). Tetrad variants followed the arrangement of the uppermost callose plaque (Fig. 3), which was usually weakly visualized and arranged approximately parallel to two other plaques (Fig. 3 tetrad 1; Fig. 4N; Supplementary Data Fig. S8L–N), diagonally (Fig. 3, tetrad 2; Fig. 4O; Supplementary Data Fig. S8O), or even transversely to form T-shaped tetrads (Fig. 3, tetrad 3; Fig. 4P; Supplementary Data Fig. S8P). The chalazal megaspore of the triad or tetrad was usually larger and elongated and was labelled by a transient or sustained, but spotted, distribution of callose (Fig. 3).

**Fig. 5.**
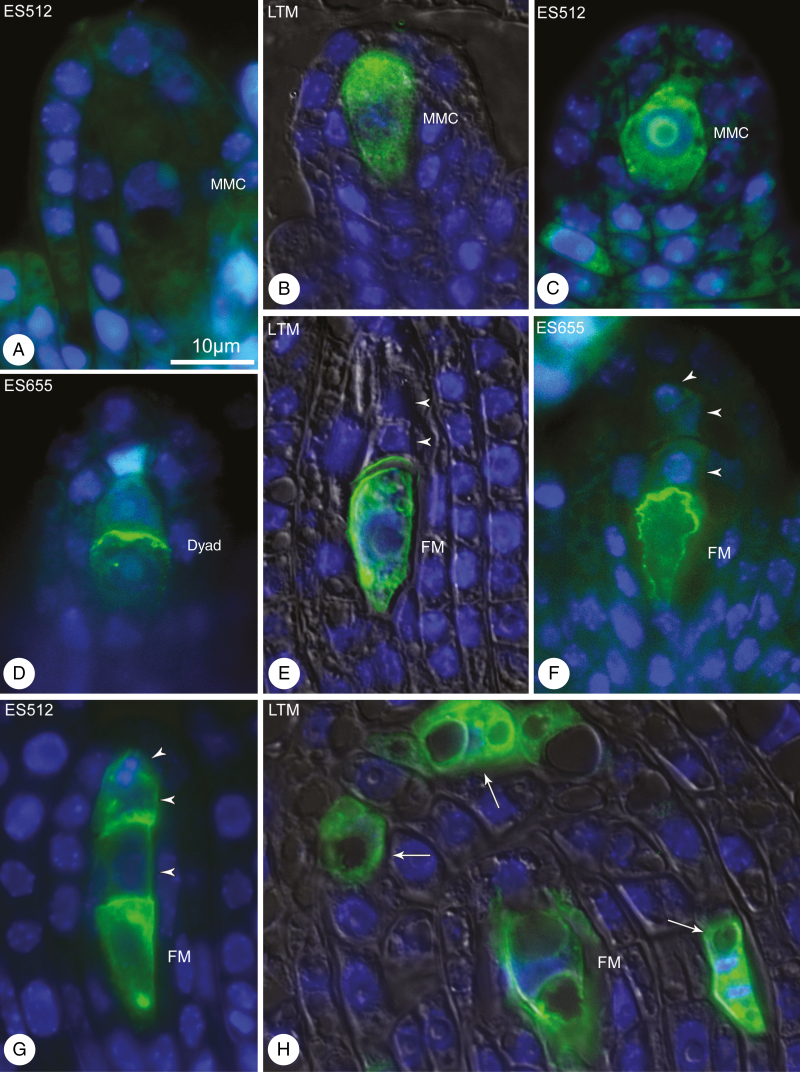
JIM13 immunodetection in *B. stricta* ovules during megasporogenesis. (A–C) MMC stage. Young meiocytes lacking (A) or showing (B, C) localization of JIM13. (A) Meiocyte that is significantly enlarged but unlabelled. (D) Dyad stage. Epitopes recognized by JIM13 are specifically labelled at the top of the chalazal cell. (F) Postmeiotic ovule showing JIM13 localization at the surface of the chalazal megaspore (E, F) or of all the resulting megaspores (G). (H) JIM13 at the surface of the functional megaspore and several integument cells (arrows). Nuclei are stained blue (DAPI) and JIM13 is stained green (FITC). Merged DIC, DAPI and FITC images for (B), (E) and (H). Scale bar in (A) applies to all images.

To determine which cells (formed via sporogenesis) would develop further into an FM and eventually an FG, the localization of the AGP-related sugar epitope recognized by JIM13 was assessed in the ovules of randomly selected pistils. JIM13 was not detected in the archespore and many MMCs (Fig. 5A). However, several MMCs in ES512 and LTM were characterized by JIM13 localized on the cell surface and inside the cell (Fig. 5B, C). JIM13 was initially localized at the surface of the chalazal cells of some dyads in ES655 ovules (Fig. 5D), whereas towards the end of megasporogenesis JIM13 was localized to the region surrounding the chalazal megaspore (Fig. 5E, F) or observed at the surface of one, two or all resulting megaspores (Fig. 5G), with additional JIM13 localization in one or more sporophytic (integumental) cells (Fig. 5H).

### Megagametogenesis

**Fig. 6.**
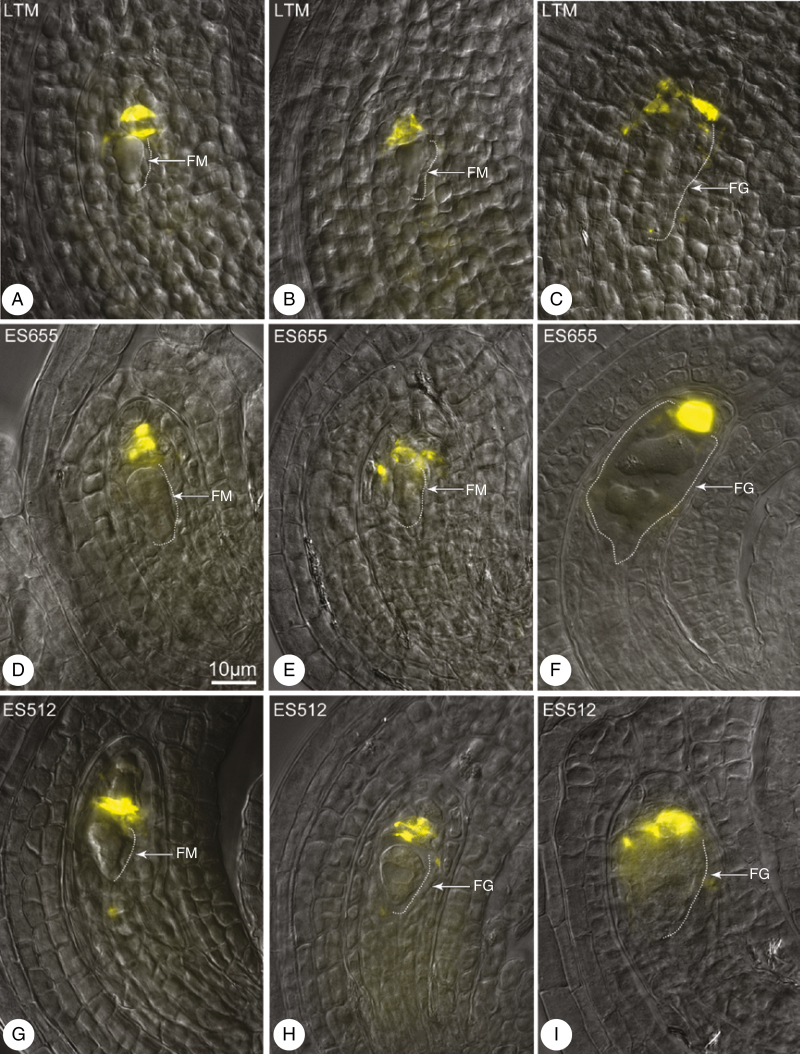
Callose arrangement at the end of megasporogenesis (A, D, G) and during megagametogenesis (B, C, E, F, H, I) in LTM (A–C), ES655 (D–F) and ES512 (G–I). Merged DIC microscopy images and DAB detection. (A, B, D–F, G) Callose is above the functional megaspore (FM) (A, B, D, E) or above the female gametophyte (FG) (C, F, H, I). Callose was detected by aniline blue staining (yellow). The FM and FG are outlined by a thin dashed line (on the right). Scale bar in (D) applies to all images.

The FMs were usually free from callose, which could be detected above or on the top of the FM (Fig. 6A, B, D, E, G), where it remained during gametophyte developmental stages (Fig. 6C, F, H, I).

**Fig. 7.**
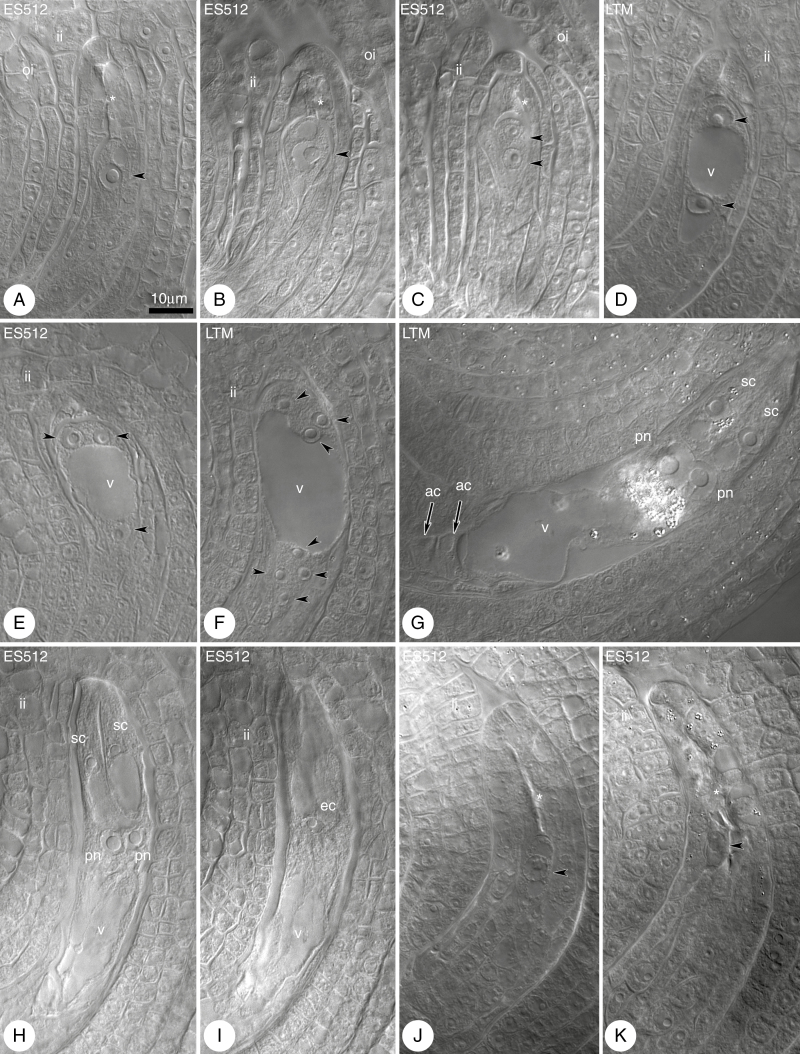
Megagametogenesis in *B. stricta*. Cleared material visualized by DIC microscopy. (A, B) Late functional megaspore (FM; arrowhead) stage. Scars from degenerated megaspores are marked by an asterisk. (C, D) Two-nucleate female gametophyte (FG); nuclei of the FG (arrowheads) are separated by the vacuole (v) and the integuments have almost enclosed the nucellus. The asterisk indicates a scar from degenerated megaspores. (E) Four-nucleate embryo sac; nuclei (three of the four nuclei are indicated by arrowheads) are distributed at the micropylar and chalazal poles. (F) Eight-nucleate embryo sac; nuclei (arrowheads, with one micropylar nucleus invisible) are distributed at the micropylar and chalazal poles. (G) Seven-celled embryo sac. The egg apparatus consists of the egg cell (invisible) and two synergids accompanied by two polar nuclei (pn) and three antipodal cells (ac) (arrows indicate two visible antipodal cells). (H, I) Mature four-celled FG with two polar nuclei, synergids (H) and an egg cell (ec) (I). The nucellus is completely enclosed by the integuments. (J, K) Examples of the ovules with FM (arrowheads) arrested in development (J) or degenerated (K). Abbreviations: ii, inner integument; oi, outer integument; sc, synergid cell; Scale bar in (A) applies to all images.

Development of the FG had begun at the time of FM vacuolation, resulting in the formation of an FG with a single nucleus (Fig. 7A, B). The FM/single-nucleate FG was located at the most proximal position with respect to the placental attachment of the ovule to the gynoecia, with extensive scarring of the dead megaspores being observed above the FM/single-nucleate FG (Fig. 7A). However, single-nucleate FGs located nearer to the nucellus epidermis were sometimes noted (Fig. 7B).

The first mitotic division and the formation of an FG with two nuclei (Fig. 7C, D) were usually correlated with a completely developed integument that enclosed the nucellus. Undisturbed gametogenesis proceeded in most ovules, leading to the formation of mature FGs with a *Polygonum*-type arrangement (Fig. 7G–I). Subsequent stages of gametogenesis were very similar in all lines. The two and then four nuclei of the FG were arranged in a 1 + 1 (Fig. 7D) and 2 + 2 pattern (Fig. 7E), respectively, and separated by a central vacuole, followed by an eight-nucleate stage with a 4 + 4 pattern (Fig. 7F), which changed to a 5 + 3 pattern at the stage of cellularization (not shown).

At maturity, the embryo sac was seven-celled (Fig. 4G). The cells of the egg apparatus were enriched in microtubules, which displayed a longitudinal pattern in synergids and a hooplike cortical system in egg cells, and formed a radial system around the polar nuclei or the central cell nucleus (Supplementary Data Fig. S9A–C). Antipodal cells disappeared when the FG was ready to be fertilized. At this stage, two polar nuclei adhered to the egg apparatus (Fig. 7H, I). Polar nuclei were visible for a long period, with occasional fusion and secondary central cell nucleus formation. Some ovules of ES512 contained undeveloped or degenerated FGs (Fig. 7J, K).

Ultrastructure analysis was performed for the two ‘East’ *Boechera* accessions since no embryo sac disturbances in LTM were found in cleared material. The ultrastructure of the young embryo sac was similar in the two tested lines (ES655 and ES512) (Supplementary Data Figs S10 and S11); however, some differences were recognized. Many lipid droplets and plastids showing starch accumulation were characteristic of the ES512 embryo sac (Supplementary Data Fig. S11A–C) but were rarely noted in ES655 (Supplementary Data Fig. S10A, B). Numerous mitochondria were observed, exhibiting two forms: small and ellipsoidal (Supplementary Data Fig. S11B) or enlarged and cup-shaped (Supplementary Data Fig. S10B). During gametogenesis, nucellus degeneration progressed more rapidly in ES655 (Supplementary Data Fig. S10A, B) compared with ES512 (Supplementary Data Fig. S11A, C), and the mature FG almost adhered to the integumentary tapetum (Supplementary Data Fig. S10F). In ES512 ovules, a thin layer of the nucellus was still visible at the time of FG maturity (Supplementary Data Fig. S11D). Growth of the integumentary tapetum was correlated with the progress of gametogenesis (Supplementary Data Figs S10A, D and S11A, C), with the cells of this structure accumulating increasing amounts of starch in plastids (Supplementary Data Fig. S10D). Synergid cells were fully developed with numerous mitochondria, rough endoplasmic reticulum, dictyosomes and vesicles (Supplementary Data Figs S10C, E, F and S11D–F). Synergidal plastids accumulated starch in ES512 (Supplementary Data Fig. S11E) and were starch-free in ES655 (Supplementary Data Fig. S10F), while the filiform apparatus of the synergids was strongly expanded (Supplementary Data Figs S10F and S11D, F).

### Microsporogenesis and pollen development

Microspore formation was characterized by a number of observations: (1) microsporocytes surrounded by callose layer which went through meiosis and generated microspores exhibiting tetrahedral tetrad formation (Supplementary Data Fig. S12A–E, G, I); (2) triads noted in all genotypes being in fact tetrads where the fourth cell was hidden; and (3) abnormalities including micronuclei or additional nuclei in dyad or triad cells instead of tetrads (LTM), degenerated microspores in the tetrads (ES655 and ES512), and, more rarely, empty anthers (all accessions, approximately once per 50 flower buds) (J. Rojek *et al*., unpubl. res.). Meiotic chromosome non-disjunction was observed once in ES512 sporogenic tissue and was accompanied by C-mitotic figures in tapetal cells (J. Rojek *et al*., unpubl. res.). Microspores/pollen grains with one or two nuclei formed in the anthers of small flower buds (Supplementary Data Fig. S12F, H). In mid-sized flower buds, the anthers had not yet dehisced and contained mostly two-nucleate pollen grains (Supplementary Data Fig. S12J–L, O). Large flower buds contained a mixture of pollen grains with two or three nuclei, and numerous dead pollen grains, particularly in ES512 (Supplementary Data Fig. S12M, N, P). Collapsed as well as bulging, ellipsoid grains with symmetrical colpi occurred during anther dehiscence of opened flowers (Supplementary Data Fig. S12R–W). In a few extreme cases, ES512 plants were highly sterile and contained anthers that never dehisced (Supplementary Data Fig. S5A, B).

### Seed development

**Fig. 8.**
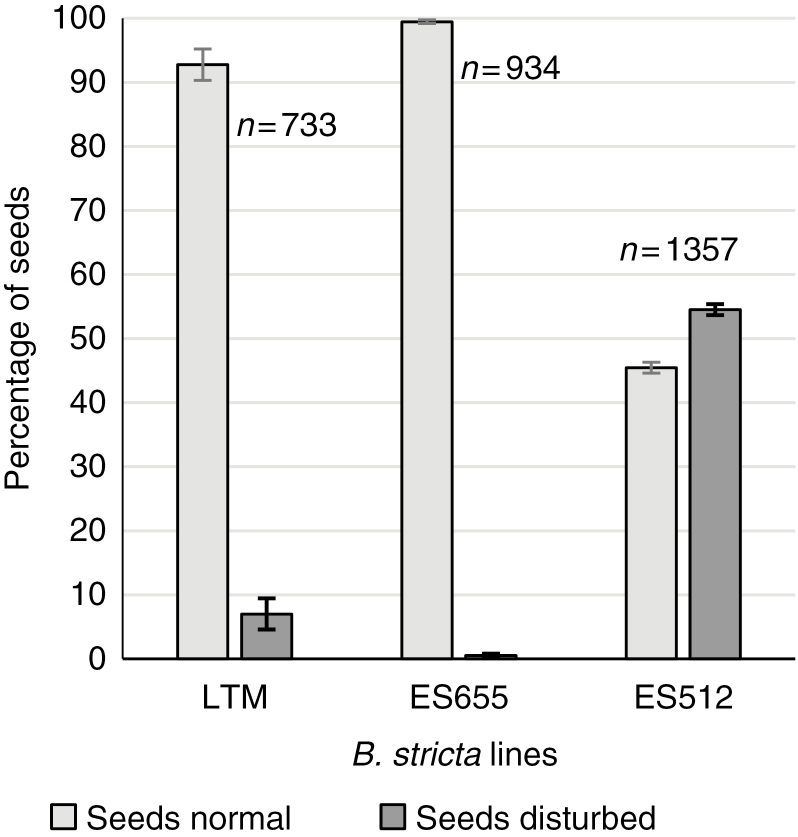
Summary of normal and disrupted seed formation after open pollination in *B. stricta* lines. A significant percentage of deviation from correct seed formation is characteristic of ES512. Bars represent standard error; *n*, number of seeds analysed.

While almost all of the ES655 and LTM seeds developed correctly, the frequencies of normal and disrupted seeds were similar in ES512 (45.5 % normal, 54.5 % disrupted; Fig. 8). The mature siliques contained both enlarged viable and aborted seeds (Supplementary Data Figs S2G–I and S5).

**Fig. 9.**
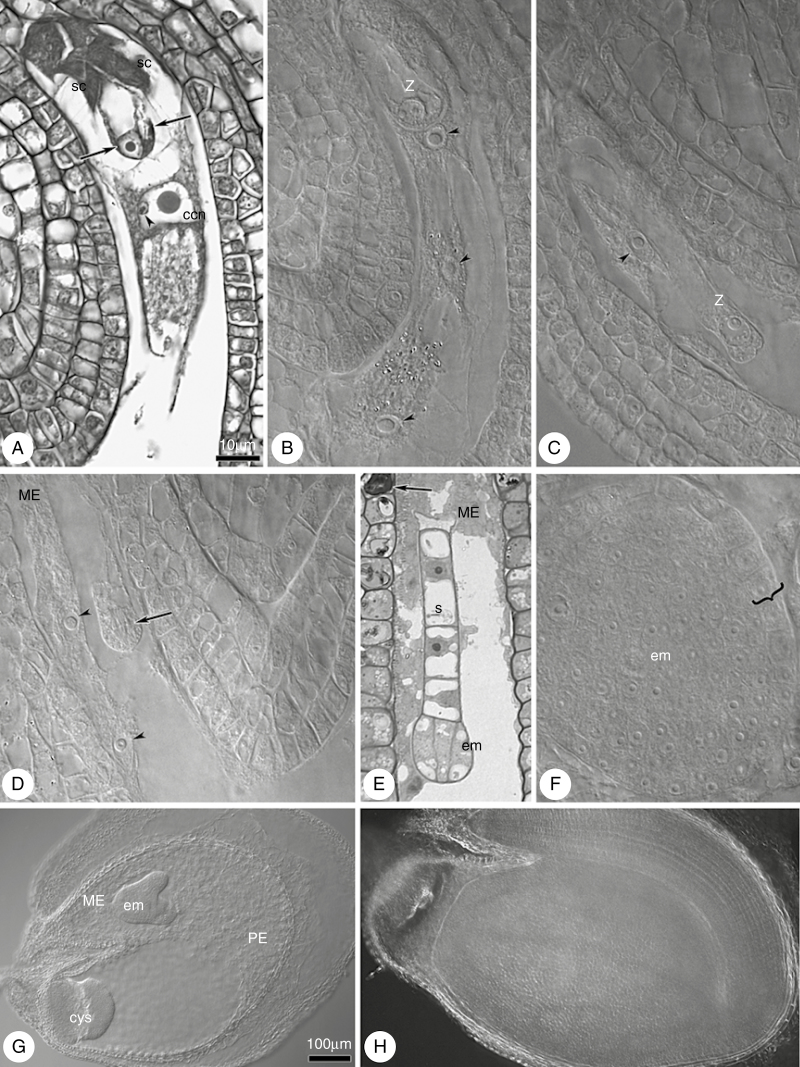
Embryogenesis in the ES655 line. (A) At the time of double fertilization. Two nuclei (arrows) in the egg cell; fusion of central cell nucleus with sperm nucleus (arrowhead); synergid degeneration. (B, C) Early stages of the zygote and endosperm development (arrowheads). (D) Mitotic division in the zygote (arrow) and free-nuclear endosperm; endosperm nuclei indicated by arrowheads. (E) Four-cell proembryo with few suspensor cells. The embryo is surrounded by micropylar endosperm and the integumental cells accumulate tannins (arrow). (F) Late globular embryo; the protoderm is distinct (curly bracket). (G) Heart stage of the embryo. The endosperm is divided into three regions: cellularized micropylar region; peripheral region, where the nuclei are situated parietally and around the central vacuole; and the chalazal region, where the nuclei, together with cytoplasm, formed a cyst. (H) Mature embryo with the cotyledons filling the embryo sac; endosperm is completely absorbed. Abbreviations: sc, synergid cell; z, zygote; ccn, central cell nucleus; em, embryo; s, suspensor; cys, chalazal cyst; ME, micropylar endosperm; PE, peripheral endosperm. (A) Paraffin section; (E) semi-thin section; (B–D, F–H) cleared material visualized by DIC microscopy. Scale bar in (A) applies to (A–F); scale bar in G applies to (G, H).

**Fig. 10.**
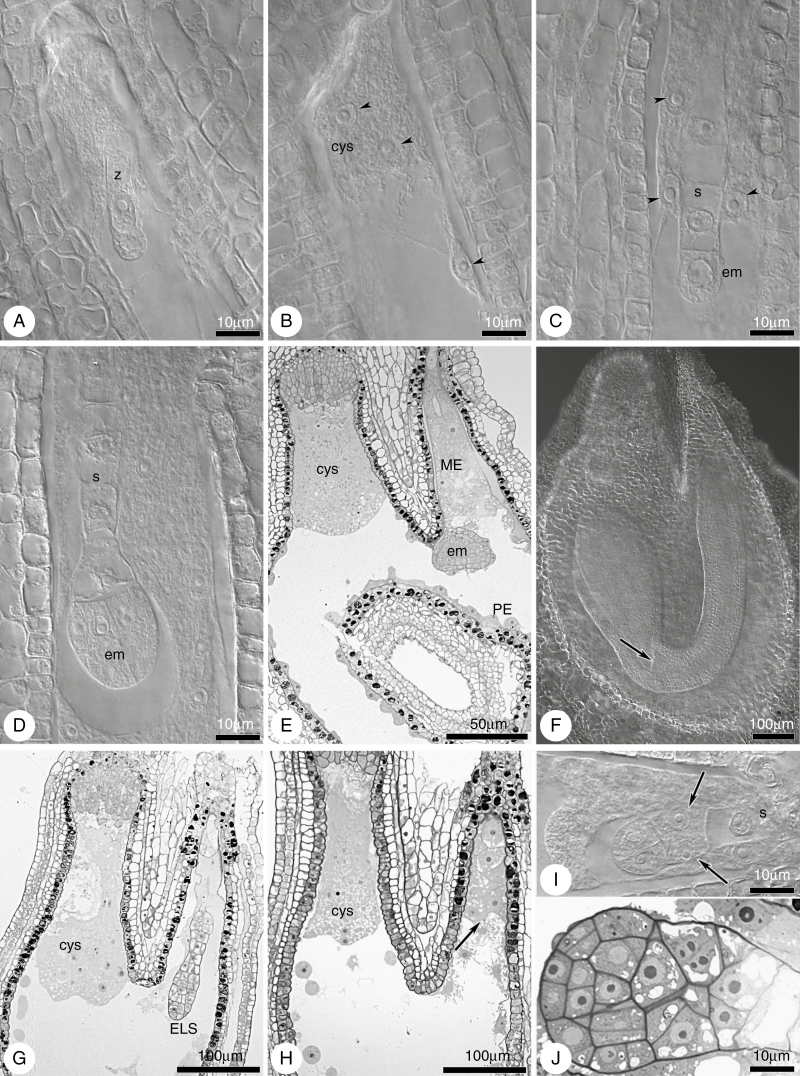
Embryogenesis in the ES512 line. (A–F) Normal embryo and endosperm development. (A) Zygote and endosperm with several nuclei. (B, C) Two-celled proper embryo with a few suspensor cells and free-nuclear endosperm; endosperm nuclei indicated by arrowheads. (D) Octant stage of the embryo and multinuclear endosperm development. (E) Early heart stage of the embryo. Endosperm shows distinct regions; micropylar, peripheral and chalazal endosperm forms an extensive cyst. (F) Walking-stick embryo with cotyledons and a shoot meristem (arrow); disappearance of the suspensor; initiation of endosperm degeneration. (G–J) Examples of abnormalities during seed development. (G) Embryo-like structure showing deviation from cell specification: overproliferation of an endosperm cyst at chalazal pole. (H) Endosperm development without embryo development (arrow). (I, J) Disturbances in boundary (arrows) formation between the embryo proper and the suspensor. Abbreviations: z, zygote; em, embryo; s, suspensor; cys, chalazal cyst; ME, micropylar endosperm; PE, peripheral endosperm; ELS, embryo-like structure. (A–D, F, I) Cleared material visualized by DIC microscopy; (E, G, H, J) semi-thin sections.

Fertilization (Fig. 9A) (Supplementary Data Fig. S13A) was noted in several ovules, between 0 and 24 HAA. The endosperm developed earlier than the embryo, and displayed few or more nuclei at the zygote stage (Figs 9B–D and 10A, B; Supplementary Data Fig. S13B). At zygote elongation or division, the endosperm progressed and became multinuclear (Supplementary Data Fig. S13C, D). A normally developed globular embryo proper and suspensor were noted in almost all young seeds examined (~3–4 DAA). The subsequent stages of embryo development (Figs 9E–H and 10B–D; Supplementary Data Fig. S13C–F) followed the Onagrad type of embryogenesis (e.g. Czapik and Izmaiłow, 2001). Cellularization of the coenocyte during the heart stage of embryo development (~6–7 DAA) resulted in division of the endosperm into three characteristic regions: the micropylar cellular endosperm surrounding the embryo; the peripheral endosperm with the nuclei situated parietally around the central vacuole; and the chalazal endosperm forming a chalazal cyst (Figs 9G and 10E; Supplementary Data Fig. S13F). In mature seed, the cellular endosperm was absorbed by the fully developed embryo (Figs 9H and 10F). The arrangement of the microtubular cytoskeleton was similar in ES655 and ES512 embryo sacs after fertilization (Supplementary Data Fig. S9D–I).

Since we did not find any disturbances in LTM seed development, but noted differences between two ‘East’ *Boechera* accessions, the ultrastructure of LTM was omitted. The ultrastructure of young ES655 seeds showed that the suspensor cells were highly vacuolated and contained mostly spherical or elongated thylakoid-free plastids (Supplementary Data Fig. S14A). The cytoplasm of the micropylar and chalazal endosperm was rich in smooth endoplasmic reticulum, mitochondria and plastids with well-developed thylakoid structures (Supplementary Data Fig. S14B–D). The chalazal endosperm was still nuclear in the late heart stage of embryo development, with variform nuclei, few mitochondria and thylakoid-filled plastids (Supplementary Data Fig. S14D). The cells of the micropylar endosperm were vacuolated, contained one or two nuclei and accumulated starch in plastids (Supplementary Data Fig. S14E).

**Fig. 11.**
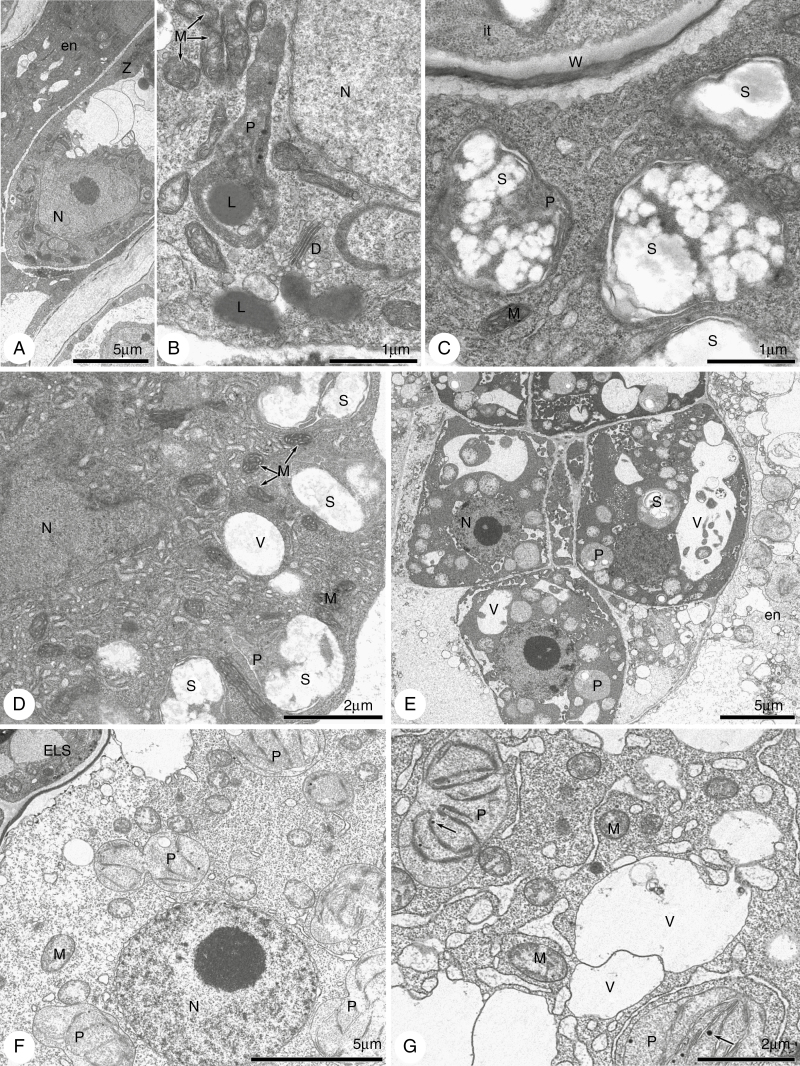
Electron micrographs of embryogenesis in line ES512. (A–D) A zygote and nuclear endosperm. Zygote cytoplasm (A, B) is dense and rich in lipids and long plastids without thylakoids, but accumulates lipids. The micropylar (C) and chalazal (D) cytoplasm of the nuclear endosperm is also dense and rich in organelles, especially in plastids accumulating huge popcorn-like starch grains. (E–G) Abnormal embryo and endosperm. (E) The irregularly positioned cells of the embryo have dark cytoplasm with non-typical starch-accumulating plastids and autophagic-like vacuoles. The micropylar endosperm (E, F) is degenerate and diluted, with disappearance of the organelles. The chalazal endosperm (G) is also diluted and fragmented but still contains mitochondria and spherical plastids with thylakoids. P, plastid; S, starch; V, vacuole; L, lipids; W, cell wall; N, nucleus; D, dictyosomes; en, endosperm; it, integumentary tapetum; z, zygote; M, mitochondria; ELS, embryo-like structure.

Approximately 45 % of the ES512 seeds developed normally, while ~55 % (Fig. 8) were disrupted. Abnormalities occurred as early as the embryonic stages consisting of only a few cells, when the endosperm overproliferated and created a huge chalazal cyst, filling the space in the embryo-free micropylar region (Fig. 10G, H). Disruptions at the suspensor–embryo proper boundary, in addition to cell specification and arrangement, were clearly visible (Fig. 10I, J). Abnormalities were also noted at the ultrastructural level, although early developmental stages were not altered. The cytoplasm of the zygote and minimal nuclear endosperm was dense and enriched with organelles, with the endosperm plastids being characterized by the accumulation of huge popcorn-like starch grains (Fig. 11A, D). Abnormal embryos exhibited irregularly positioned cells containing dark cytoplasm with non-typical starch-accumulating plastids and autophagic-like vacuoles (Fig. 11E). The endosperm of such disrupted seeds was degenerate, less dense and lacking organelles (Fig. 11F, G).

### Flow cytometric analyses of seed formation and reproductive mode

**Fig. 12.**
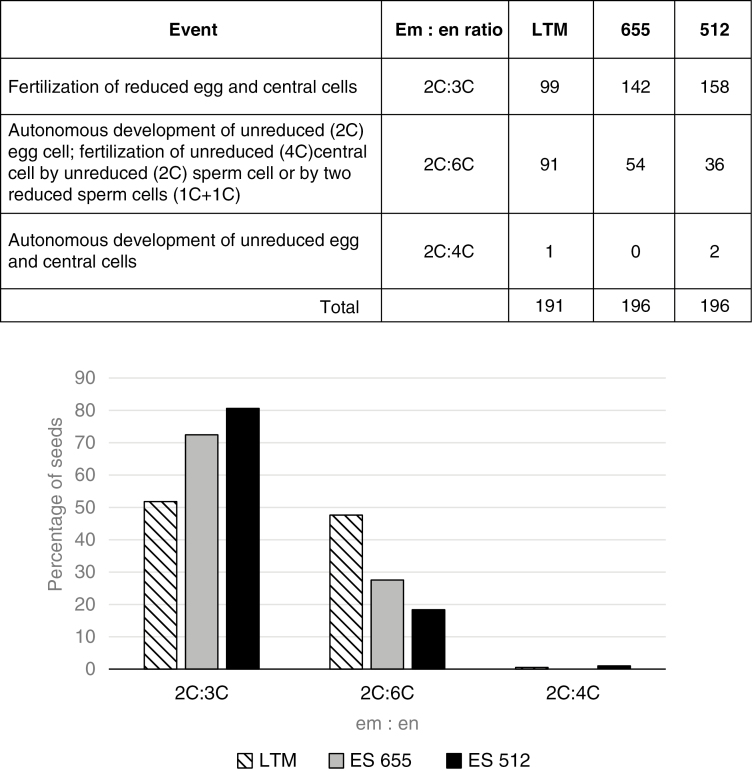
Flow cytometry seed screening from diploid *B. stricta* accessions. Combined razor-chopping and high-throughput FCSS results are presented. Occurrences of different embryo:endosperm (Em:en) C-value ratios were counted for LTM, ES655 and ES512 seeds. The graph below the table summarizes the average frequency of the ratios within and between genotypes.

In total, 583 seeds from the three diploid genotypes were measured using flow cytometry. Overall (68 % of all seeds), the expected ratio of 2C:3C (embryo:endosperm) for sexual reproduction occurred in the studied genotypes; however, the remaining 32 % of seeds were characterized by non-sexual ratios (2C:6C and 2C:4C; Fig. 12). The 2C:3C ratio was more frequent in ES655 and ES512, while LTM had similar frequencies of seeds with 2C:3C and 2C:6C ratios. The 2C:4C ratio (i.e. apomictic with autonomous endosperm formation) was identified in a single seed of LTM and in two seeds of ES512. Additionally, some histograms revealed the presence of endopolyploid (8C and 12C) cells (Supplementary Data Fig. S15).

### Putative parthenogenesis

**Fig. 13.**
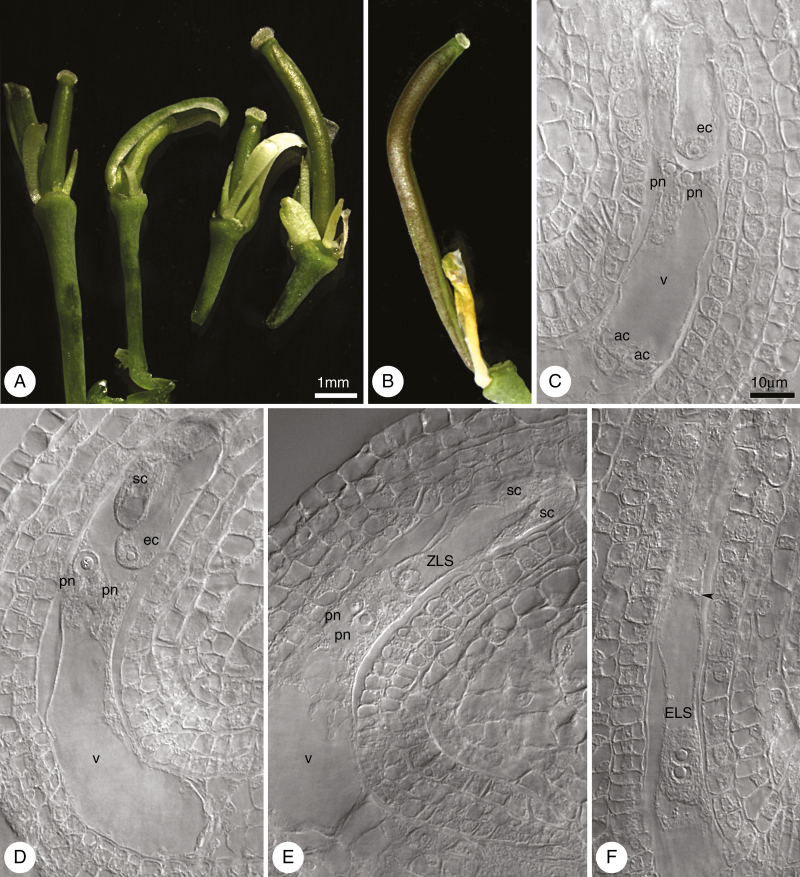
Development of ES512 unpollinated pistils after emasculation and isolation from external pollination. (A) Unpollinated pistils on the third day of the experiment (3 DAE). (B) Enlarged unpollinated pistil at 7 DAE. (C–F) Types of unfertilized embryo sac inside unpollinated but enlarged pistils at 7 DAE. (C, D) Mature embryo sacs consist of intact cells of the egg apparatus: the egg cell and two synergids accompanied by two unfused polar nuclei. Sometimes antipodal cells (C) were noted. (E) Structure resembling the zygote, accompanied by unfused polar nuclei and degenerated synergids. (F) Embryo sac containing an embryo-like structure, which consists of three cells; the boundary between two-nuclear apical cell of the embryo-like structure and other cells is indicated by an arrowhead. Abbreviations: ec, egg cell; pn, polar nucleus; ac, antipodal cell; sc, synergid cell; v, vacuole; ZLS, zygote-like structure; ELS, embryo-like structure. (A, B) Images obtained by stereoscopic microscopy; (C–F) cleared material visualized by DIC microscopy. Scale bar in (A) applies to (A) and (B); Scale bar in (C) applies to (C–F).

To ascertain whether the pistils and ovules of *Boechera* lines developed in the absence of pollination and fertilization, we emasculated mid-sized flower buds and isolated them against external pollination and observed them for 7 DAE. As LTM is known to be an obligate sexual, the experiment was performed for ES655 and ES512 exclusively. We found that 20 % of the ES655 pistils and 80 % of the ES512 pistils showed growth (Fig. 13A, B; Supplementary Data Fig. S16A), morphologically similar to that of pollinated pistils. At 3 DAE, almost all of the ovules of the enlarged pistils contained an FG, at stages ranging from four nuclei to maturity (data not shown). Despite pistil (ovary) enlargement at 7 DAE, many ovules were aborted but some were viable (Supplementary Data Fig. S16B) and exhibited an intact FG (Fig. 13C, D) or central cell only. Very few ES512 ovules contained autonomously formed structures. These structures resembled a zygote (ZLS in Fig. 13E) or an embryo with few cells (ELS in Fig. 13F). Autonomous endosperm with a few nuclei, unaccompanied by an ELS, was observed in one ovule (data not shown). Embryo sacs bearing a ZLS, ELS or autonomous endosperm were enlarged and formed bent, horseshoe-like structures that resembled embryo sacs after fertilization *in planta*.

## DISCUSSION

Here we provide evidence of diversity in the reproduction of *B. stricta*, a model for obligate sexual reproduction in *Boechera* and a genus that is characterized by its widespread distribution and varying levels of apomixis. Indeed, our results showed that the sexual pathway via meiosis and fertilization was present in three *B. stricta* accessions. Additionally, however, we identified deviations from normal patterns of megasporogenesis, variation in embryo:endosperm ratios, low pollen viability (in one line) and disturbances in seed formation and parthenogenesis potential, which together suggest an underlying propensity for apomictic reproduction.

### Hints of both sexuality and apomixis in diploid *B. stricta*

Our results indicate that the seed parent of both lineages ES512 and ES655 was *B*. *stricta*, considering that chloroplast haplotypes DG and BF are restricted to *B. stricta*. Additionally, haplotype DG is very rare and was characteristic of the SAD12 line of *B. stricta*. SAD12 is a tester line (seed parent), highly inbred and sexual, and used for intra- and interspecific crosses within *Boechera* (Schranz *et al.*, 2005). ES512 is the progeny of SAD12 and ES655 is the progeny of ES52 (Schranz *et al.*, 2005).

Analyses of chromatograms and cloned ITS sequences do not demonstrate any evidence for hybridization. Due to concerted evolution of rDNA, ITS is not a suitable marker for hybridization detection within old lineages, although in young hybrids both parental alleles can be observed (Szlachetko *et al.*, 2017). Both lineages (ES512 and ES655) were homozygous for this locus, and our results are consistent with those of Koch *et al.* (2003), in which most *B*. *stricta* accessions were homozygous for ITS. Type L of ITS was also characteristic of 18 specimens of *B*. *stricta*, 19 specimens of *B*. × *divaricarpa* and two specimens of *B*. *holboellii* (Koch *et al.*, 2003). Virtually complete homozygosity in the microsatellite data (Supplementary Data Table S3) also implies that selfing is the most common form of reproduction in these genotypes.

Analysis of the *APOLLO* gene in ES655 and ES512 demonstrated no apomixis-specific 5′-UTR polymorphism (Corral *et al.*, 2013; Mau *et al.*, 2015); rather, both were homozygous for sex alleles, supporting their sexual state and being consistent with Mau *et al.* (2015, dataset S1).

Based on the analysis of 18 microsatellite loci, ES512 and ES655 appear to be ‘pure’ *B*. *stricta.* The a3 locus can exhibit more than the expected number of alleles in plants of known ploidy, and was therefore excluded from heterozygosity calculations (according to Li *et al.*, 2017). Results from the TESLA output further support the pure species (*B. stricta*) status of LTM, while the analysis of ES512 and ES655 suggest the pattern of introgression (e.g. Beck *et al*., 2012; Lovell *et al.*, 2013; Alexander *et al*., 2015; Rushworth, 2015; Windham *et al.*, 2015).

**Fig. 14.**
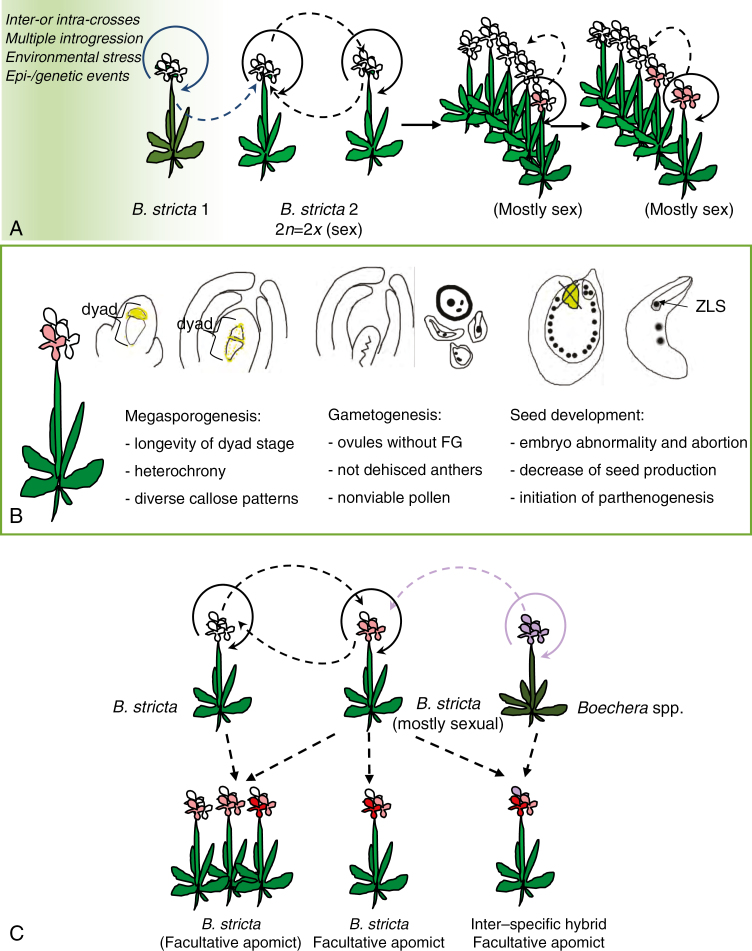
A conceptual model of events during reproduction of diploid *B. stricta*. (A) Despite high self-crossing (circular solid arrows), intraspecific crossing within or between populations could happen (circular dashed arrows). While rare, pollination of sexual diploid plants with reduced pollen that introduces apomixis (epi-)genetic factors could contribute to the ubiquity of diploid apomictic inter- or intra-hybrids across the genus that generated variability. Backcrossing with a *B. stricta* parent makes hybrid-origin plants indistinguishable from pure *B. stricta*. However, other events, such as (epi-)genetic changes, could bring and/or maintain new (here apomictic) features, without hybridity. (B) Differences occurring during the main stages of sexual development were reminiscent of hybrid or apomictic features. During megasporogenesis there is heterochrony in the ovule (integument) growth inside one ovary, and also in the meiotic events (longevity of the MMC and dyad stages), and the presence of many triads. During gametogenesis there is a lack of female gametophyte development with some ovules and non-functional anthers with degenerated pollen grains. During seed development there is disruption of embryogenesis or embryo abortion, a low number of viable seeds, but in some cases parthenogenetic development. (C) New features of *B. stricta* individuals may accumulate (by selfing) and be transferred by intra- or interspecific crossing, leading to the generation of new genotypes. ZLS, zygote-like structure.

The cytogenetic data presented here show the diploid state of the tested plants and, as has been previously shown (Aliyu *et al.*, 2010), evidence for additional apomixis-like processes at varying frequencies (Fig. 12; Aliyu *et al.*, 2010; Sharbel *et al.*, 2010; Voigt-Zielinski *et al.*, 2012). Considering the apparent low levels of apomixis in ES655, ES512 and LTM, it is unclear whether the deviation from sexuality (Fig. 14) represents the normal state of sexual *Boechera*, or whether ancestral introgression and/or (epi-)genetic events have possibly influenced reproduction. These results are congruent with the non-hybrid origin of some diploid apomicts (e.g. Lovell *et al*., 2013, 2017; Rushworth, 2015).

### Heterochrony in ovule development and variability during megasporogenesis provide an opportunity for reduced and unreduced megaspore formation

Genotypic variation in the frequencies of each sporogenic stage reflected desynchronization (or heterochrony) of reproductive development in the tested lines (Figs 2 and 4; Supplementary Data Fig. S8; Supplementary Data Table S4). Differences in developmental rates of the ovules may or may not indicate more than one reproductive mode (i.e. sexual and apomictic) in one plant (e.g. Grimanelli *et al.*, 2003; Carman *et al.*, 2011). Diverse or irregular callose distribution in the walls of some meiocytes may reflect epigenetic influences, as in *Arabidopsis* plants deficient in ACTIN-RELATED PROTEIN, whose requirement in determining the spatial and temporal patterns of gene expression at meiosis has been proven (Qin *et al.*, 2014). Together, MMCs abnormally enlarged but undivided, a long duration of MMC and dyad stages and dyads with only one developed chalazal cell were reminiscent of *Taraxacum*-type spore formation in apomictic *Boechera* (Naumova *et al.*, 2001; Shah *et al.*, 2016).

The accumulation of large amounts of callose in the MMC wall prior to meiotic division and during meiosis is a feature of *Arabidopsis* and other sexual taxa, including basal/early-divergent angiosperms (Lora *et al.*, 2017). After meiosis, callose is detected in non-FMs that will undergo collapse (Tucker and Koltunow, 2014 and references therein) but is degraded around FMs that continue megagametogenesis (Van Hautegem *et al*., 2015). Callose deposition could influence cell–cell communication via plasmodesmata, and probably blocks the exchange of molecules between cells, mostly to the MMC before and during meiosis (Vatén *et al*., 2011; Benitez-Alfonso *et al*., 2013; Han *et al*., 2014). However, callose deposition may not be sufficient to completely block signalling, as in the *somatic and microspore defect 1* (*csmd1*) maize mutant, which undergoes meiosis despite an excess of callose deposition (Wang *et al*., 2011). Here, the phenotypes involving a loss or gain of callose accumulation around megaspores are particularly interesting because of their strong association with the production of polyploid gametes and apomixis (Tucker and Koltunow, 2014 and references therein).

The arrest of cell division of the micropylar dyad was probably responsible for the large number of triads observed. The formation of triads is regarded as a transitional stage, when the chalazal cell of the dyad divides earlier than the micropylar cell (Rodkiewicz, 1970), or as a product of an undivided micropylar dyad cell, which is frequently observed in orchids with a monosporic pattern (Yeung and Law, 2013). Megaspore triads are produced in the sexual diploid *Taraxacum linearisquameum* and in the apomictic triploid *Taraxacum atricapillum* as a result of abnormal meiosis (Musiał *et al.*, 2015), and in apomictic *B. divaricarpa* (Shah *et al.*, 2016) and *B. gunnisoniana* (Schmidt *et al.*, 2014; Shah *et al.*, 2016), where triads may originate from both apomictic and meiotic initial cells. Interestingly, microspore triads are also present at low levels in both sexual and apomictic individuals of *Boechera* (Mau *et al.*, 2013), suggesting that meiotic irregularities are characteristic of the *Boechera* complex.

Our assessment of callose accumulation sites revealed the presence of variability in megaspore tetrad shape, from linear to T-shaped. This makes *Boechera* megasporogenesis even more similar to *Arabidopsis* and *Capsella*, where T-shaped or multishape tetrads, and triads with underdeveloped cell walls between the two uppermost megaspores, are common (Schulz and Jensen, 1986; Schneitz *et al.*, 1995). The meiotic pathway was therefore active and complete, at least in the ovules where tetrad stages had been identified.

In addition to callose, AGPs are cell wall components that exhibit specific localization in female reproductive cells in the developing ovule (Tucker and Koltunow, 2014), and are postulated to serve as a molecular marker of sexual reproduction (Demesa-Arévalo and Vielle-Calzada, 2013). To our knowledge, such signalling events have so far not been investigated in *Boechera*, and hence an explanation of JIM13 localization in LTM, ES655 and ES512 ovules can be made based on *Arabidopsis* and other species data.

Monoclonal JIM8 and JIM13 antibodies that recognize AGP epitopes mark mainly gametophytic cells (especially the FM) in *Arabidopsis* (Coimbra *et al.*, 2007; Lora *et al.*, 2017). In our *B. stricta* lines, the localization of AGPs using JIM13 revealed that more than one postmeiotic megaspore reacted to JIM13, with additional JIM13 signal in single cells of the integuments (Fig. 5). The excess of JIM13 in ovules may come from overexpression of *AGP18*, and is reminiscent of *AGP18*-overexpressing mutants that display abnormal maintenance of the cell during meiosis and additional surviving megaspores that acquire FM identity (Demesa-Arévalo and Vielle-Calzada, 2013). On the other hand, AGP localization can be genotype-specific, since AGPs (JIM8 and JIM13) marked all germ-line cell types during megasporogenesis in early-divergent angiosperms (Lora *et al.*, 2017), with stronger signals in the cell wall of the FM (Lora *et al.*, 2017). Additional JIM13 signal in the integument cells is similar to what has been found in *Arabidopsis* (Coimbra *et al.*, 2007). Single integument cells with AGPs detected by JIM13 may, however, be reminiscent of aposporous initial cells (Tucker *et al.*, 2001; Mateo de Arias, 2015; Carman *et al*., 2011, 2016). The question is open whether specific AGP localization may serve as a universal marker for initial cells (i.e. meiotically reduced as well as non-reduced) that are destined to undergo FG formation.

### Abnormalities in pollen and seed formation limit sexual reproduction, but could provide fodder for apomixis induction

The reproductive variation observed between the lines is not limited to female sporogenesis. Admittedly, we did not detect significant differences in the development of FGs, but we wondered whether the long persistence of polar nuclei and the occasional cases of secondary nucleus formation in mature FGs could be signs of developmental disturbance, as observed in *B. gunnisoniana* (Schmidt *et al.*, 2014). The high rates of abortion and defects in seed development observed in ES512 individuals, as well as the presence of numerous dead siliques prior to ripening (data not shown), may be correlated with the existence of only a small amount of pollen capable of achieving self-pollination.

Embryological and ultrastructural analyses of the embryo and endosperm have confirmed the developmental patterns of most Brassicaceae, especially *Boechera* (e.g. Böcher, 1951; Naumova *et al.*, 2001) and *Capsella* species, in which chloroplasts and starch accumulate to a large extent, whereas a clear decline in the starch and lipid reserves of the central cell is observed during the first few divisions of the primary endosperm nucleus (Schulz and Jensen, 1974). Small differences observed between the tested lines in the ultrastructure of the normal embryo and endosperm (e.g. popcorn-like starch is more characteristic of ES512) were not associated with the observed abnormalities in seed development. A difference was evident in the quantity of viable seeds produced, as those of ES512 germinated at a slower rate with <60 % germination (data not shown).

The high frequency of abnormal seeds in ES512 (seeds without an embryo or with a disrupted embryo, or overproliferation of the endosperm) strongly suggests defective fertilization, as has been found in maturing siliques of *B. gunnisoniana*, in which reproductive development appears to be arrested, leading to the vast majority of mature seeds being derived apomictically (see complete data in Schmidt *et al.*, 2014). Furthermore, seed abortion rates are at higher levels in diploid compared with triploid apomicts, an observation that is consistent with the unmasking of deleterious mutations in the diploids (Voigt-Zielinski *et al.*, 2012).

Autonomous structures were initiated in a few tested ovules and seeds (Fig. 12; Supplementary Data Fig. S16). The true frequencies of autonomous embryo and endosperm formation may be higher than recorded because of seed abortion, as concluded by Aliyu *et al.* (2010) based on single seed analyses. Furthermore, Voigt *et al.* (2007) documented higher frequencies of autonomous endosperm in immature seeds compared with mature ones, in addition to higher frequencies in triploids versus diploid apomicts.

### Do deviations in the sexual pathway of diploid *B. stricta* individuals reflect apomixis initiation?

Although apomixis mechanisms *per se* are better understood, how and when apomixis is acquired during plant development remains mostly unknown. If one assumes that apomixis is activated when specific cells acquire the capability to form apomeiotic spores or embryos, stress (biotic and abiotic), in addition to hybridity and polyploidy, could contribute as a trigger of apomixis (e.g. Hand and Koltunov, 2014; Schmidt *et al.*, 2014, 2015). Stress-induced changes may explain the manifestation of apomixis-like features in a sexual plant, for example in *Arabidopsis* where some genotypes exhibit the elements of apomixis as a result of mutation and/or stress-induced experimental conditions (see reviews by e.g. Tucker and Koltunow, 2009; Grimanelli, 2012; Barcaccia and Albertini, 2013; Hand and Koltunow, 2014). It is unclear whether the low levels of apomictic seed development in these otherwise sexual accessions could have been affected by the environmental conditions in which our genotypes were growing or treatment of the plants with cold stress during development, or whether diploid sexuals have an inherent ability to produce unreduced embryo sacs or pollen (according to Lovell *et al.*, 2013), or whether certain sexual genetic backgrounds have a predisposition for these traits (Aliyu *et al.*, 2010).

We have shown the cell biology of reproduction in diploid *B. stricta* to be sexual, in addition to features of apomictic development being expressed in the absence of polyploidy. The *B. stricta* lines studied here demonstrate instability in their sexuality, a trait that is important for our understanding of the origin of apomixis in *Boechera*. As a promiscuous species involved in the formation of many apomicts, *B. stricta* itself may possess the preadaptation [e.g. encrypted (epi)genetic] marks to become apomictic.

## SUPPLEMENTARY DATA

Supplementary data are available online at https://academic.oup.com/aob and consist of the following. Materials and methods S1: tetrazolium chloride test (TTC) for seed viability. Materials and methods S2: flow cytometric seed screening. Table S1: summary of the main results from the *B. stricta* study. Table S2: list of primer names, labels and sequences used in the simple sequence repeat (SSR) analysis. Table S3: number of alleles observed at 18 microsatellite loci in the analysed accessions. Table S4: meiotic stage occurrence in relation to ovule development. Figure S1: phenotype of tested plants. Figure S2: morphology of flowers and siliques in two diploid lines of *B. stricta*. Figure S3: chromosome counting in original plants and *F*_1_ generation. Figure S4: basal leaf pubescence of *B. stricta*. Figure S5: phenotypic disturbances in reproduction within accession ES512. Figure S6: viability of seeds evaluated by the TTC test. Figure S7: tubulin cytoskeleton – pictures of early megasporogenesis stages. Figure S8: variability in callose arrangement during megasporogenesis. Figure S9: tubulin cytoskeleton pictured around fertilization and embryogenesis. Figure S10: electron micrographs of megagametogenesis in line ES655. Figure S11: electron micrographs of megagametogenesis in line ES512. Figure S12: pollen development. Figure S13: embryogenesis in line LTM. Figure S14: electron micrographs of embryogenesis in line ES655. Figure S15: representative histograms from flow cytometric analysis of nuclei of single-seed samples of diploid *B. stricta*. Figure S16: summary of unpollinated pistil development.

Supplementary Figures

Supplementary Methods

Supplementary Table S1

Supplementary Table S2

Supplementary Table S3

Supplementary Table S4
