# Supplementary material for: Establishing the cell biology of apomictic reproduction in diploid Boechera stricta (Brassicaceae)
Source: Ann Bot. 2018 Jul 6;122(4):513–39. doi: 10.1093/aob/mcy114 (PMC6153484; doi:10.1093/aob/mcy114)
Supplement: Supplementary Figures [file mcy114_suppl_supplementary_figures.pptx]

## Slide 1
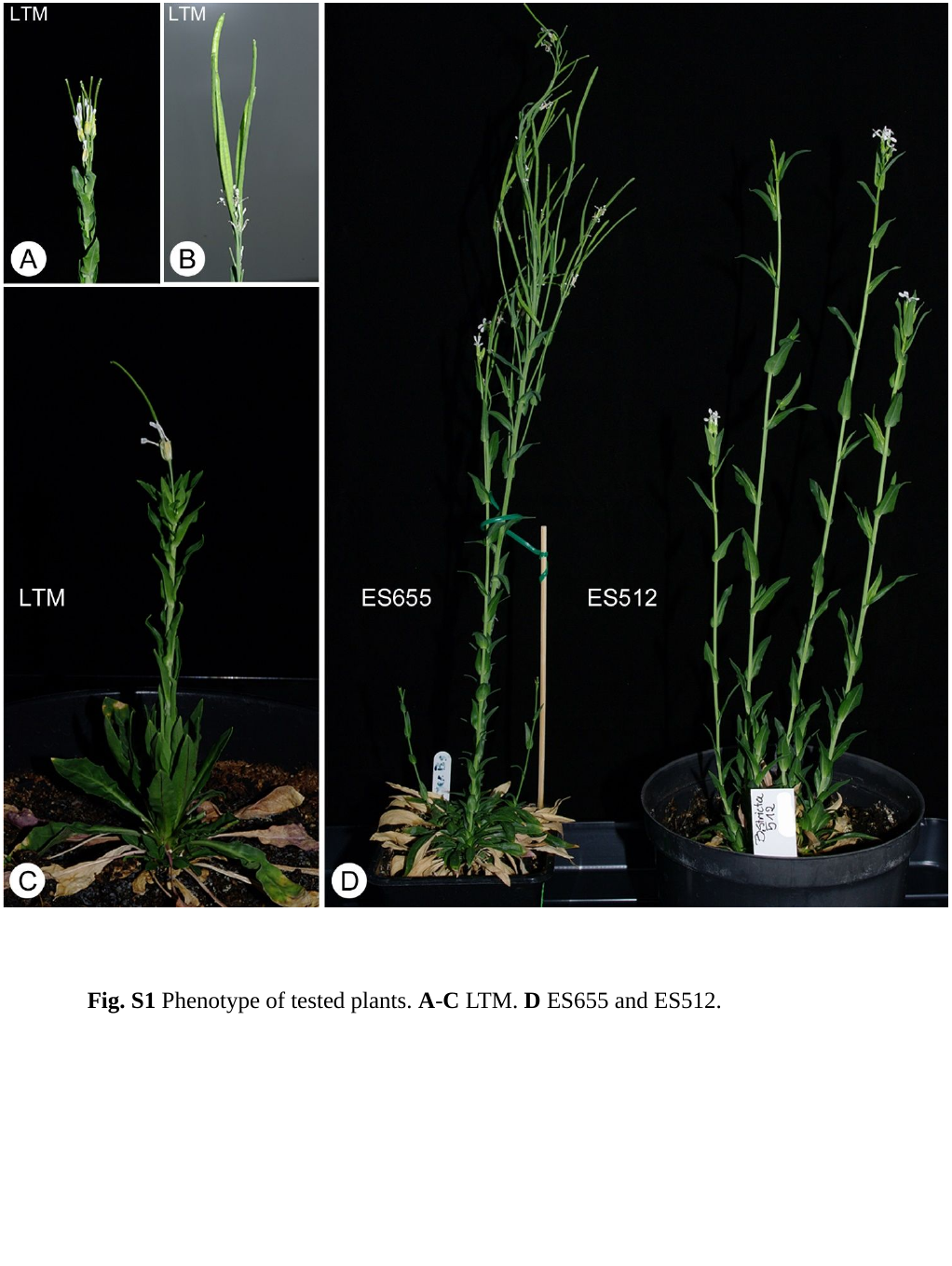

Fig. S1 Phenotype of tested plants. A-C LTM. D ES655 and ES512.

## Slide 2
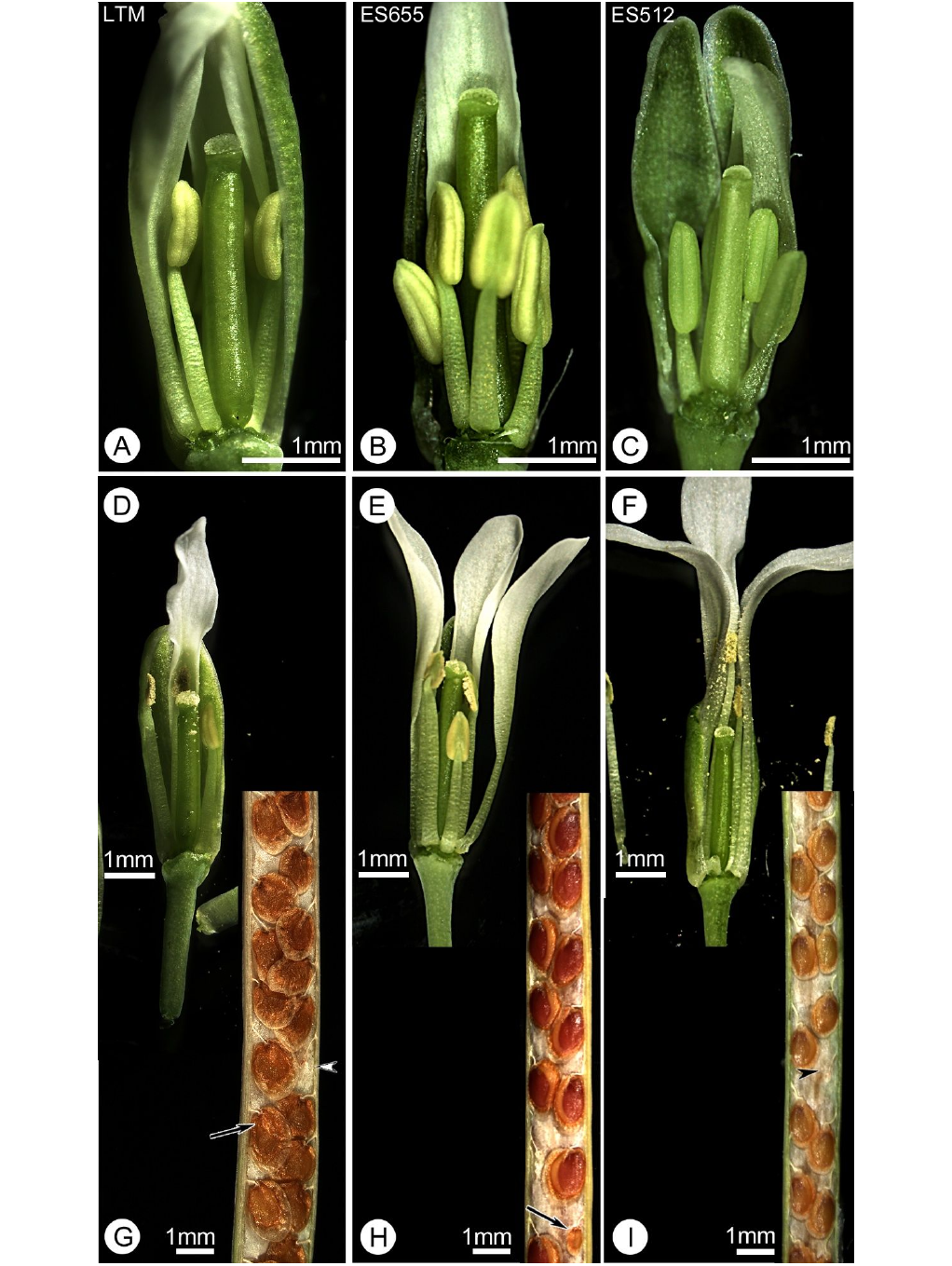

## Slide 3
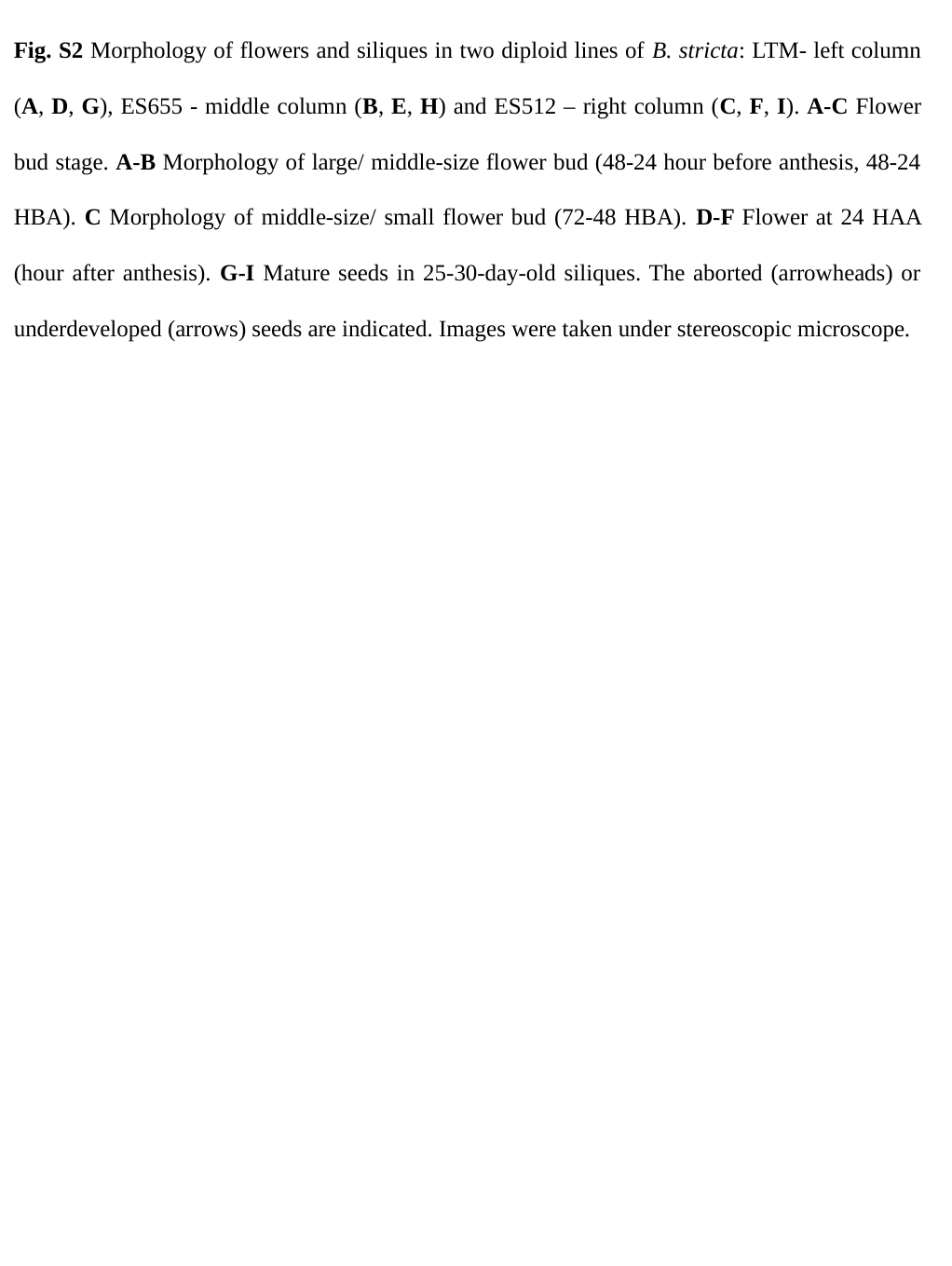

Fig. S2 Morphology of flowers and siliques in two diploid lines of B. stricta: LTM- left column (A, D, G), ES655 - middle column (B, E, H) and ES512 – right column (C, F, I). A-C Flower bud stage. A-B Morphology of large/ middle-size flower bud (48-24 hour before anthesis, 48-24 HBA). C Morphology of middle-size/ small flower bud (72-48 HBA). D-F Flower at 24 HAA (hour after anthesis). G-I Mature seeds in 25-30-day-old siliques. The aborted (arrowheads) or underdeveloped (arrows) seeds are indicated. Images were taken under stereoscopic microscope.

## Slide 4
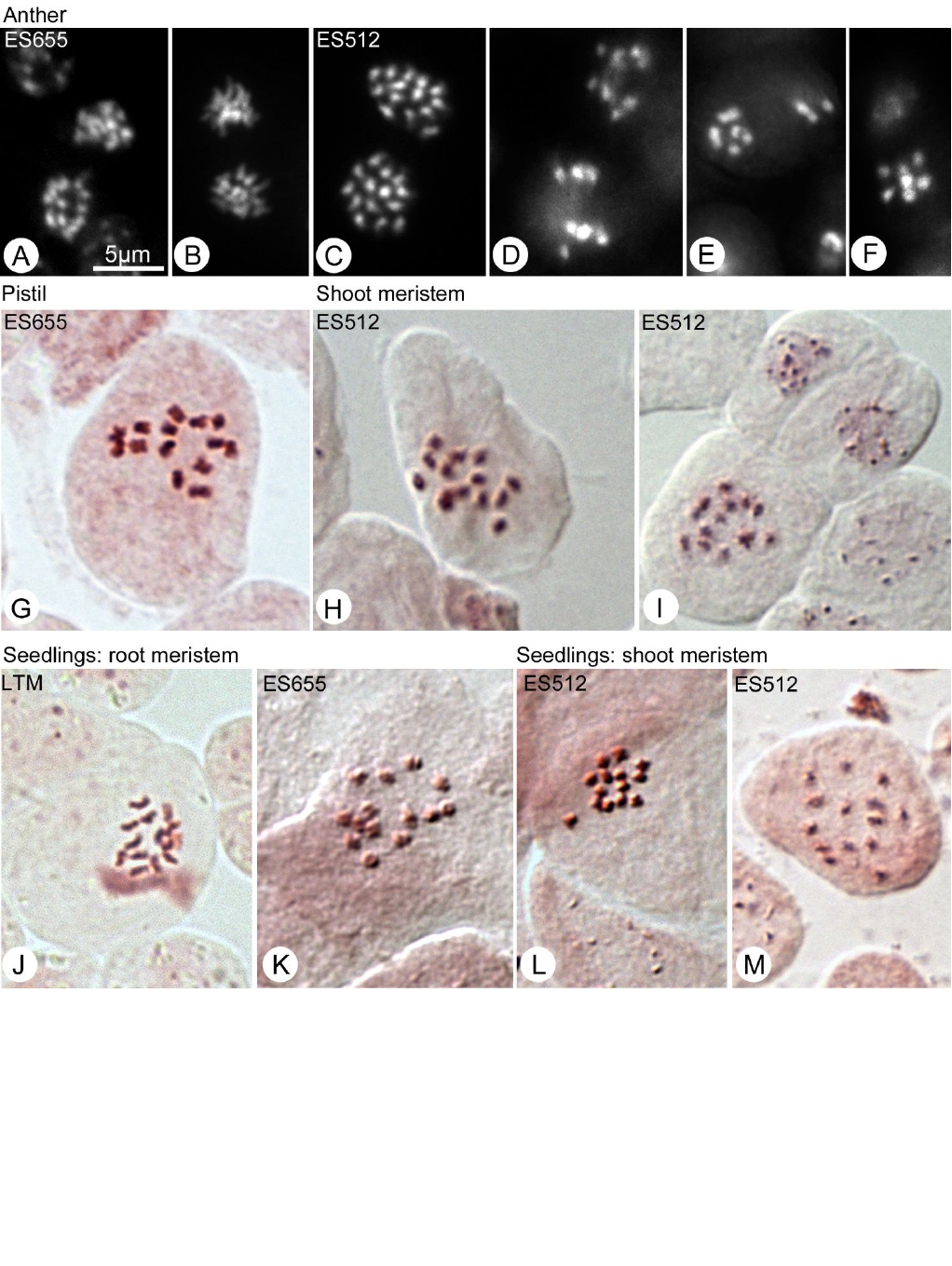

## Slide 5
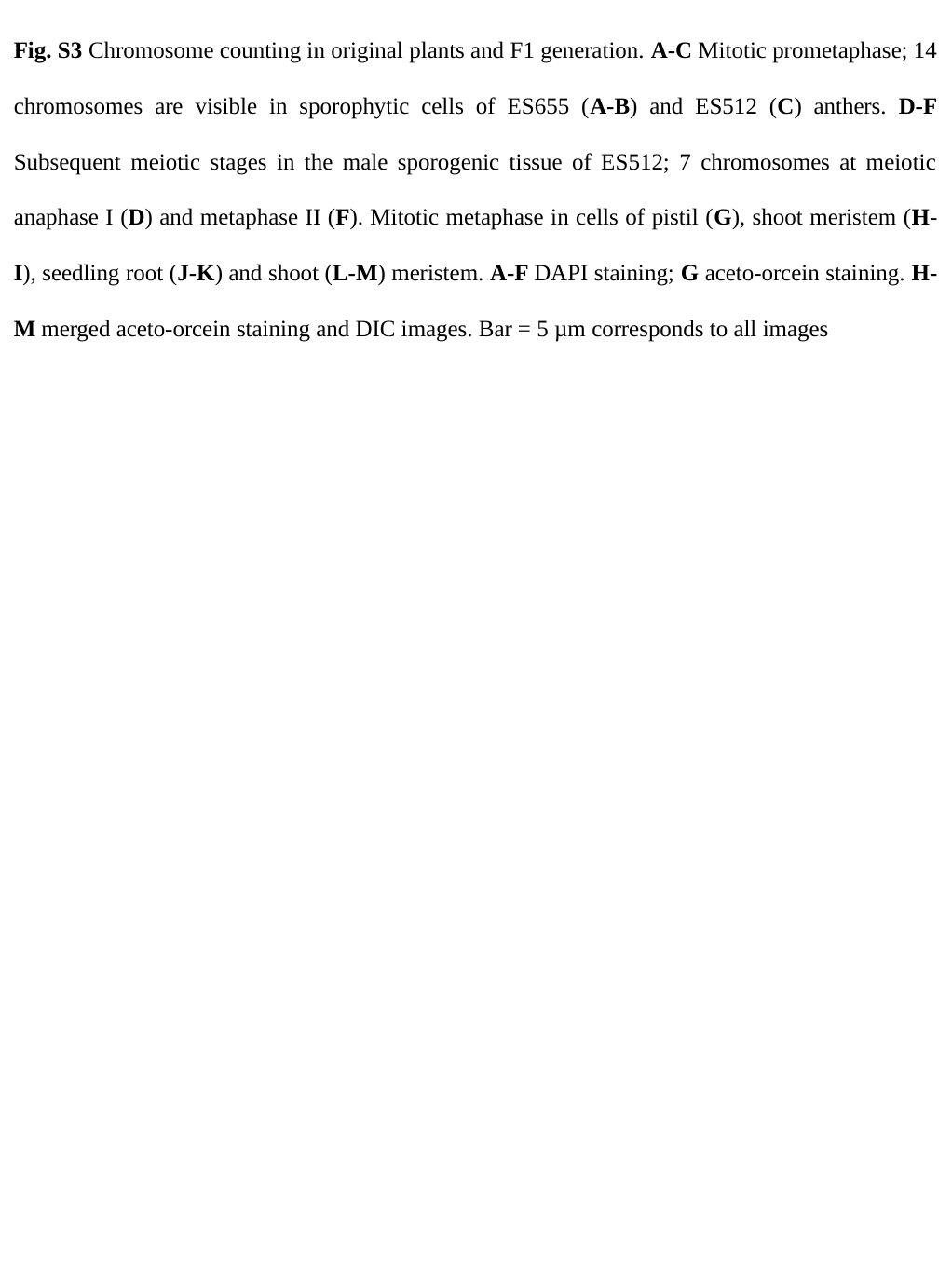

Fig. S3 Chromosome counting in original plants and F1 generation. A-C Mitotic prometaphase; 14 chromosomes are visible in sporophytic cells of ES655 (A-B) and ES512 (C) anthers. D-F Subsequent meiotic stages in the male sporogenic tissue of ES512; 7 chromosomes at meiotic anaphase I (D) and metaphase II (F). Mitotic metaphase in cells of pistil (G), shoot meristem (H-I), seedling root (J-K) and shoot (L-M) meristem. A-F DAPI staining; G aceto-orcein staining. H-M merged aceto-orcein staining and DIC images. Bar = 5 µm corresponds to all images

## Slide 6
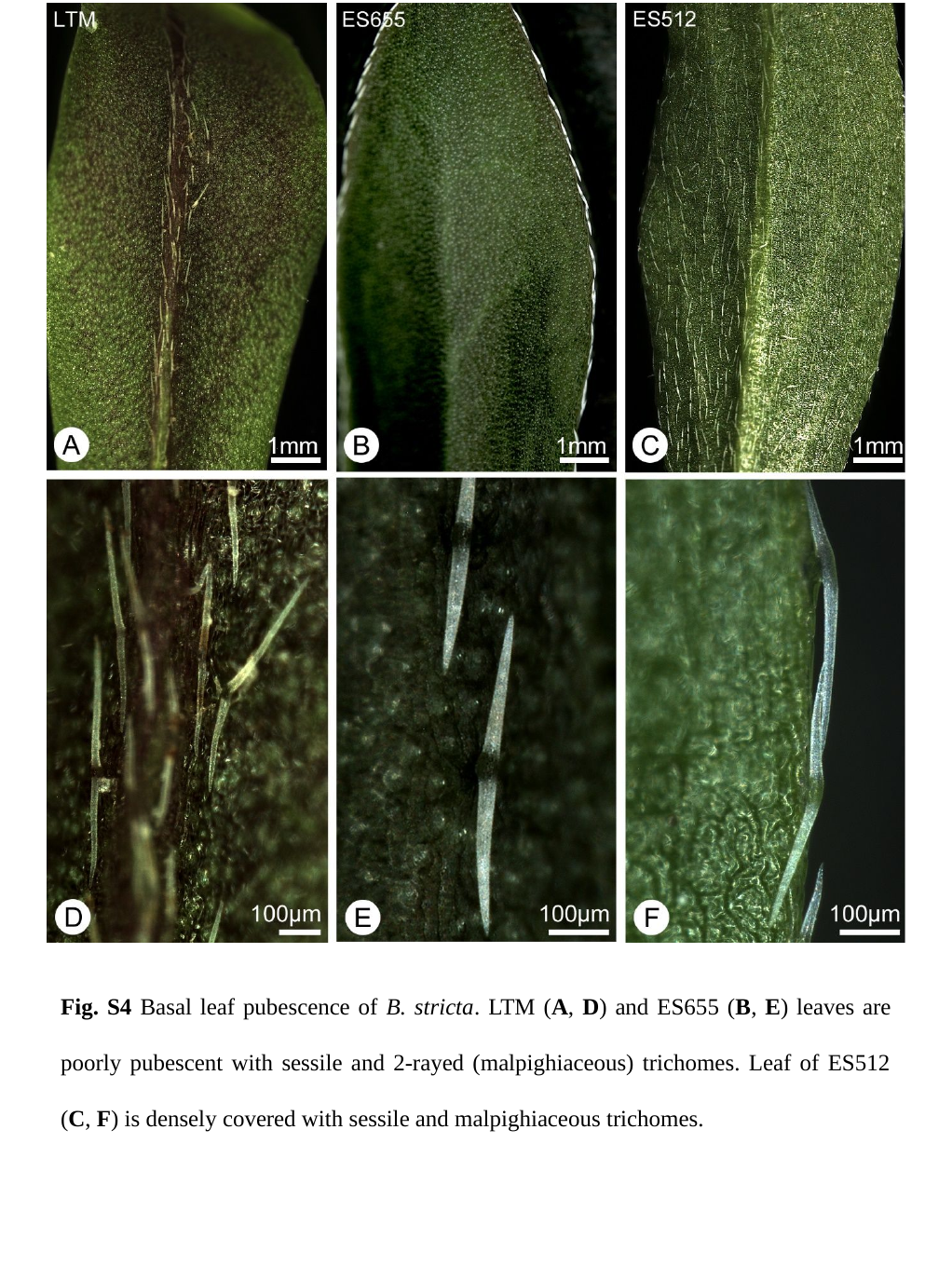

Fig. S4 Basal leaf pubescence of B. stricta. LTM (A, D) and ES655 (B, E) leaves are poorly pubescent with sessile and 2-rayed (malpighiaceous) trichomes. Leaf of ES512 (C, F) is densely covered with sessile and malpighiaceous trichomes.

## Slide 7
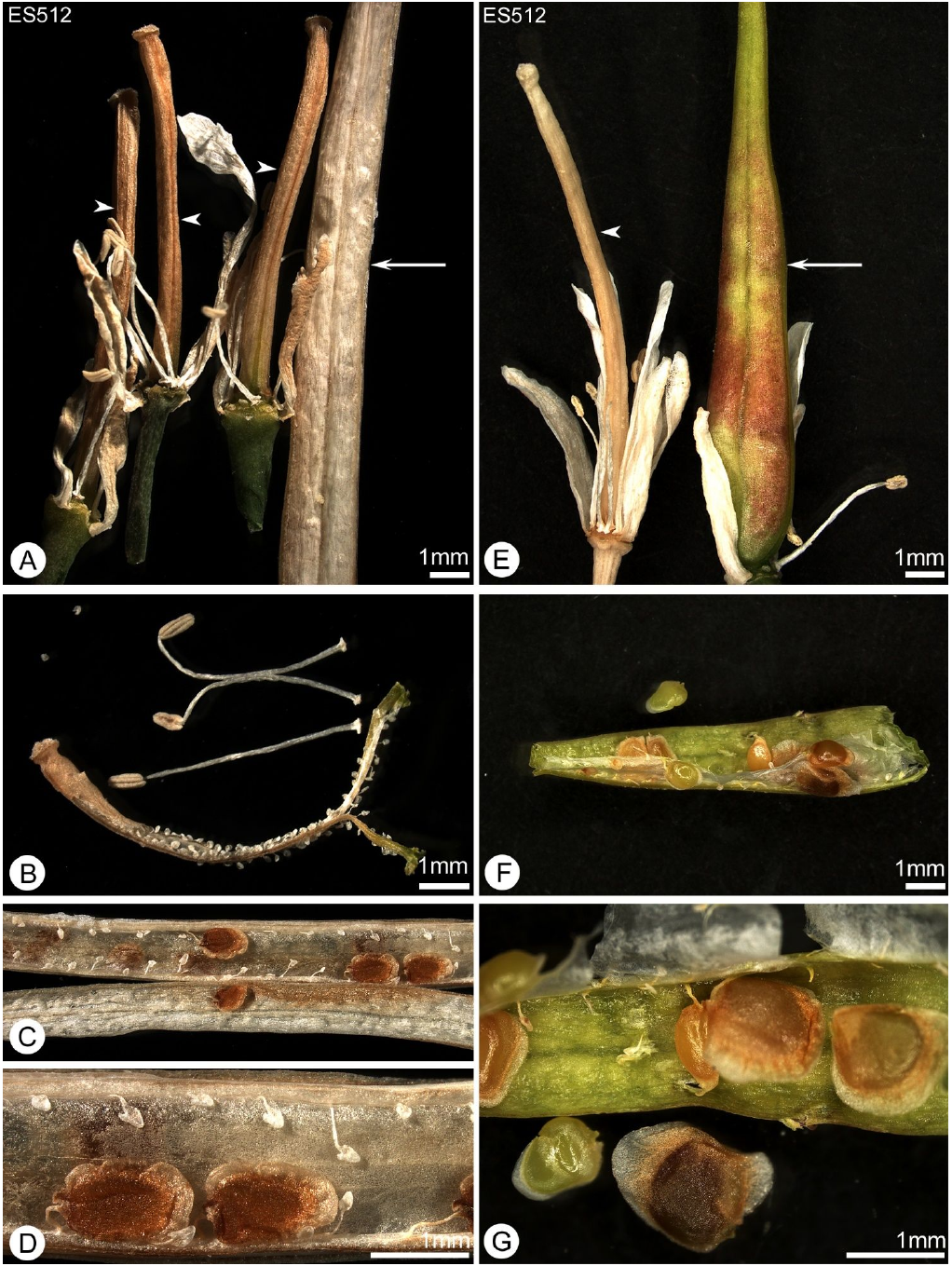

## Slide 8
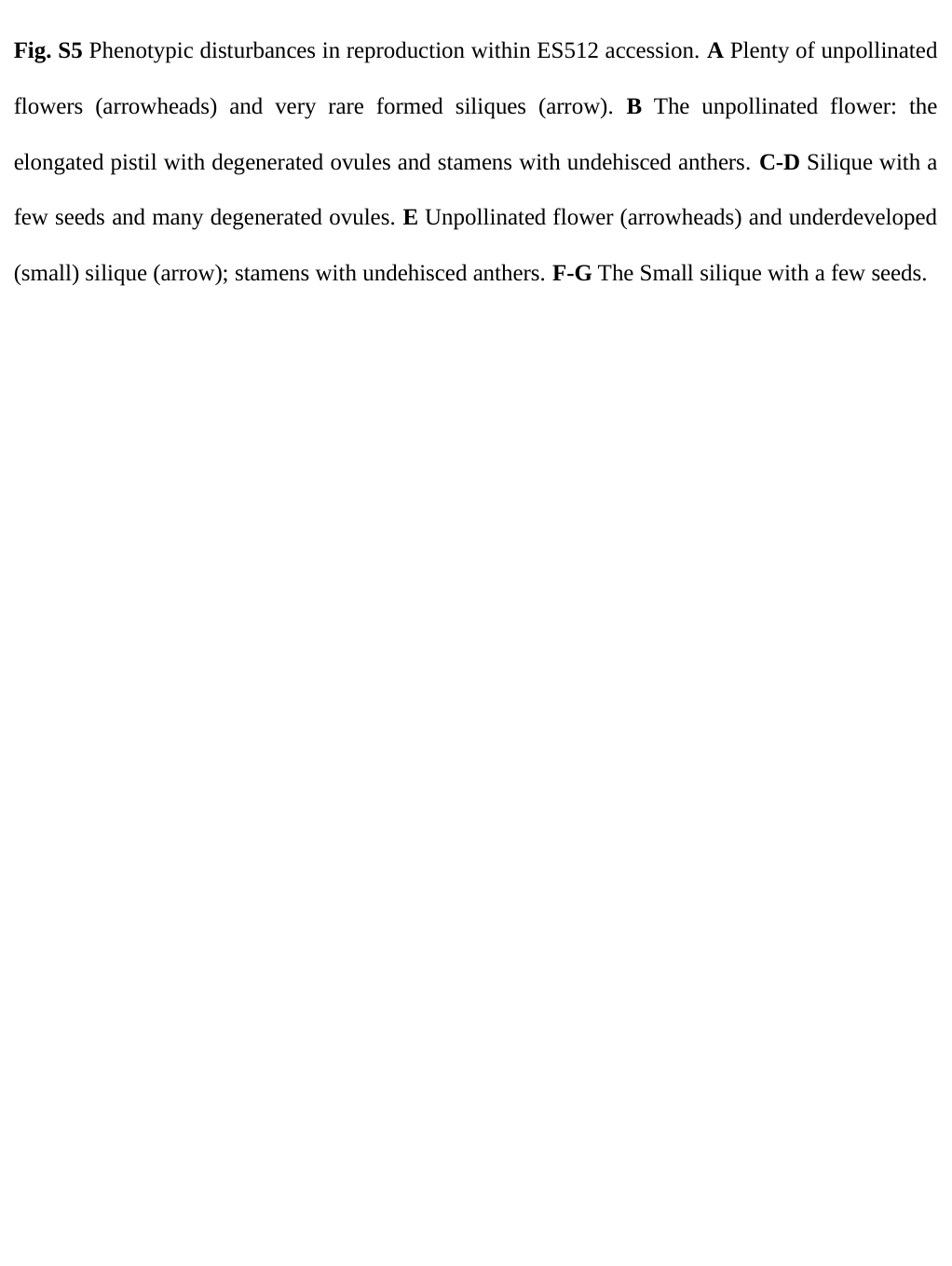

Fig. S5 Phenotypic disturbances in reproduction within ES512 accession. A Plenty of unpollinated flowers (arrowheads) and very rare formed siliques (arrow). B The unpollinated flower: the elongated pistil with degenerated ovules and stamens with undehisced anthers. C-D Silique with a few seeds and many degenerated ovules. E Unpollinated flower (arrowheads) and underdeveloped (small) silique (arrow); stamens with undehisced anthers. F-G The Small silique with a few seeds.

## Slide 9
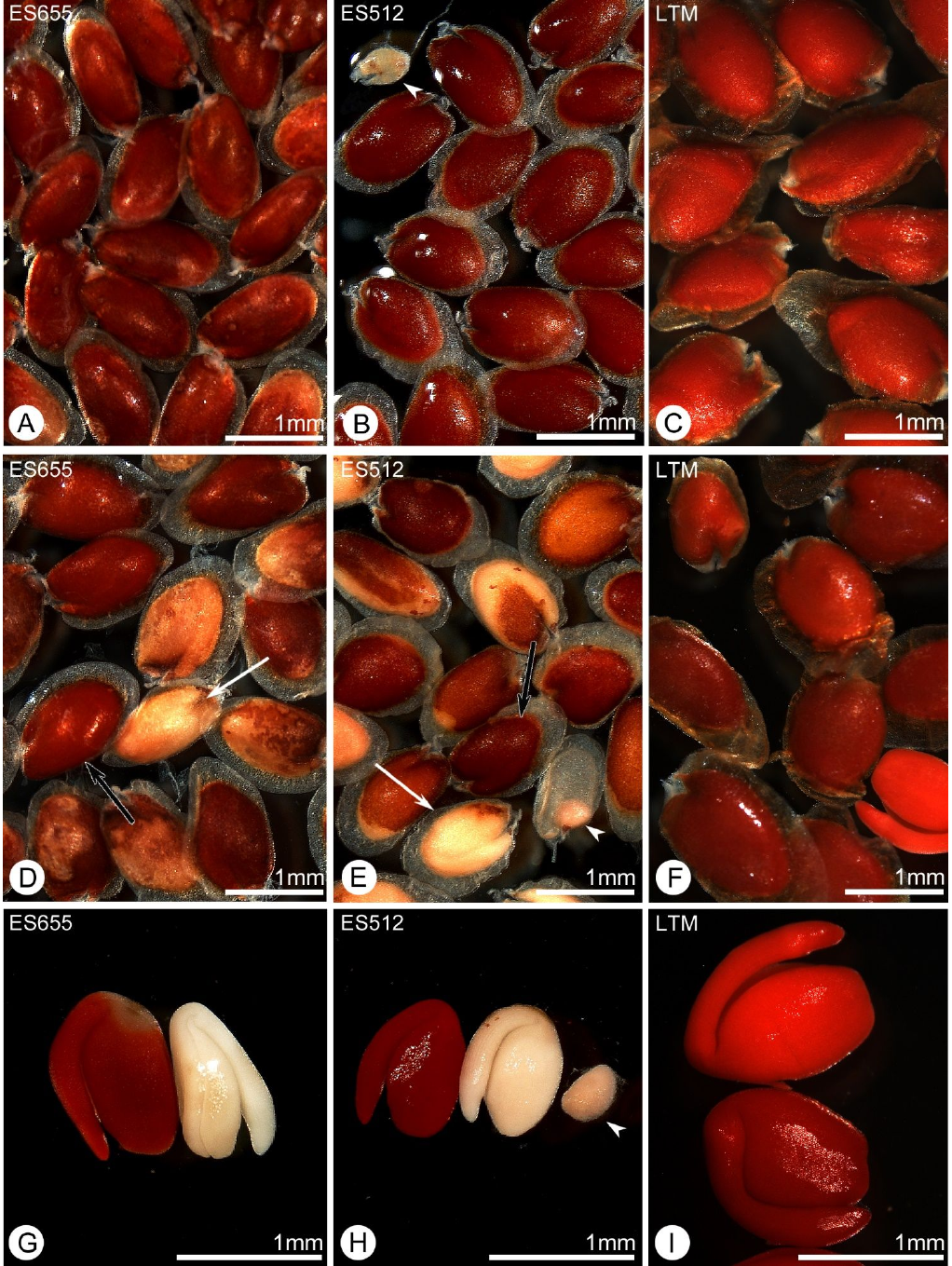

## Slide 10
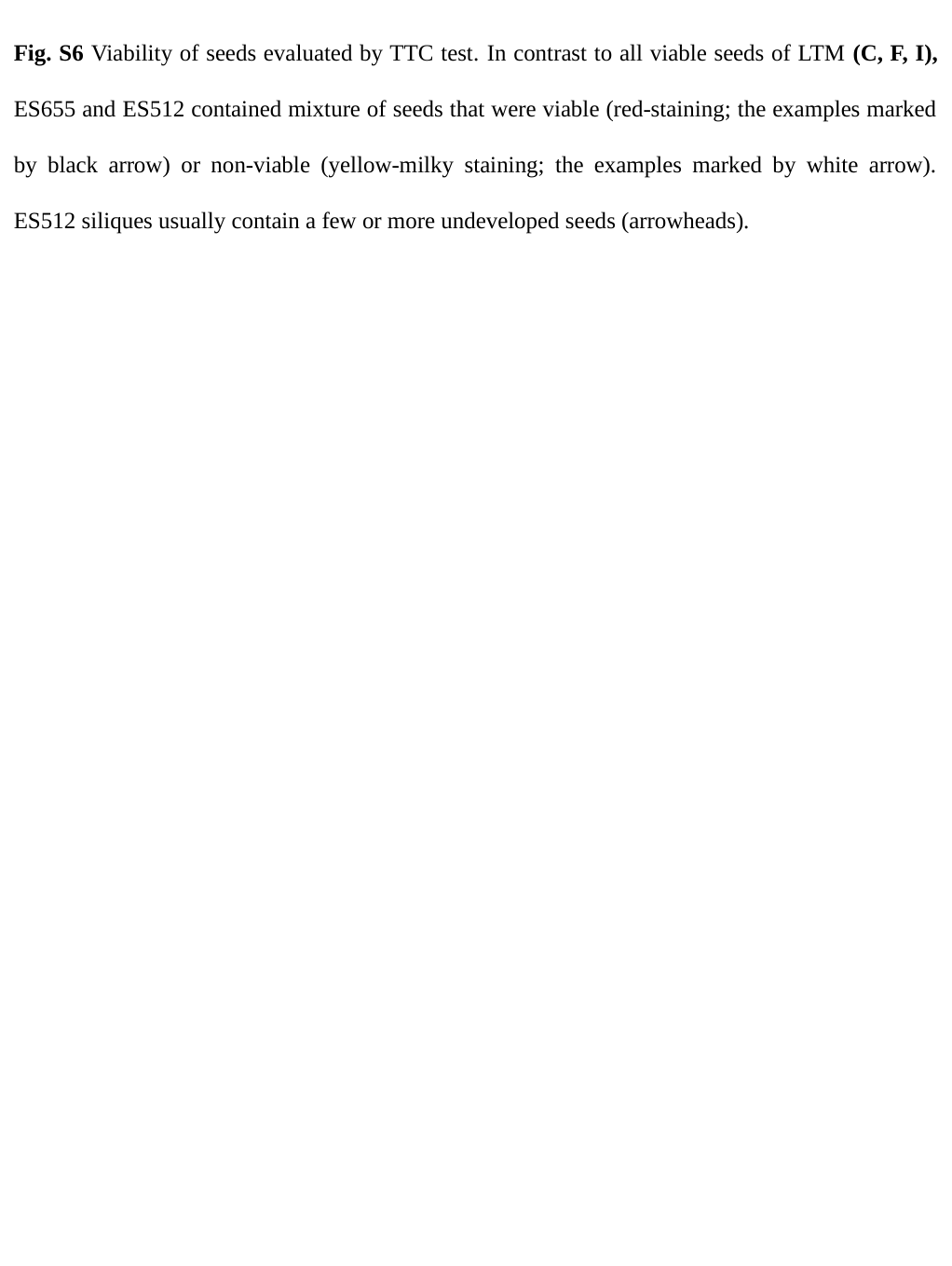

Fig. S6 Viability of seeds evaluated by TTC test. In contrast to all viable seeds of LTM (C, F, I), ES655 and ES512 contained mixture of seeds that were viable (red-staining; the examples marked by black arrow) or non-viable (yellow-milky staining; the examples marked by white arrow). ES512 siliques usually contain a few or more undeveloped seeds (arrowheads).

## Slide 11
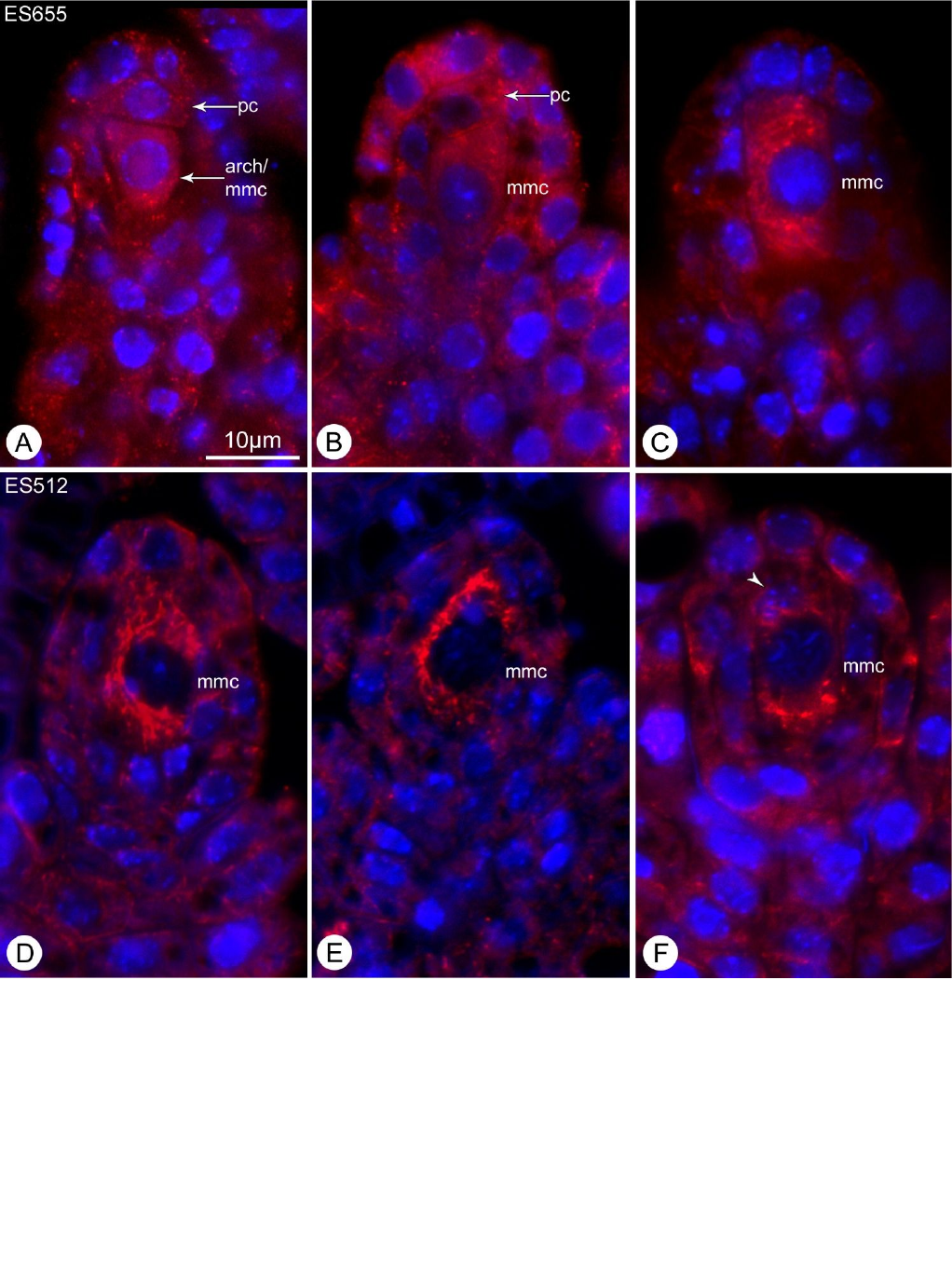

## Slide 12
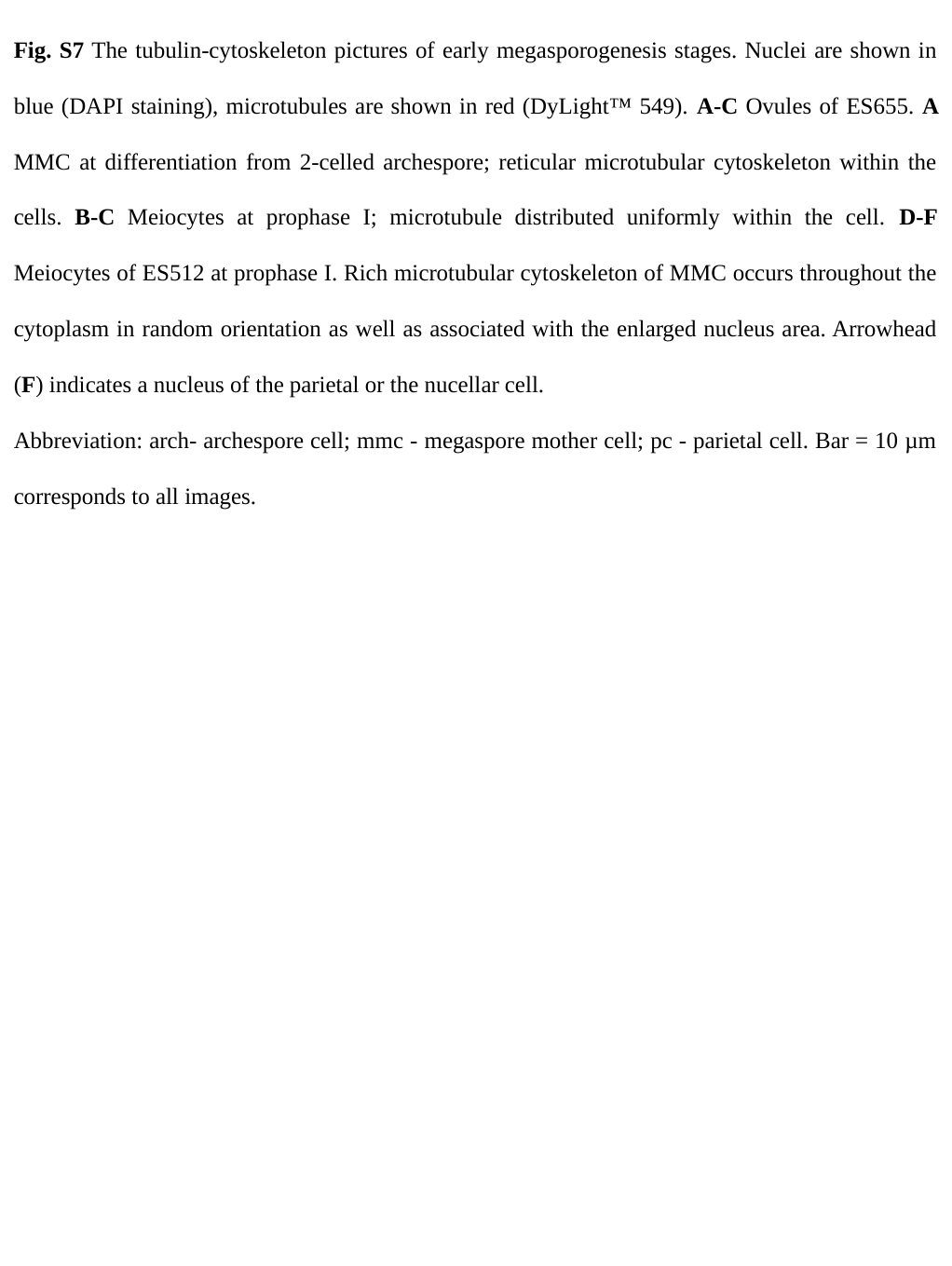

Fig. S7 The tubulin-cytoskeleton pictures of early megasporogenesis stages. Nuclei are shown in blue (DAPI staining), microtubules are shown in red (DyLight™ 549). A-C Ovules of ES655. A MMC at differentiation from 2-celled archespore; reticular microtubular cytoskeleton within the cells. B-C Meiocytes at prophase I; microtubule distributed uniformly within the cell. D-F Meiocytes of ES512 at prophase I. Rich microtubular cytoskeleton of MMC occurs throughout the cytoplasm in random orientation as well as associated with the enlarged nucleus area. Arrowhead (F) indicates a nucleus of the parietal or the nucellar cell.
Abbreviation: arch- archespore cell; mmc - megaspore mother cell; pc - parietal cell. Bar = 10 µm corresponds to all images.

## Slide 13
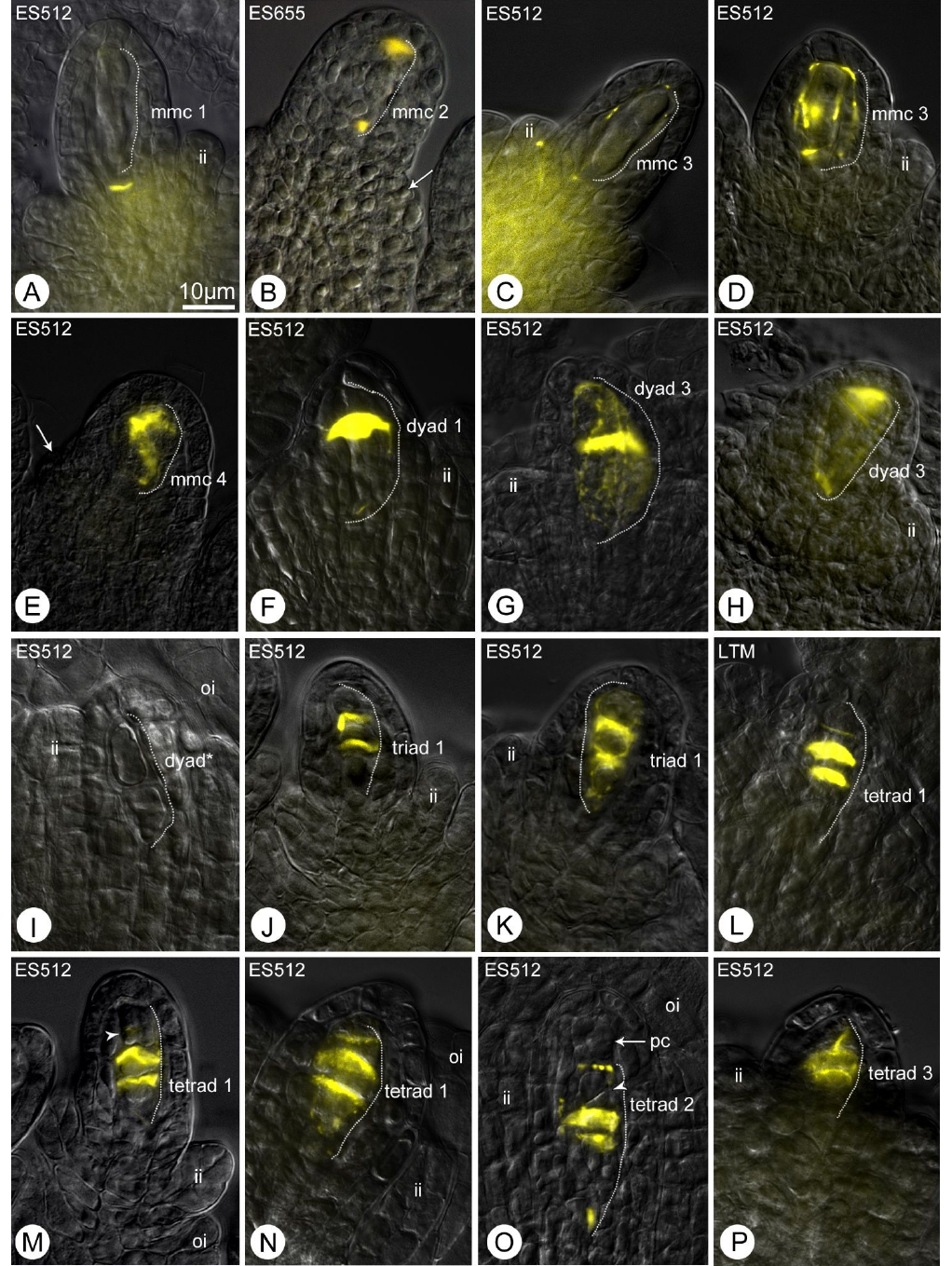

## Slide 14
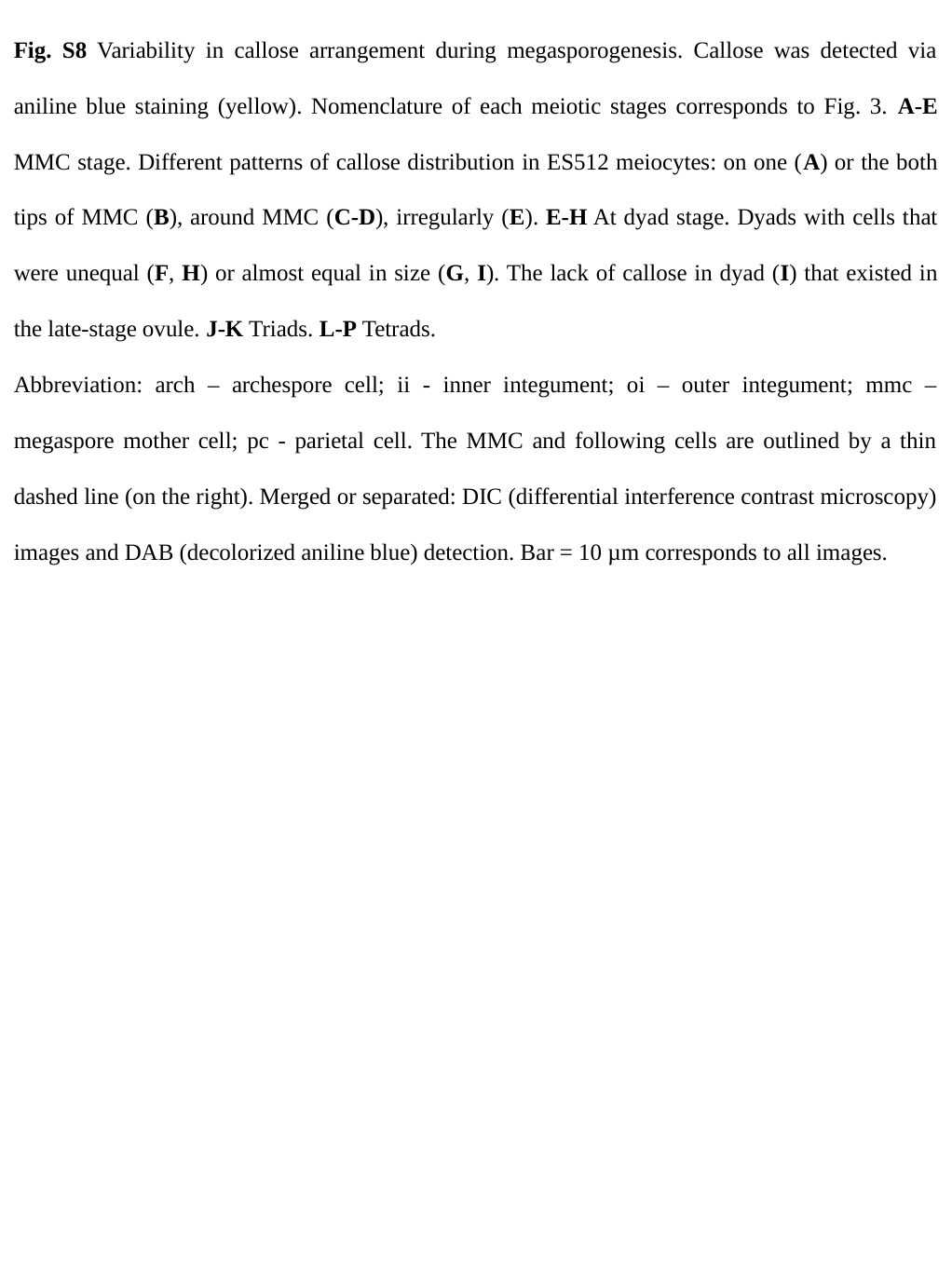

Fig. S8 Variability in callose arrangement during megasporogenesis. Callose was detected via aniline blue staining (yellow). Nomenclature of each meiotic stages corresponds to Fig. 3. A-E MMC stage. Different patterns of callose distribution in ES512 meiocytes: on one (A) or the both tips of MMC (B), around MMC (C-D), irregularly (E). E-H At dyad stage. Dyads with cells that were unequal (F, H) or almost equal in size (G, I). The lack of callose in dyad (I) that existed in the late-stage ovule. J-K Triads. L-P Tetrads.
Abbreviation: arch – archespore cell; ii - inner integument; oi – outer integument; mmc – megaspore mother cell; pc - parietal cell. The MMC and following cells are outlined by a thin dashed line (on the right). Merged or separated: DIC (differential interference contrast microscopy) images and DAB (decolorized aniline blue) detection. Bar = 10 µm corresponds to all images.

## Slide 15
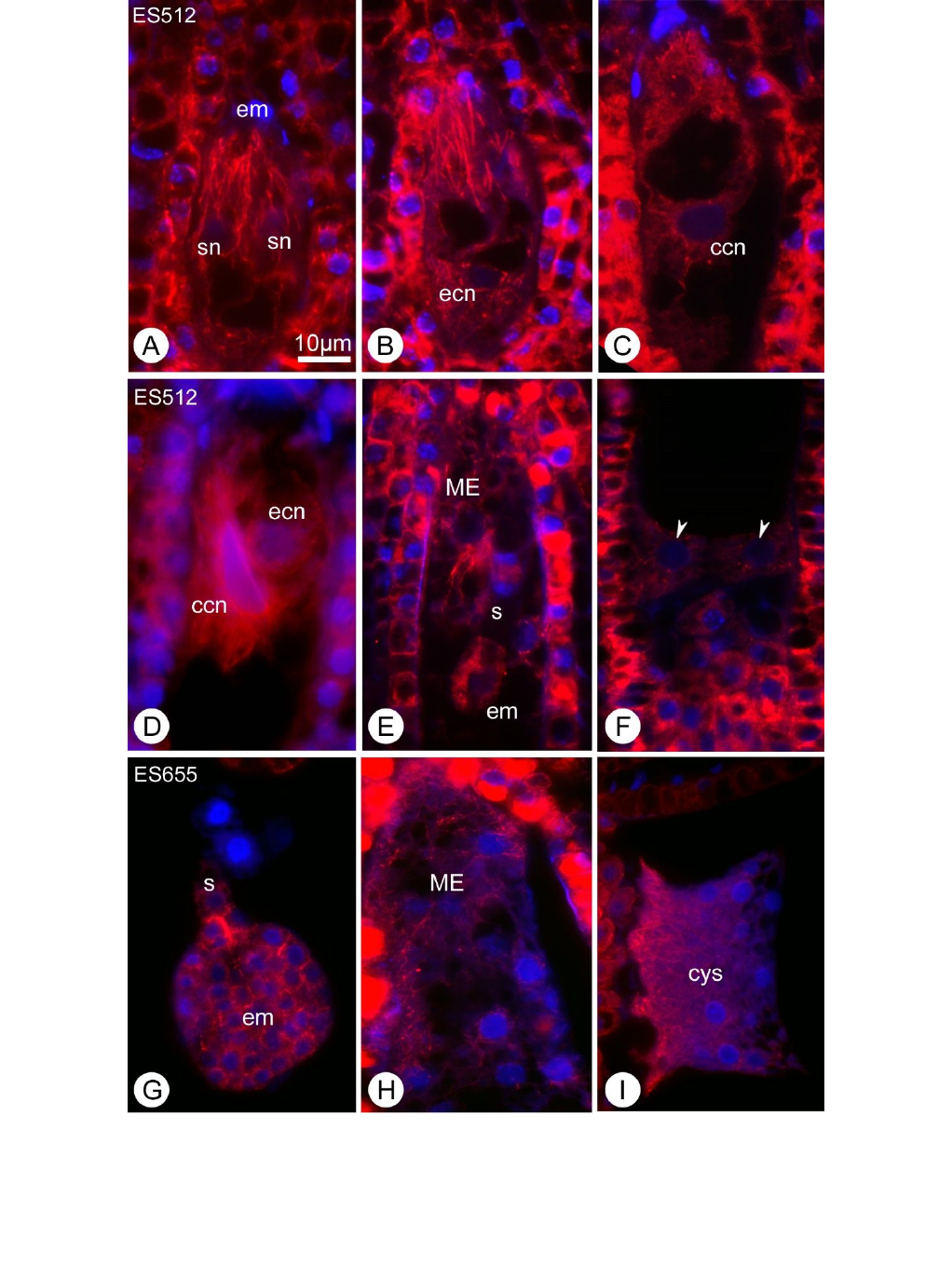

## Slide 16
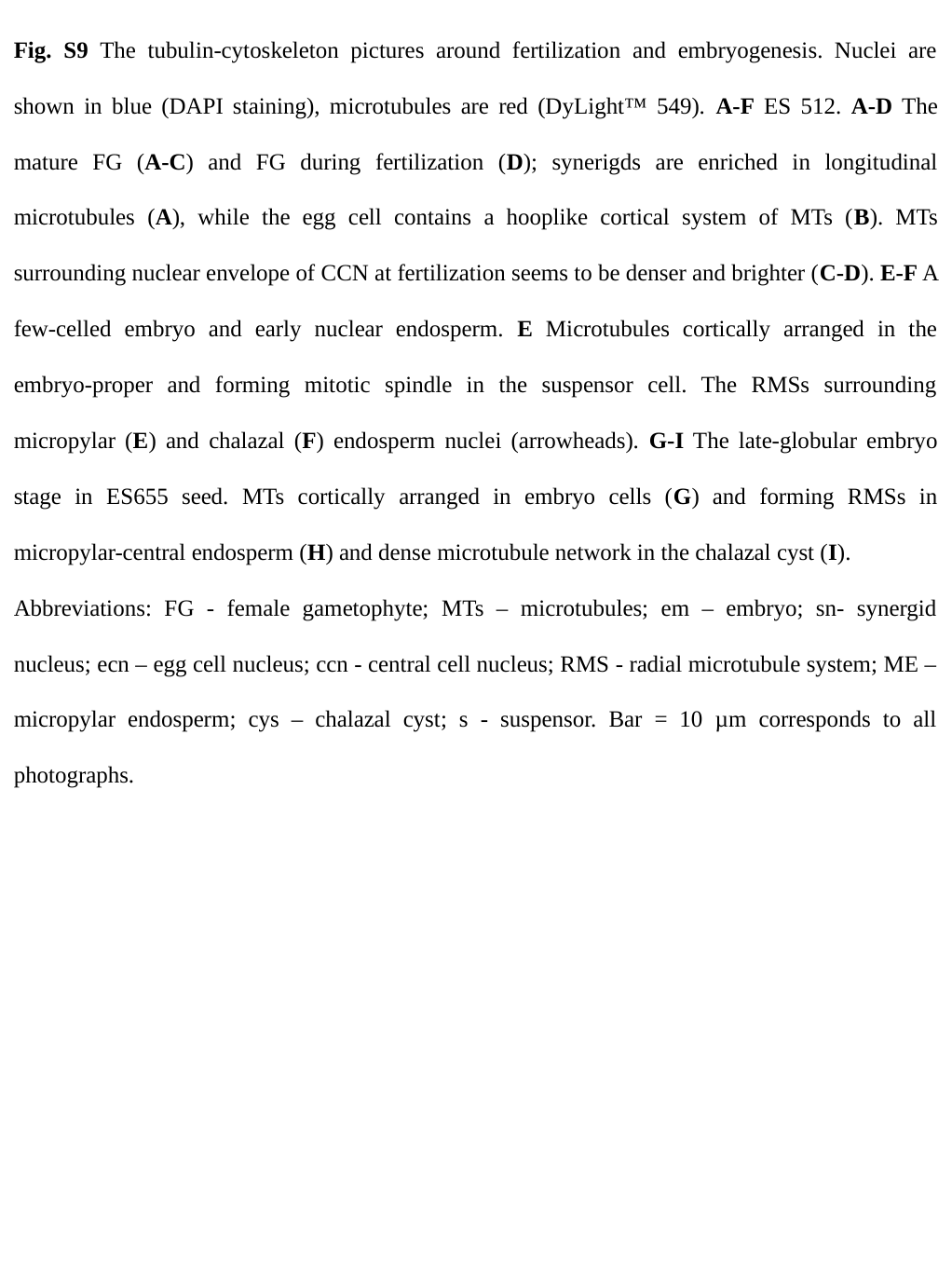

Fig. S9 The tubulin-cytoskeleton pictures around fertilization and embryogenesis. Nuclei are shown in blue (DAPI staining), microtubules are red (DyLight™ 549). A-F ES 512. A-D The mature FG (A-C) and FG during fertilization (D); synerigds are enriched in longitudinal microtubules (A), while the egg cell contains a hooplike cortical system of MTs (B). MTs surrounding nuclear envelope of CCN at fertilization seems to be denser and brighter (C-D). E-F A few-celled embryo and early nuclear endosperm. E Microtubules cortically arranged in the embryo-proper and forming mitotic spindle in the suspensor cell. The RMSs surrounding micropylar (E) and chalazal (F) endosperm nuclei (arrowheads). G-I The late-globular embryo stage in ES655 seed. MTs cortically arranged in embryo cells (G) and forming RMSs in micropylar-central endosperm (H) and dense microtubule network in the chalazal cyst (I).
Abbreviations: FG - female gametophyte; MTs – microtubules; em – embryo; sn- synergid nucleus; ecn – egg cell nucleus; ccn - central cell nucleus; RMS - radial microtubule system; ME – micropylar endosperm; cys – chalazal cyst; s - suspensor. Bar = 10 µm corresponds to all photographs.

## Slide 17
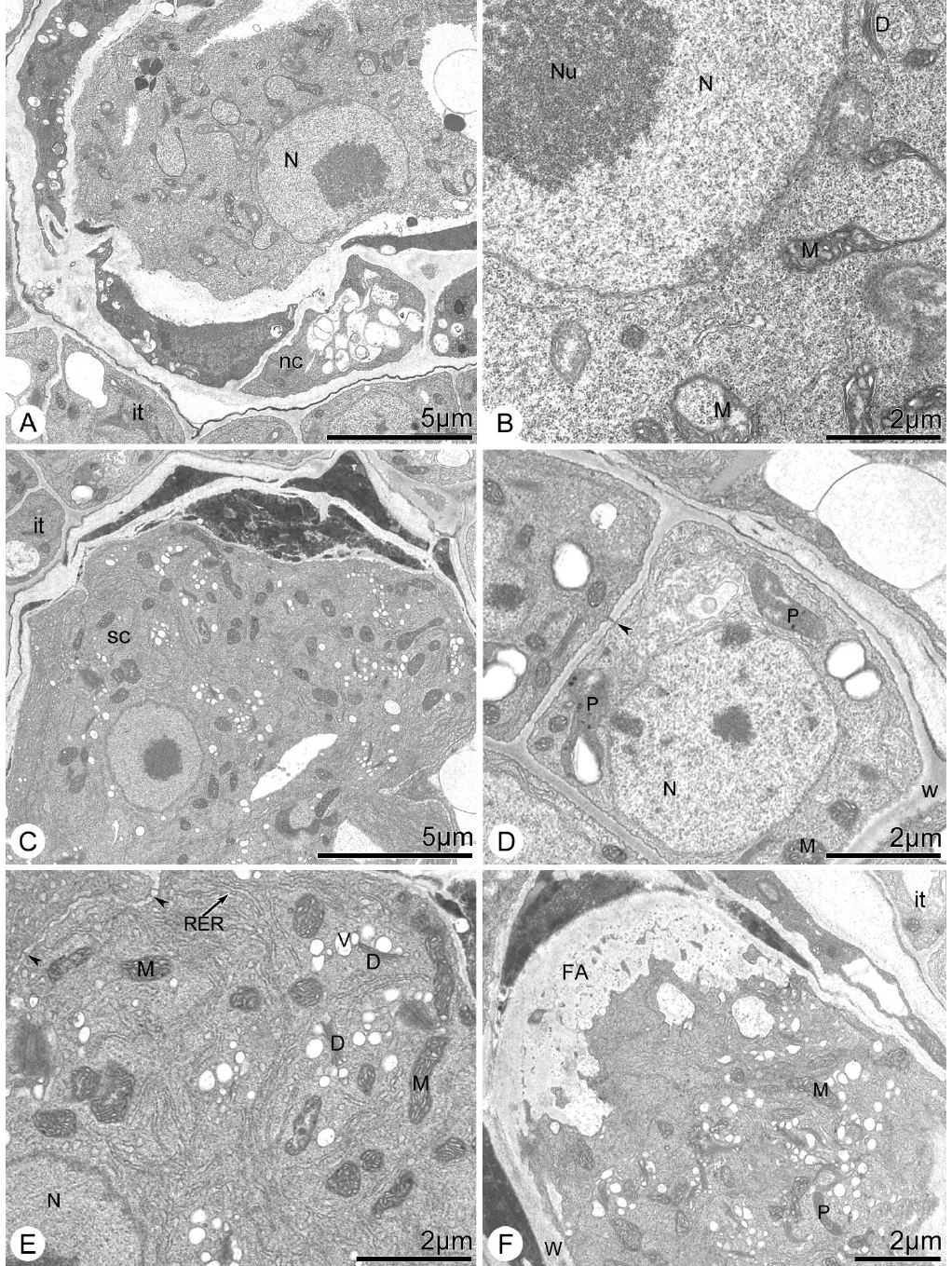

## Slide 18
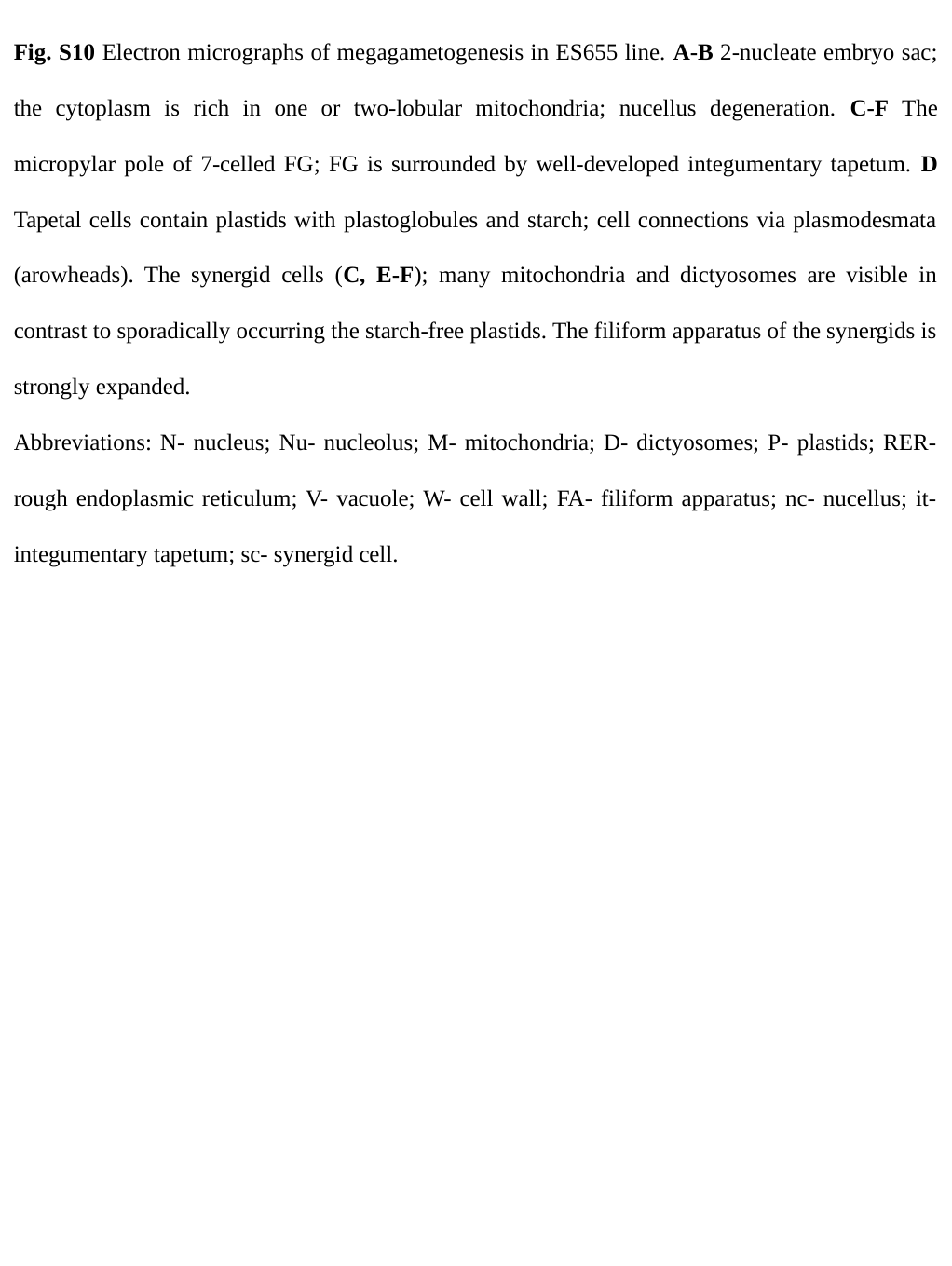

Fig. S10 Electron micrographs of megagametogenesis in ES655 line. A-B 2-nucleate embryo sac; the cytoplasm is rich in one or two-lobular mitochondria; nucellus degeneration. C-F The micropylar pole of 7-celled FG; FG is surrounded by well-developed integumentary tapetum. D Tapetal cells contain plastids with plastoglobules and starch; cell connections via plasmodesmata (arowheads). The synergid cells (C, E-F); many mitochondria and dictyosomes are visible in contrast to sporadically occurring the starch-free plastids. The filiform apparatus of the synergids is strongly expanded.
Abbreviations: N- nucleus; Nu- nucleolus; M- mitochondria; D- dictyosomes; P- plastids; RER- rough endoplasmic reticulum; V- vacuole; W- cell wall; FA- filiform apparatus; nc- nucellus; it- integumentary tapetum; sc- synergid cell.

## Slide 19
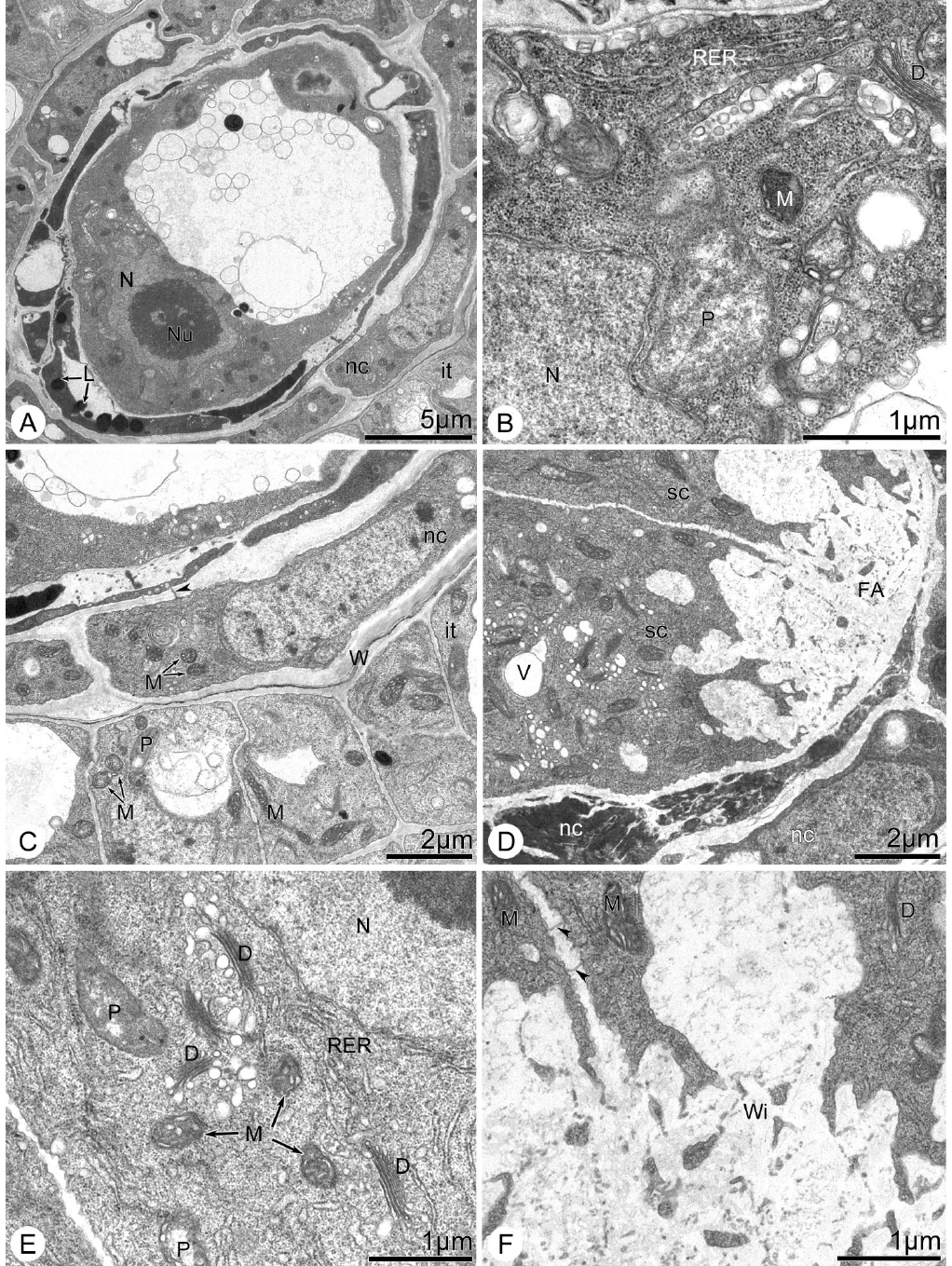

## Slide 20
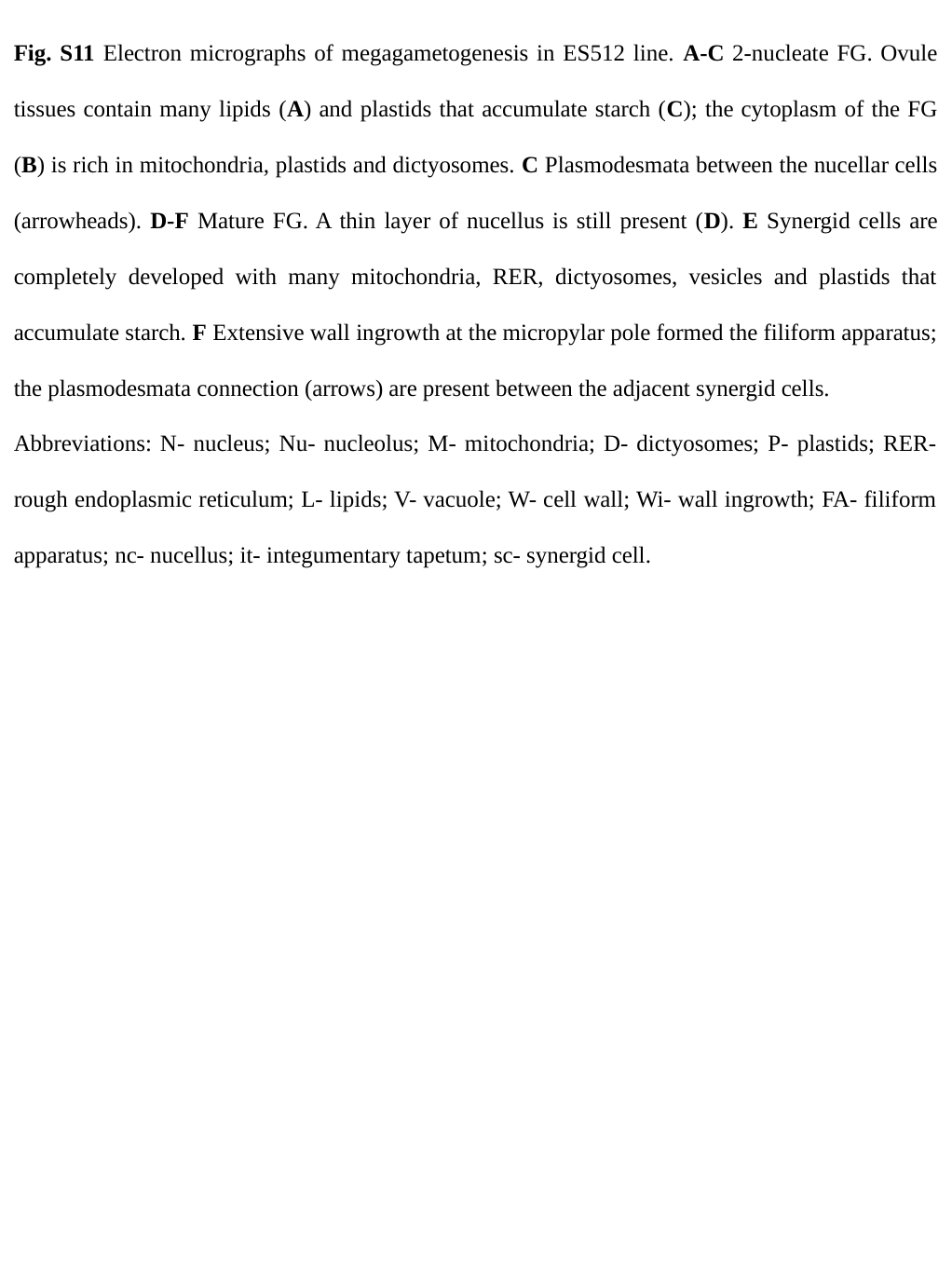

Fig. S11 Electron micrographs of megagametogenesis in ES512 line. A-C 2-nucleate FG. Ovule tissues contain many lipids (A) and plastids that accumulate starch (C); the cytoplasm of the FG (B) is rich in mitochondria, plastids and dictyosomes. C Plasmodesmata between the nucellar cells (arrowheads). D-F Mature FG. A thin layer of nucellus is still present (D). E Synergid cells are completely developed with many mitochondria, RER, dictyosomes, vesicles and plastids that accumulate starch. F Extensive wall ingrowth at the micropylar pole formed the filiform apparatus; the plasmodesmata connection (arrows) are present between the adjacent synergid cells.
Abbreviations: N- nucleus; Nu- nucleolus; M- mitochondria; D- dictyosomes; P- plastids; RER- rough endoplasmic reticulum; L- lipids; V- vacuole; W- cell wall; Wi- wall ingrowth; FA- filiform apparatus; nc- nucellus; it- integumentary tapetum; sc- synergid cell.

## Slide 21
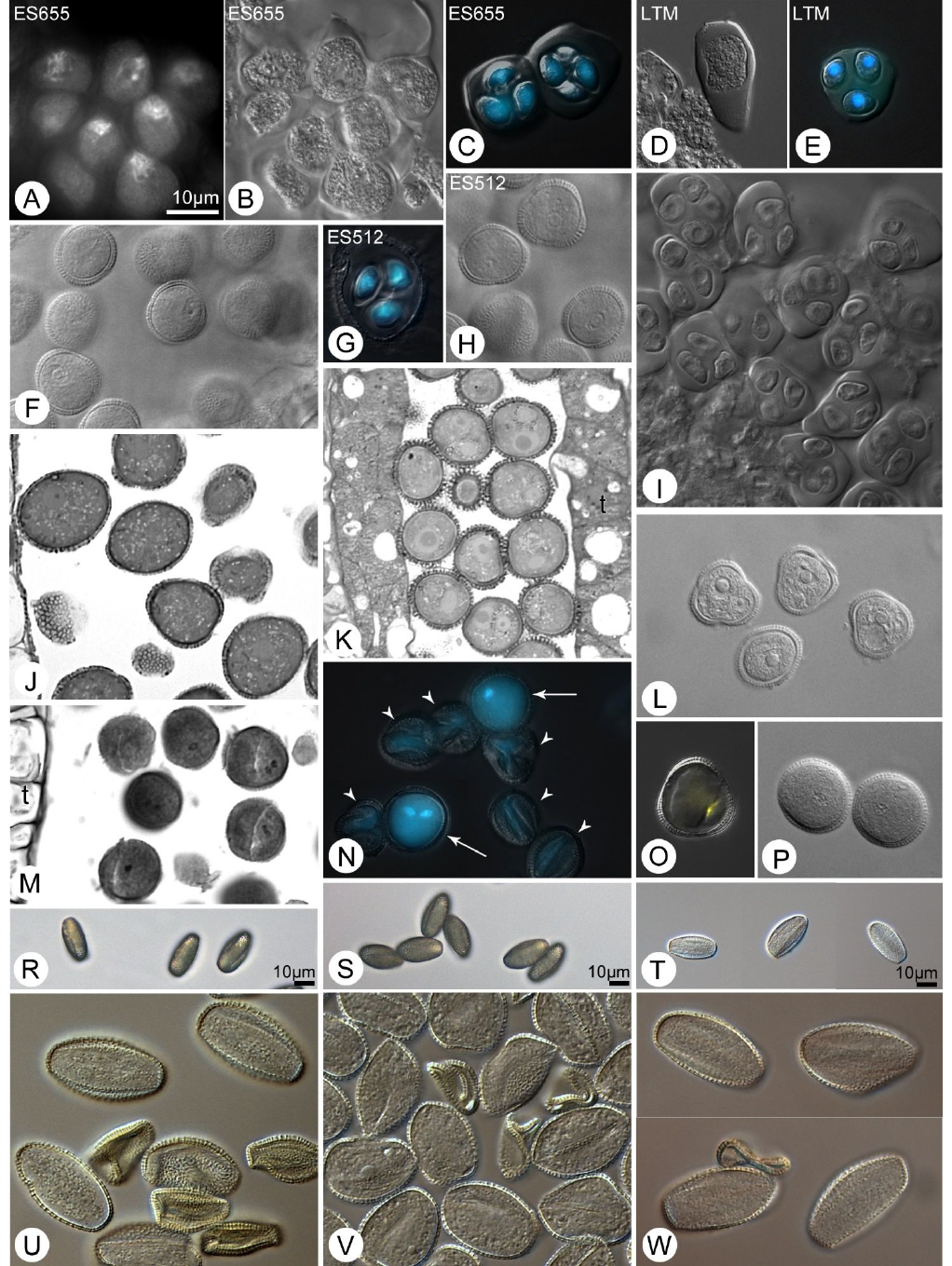

## Slide 22
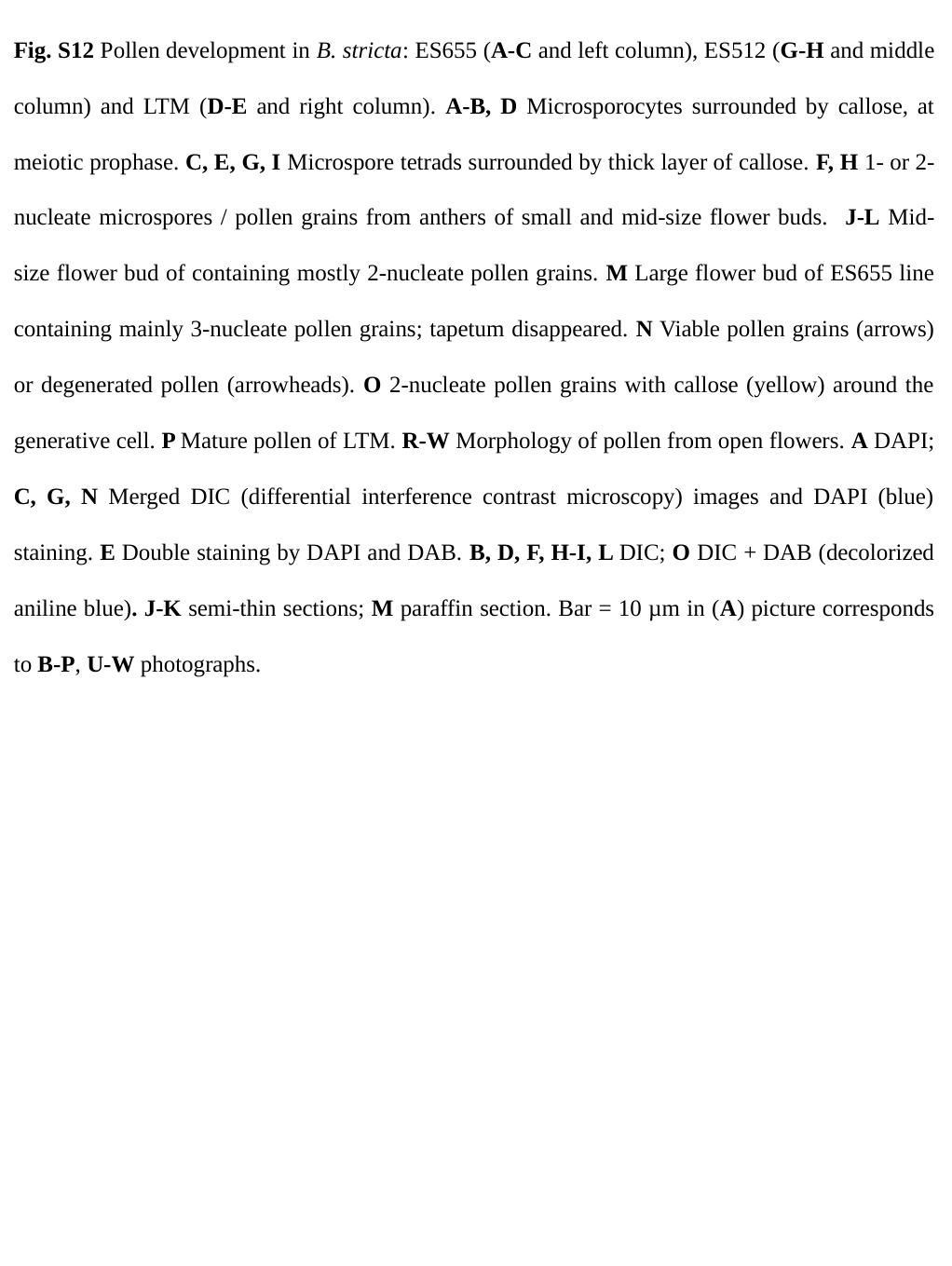

Fig. S12 Pollen development in B. stricta: ES655 (A-C and left column), ES512 (G-H and middle column) and LTM (D-E and right column). A-B, D Microsporocytes surrounded by callose, at meiotic prophase. C, E, G, I Microspore tetrads surrounded by thick layer of callose. F, H 1- or 2-nucleate microspores / pollen grains from anthers of small and mid-size flower buds. J-L Mid-size flower bud of containing mostly 2-nucleate pollen grains. M Large flower bud of ES655 line containing mainly 3-nucleate pollen grains; tapetum disappeared. N Viable pollen grains (arrows) or degenerated pollen (arrowheads). O 2-nucleate pollen grains with callose (yellow) around the generative cell. P Mature pollen of LTM. R-W Morphology of pollen from open flowers. A DAPI; C, G, N Merged DIC (differential interference contrast microscopy) images and DAPI (blue) staining. E Double staining by DAPI and DAB. B, D, F, H-I, L DIC; O DIC + DAB (decolorized aniline blue). J-K semi-thin sections; M paraffin section. Bar = 10 µm in (A) picture corresponds to B-P, U-W photographs.

## Slide 23
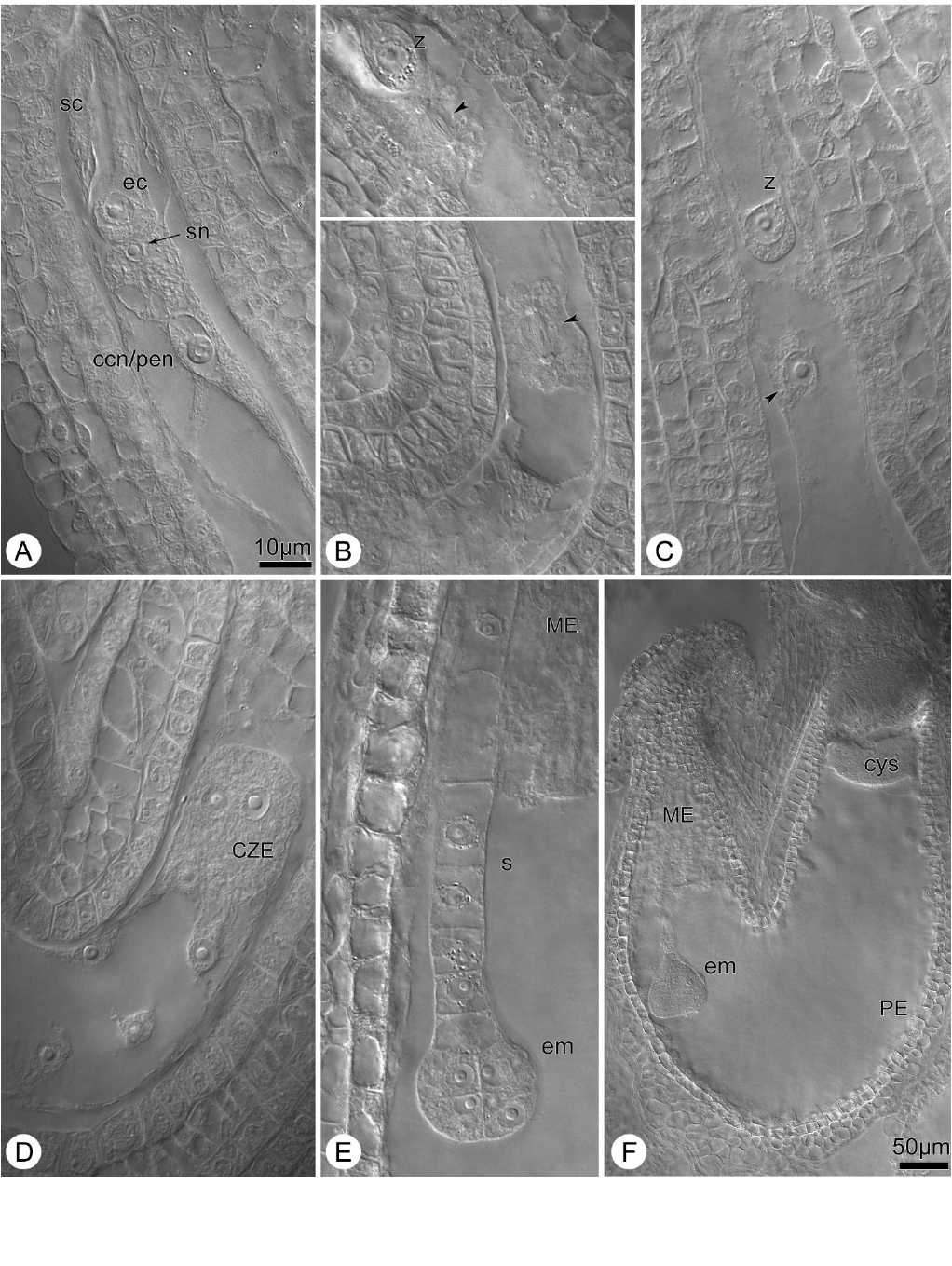

## Slide 24
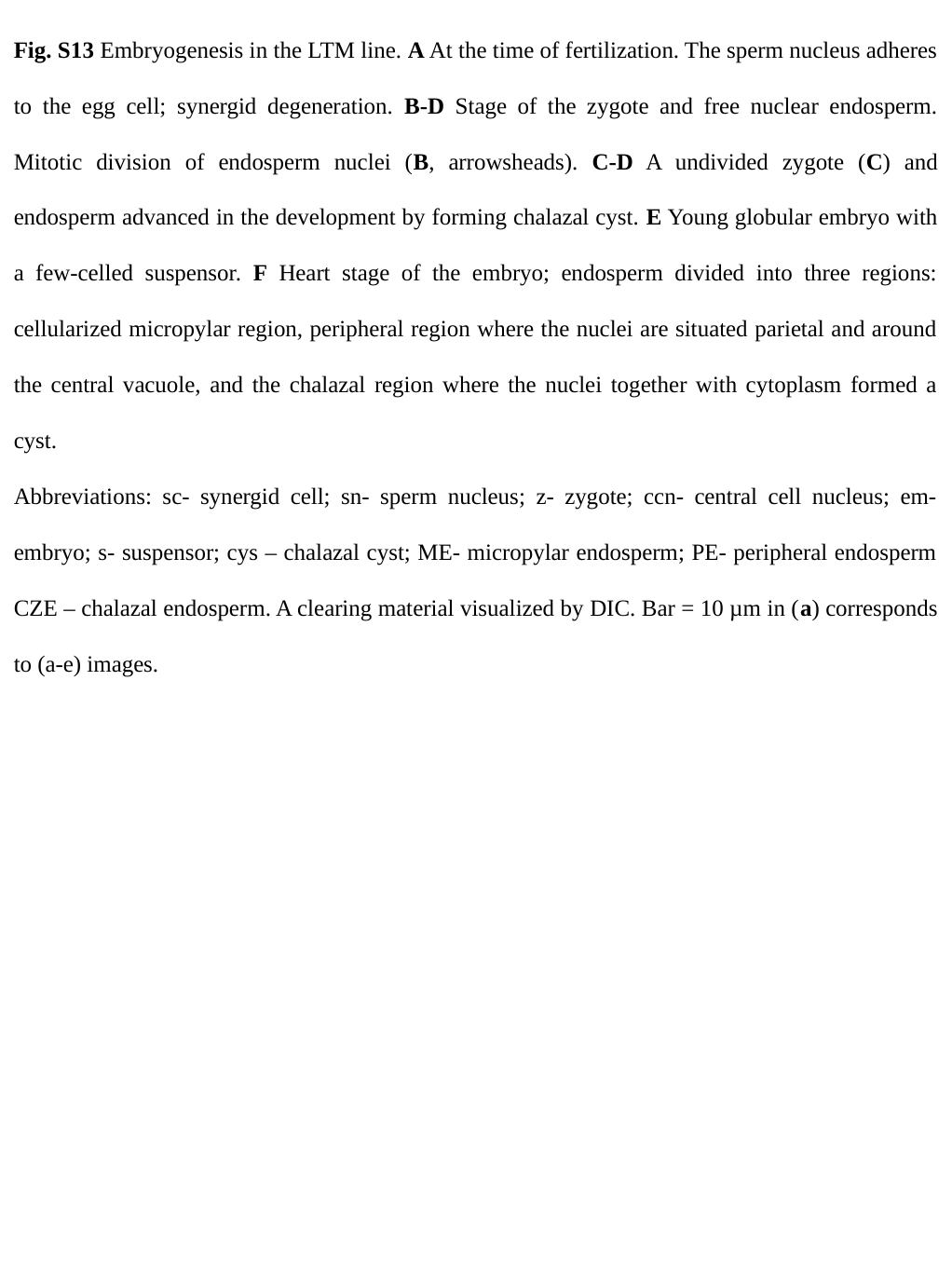

Fig. S13 Embryogenesis in the LTM line. A At the time of fertilization. The sperm nucleus adheres to the egg cell; synergid degeneration. B-D Stage of the zygote and free nuclear endosperm. Mitotic division of endosperm nuclei (B, arrowsheads). C-D A undivided zygote (C) and endosperm advanced in the development by forming chalazal cyst. E Young globular embryo with a few-celled suspensor. F Heart stage of the embryo; endosperm divided into three regions: cellularized micropylar region, peripheral region where the nuclei are situated parietal and around the central vacuole, and the chalazal region where the nuclei together with cytoplasm formed a cyst.
Abbreviations: sc- synergid cell; sn- sperm nucleus; z- zygote; ccn- central cell nucleus; em- embryo; s- suspensor; cys – chalazal cyst; ME- micropylar endosperm; PE- peripheral endosperm CZE – chalazal endosperm. A clearing material visualized by DIC. Bar = 10 µm in (a) corresponds to (a-e) images.

## Slide 25
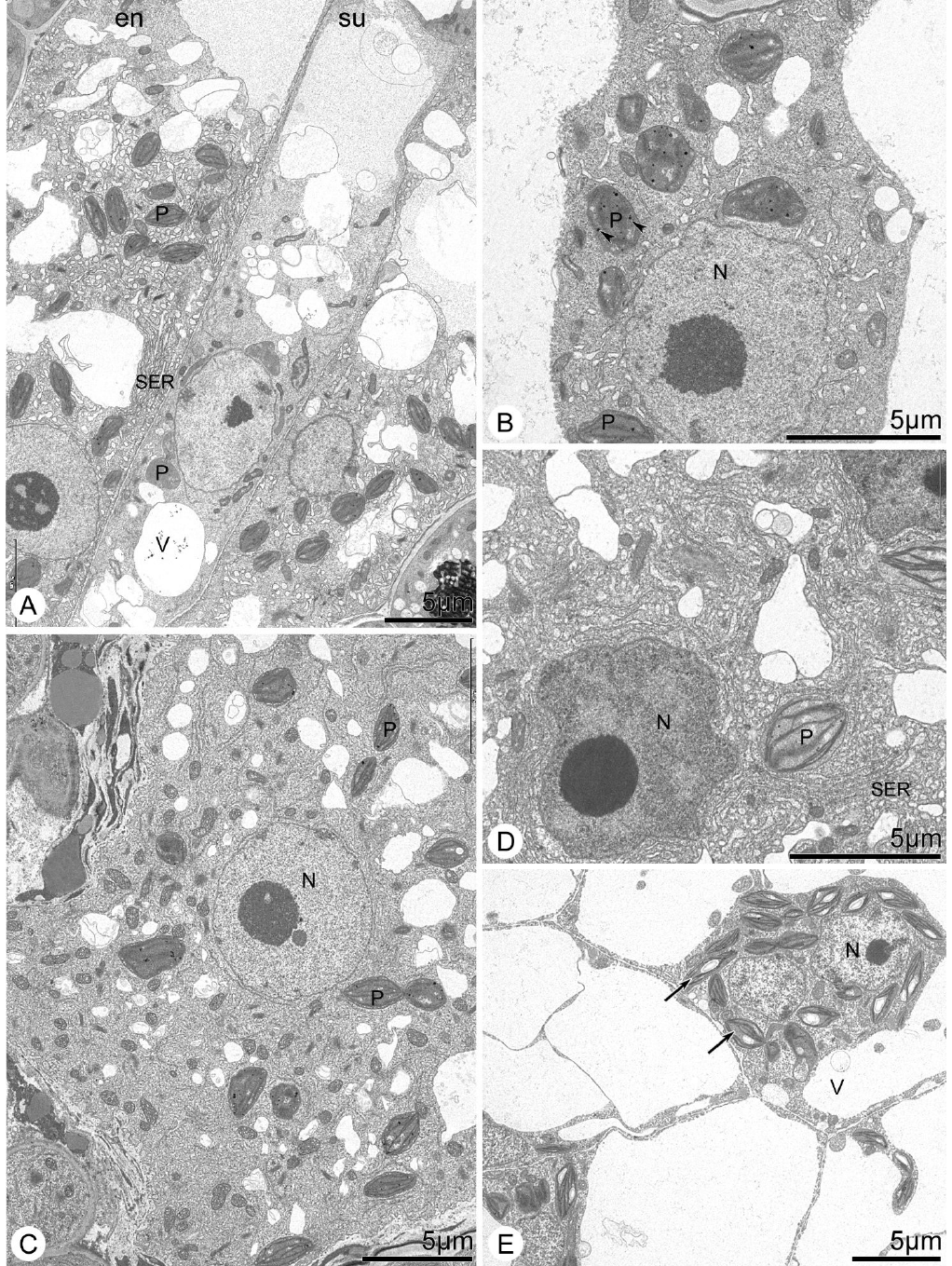

## Slide 26
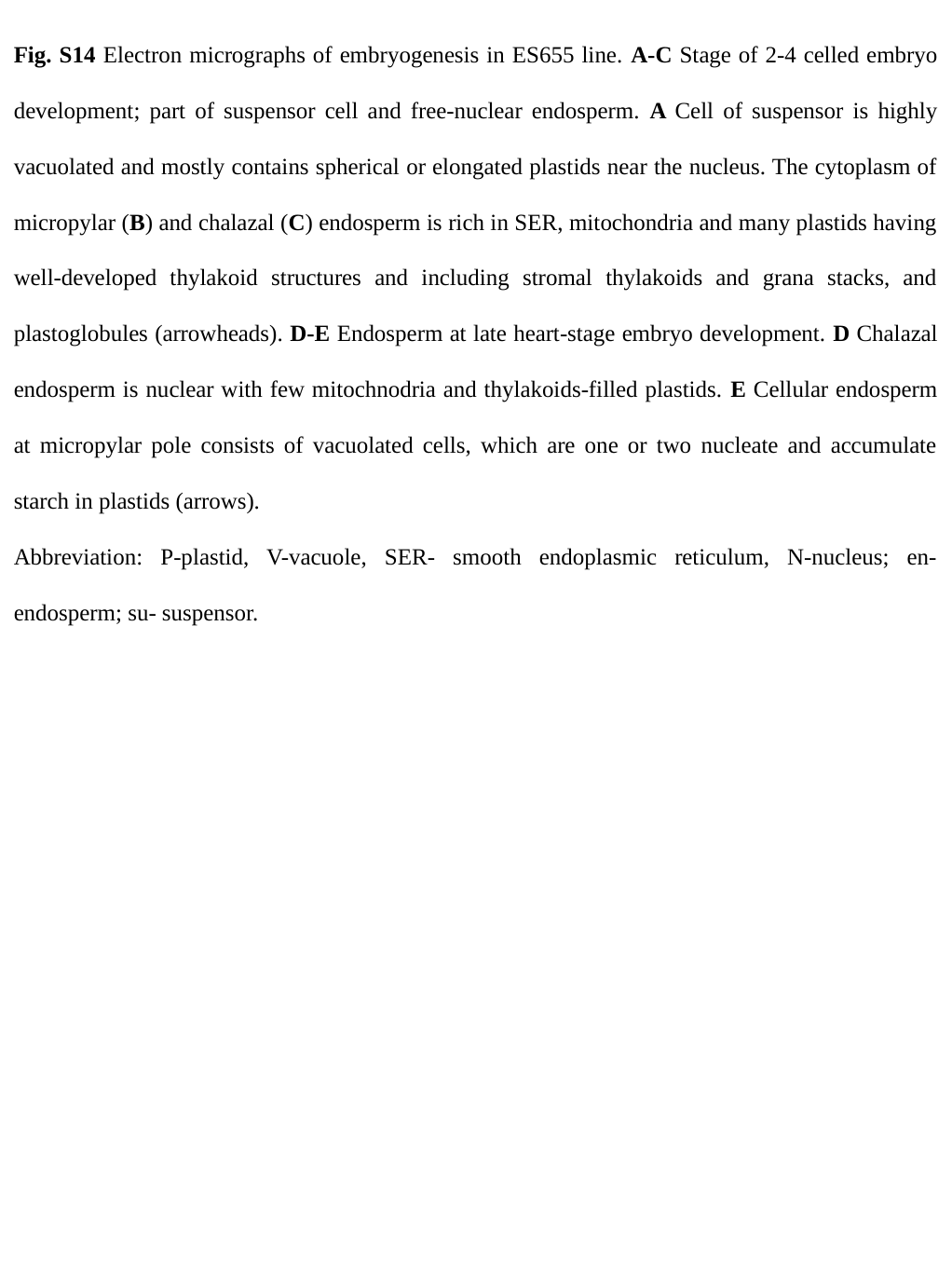

Fig. S14 Electron micrographs of embryogenesis in ES655 line. A-C Stage of 2-4 celled embryo development; part of suspensor cell and free-nuclear endosperm. A Cell of suspensor is highly vacuolated and mostly contains spherical or elongated plastids near the nucleus. The cytoplasm of micropylar (B) and chalazal (C) endosperm is rich in SER, mitochondria and many plastids having well-developed thylakoid structures and including stromal thylakoids and grana stacks, and plastoglobules (arrowheads). D-E Endosperm at late heart-stage embryo development. D Chalazal endosperm is nuclear with few mitochnodria and thylakoids-filled plastids. E Cellular endosperm at micropylar pole consists of vacuolated cells, which are one or two nucleate and accumulate starch in plastids (arrows).
Abbreviation: P-plastid, V-vacuole, SER- smooth endoplasmic reticulum, N-nucleus; en- endosperm; su- suspensor.

## Slide 27
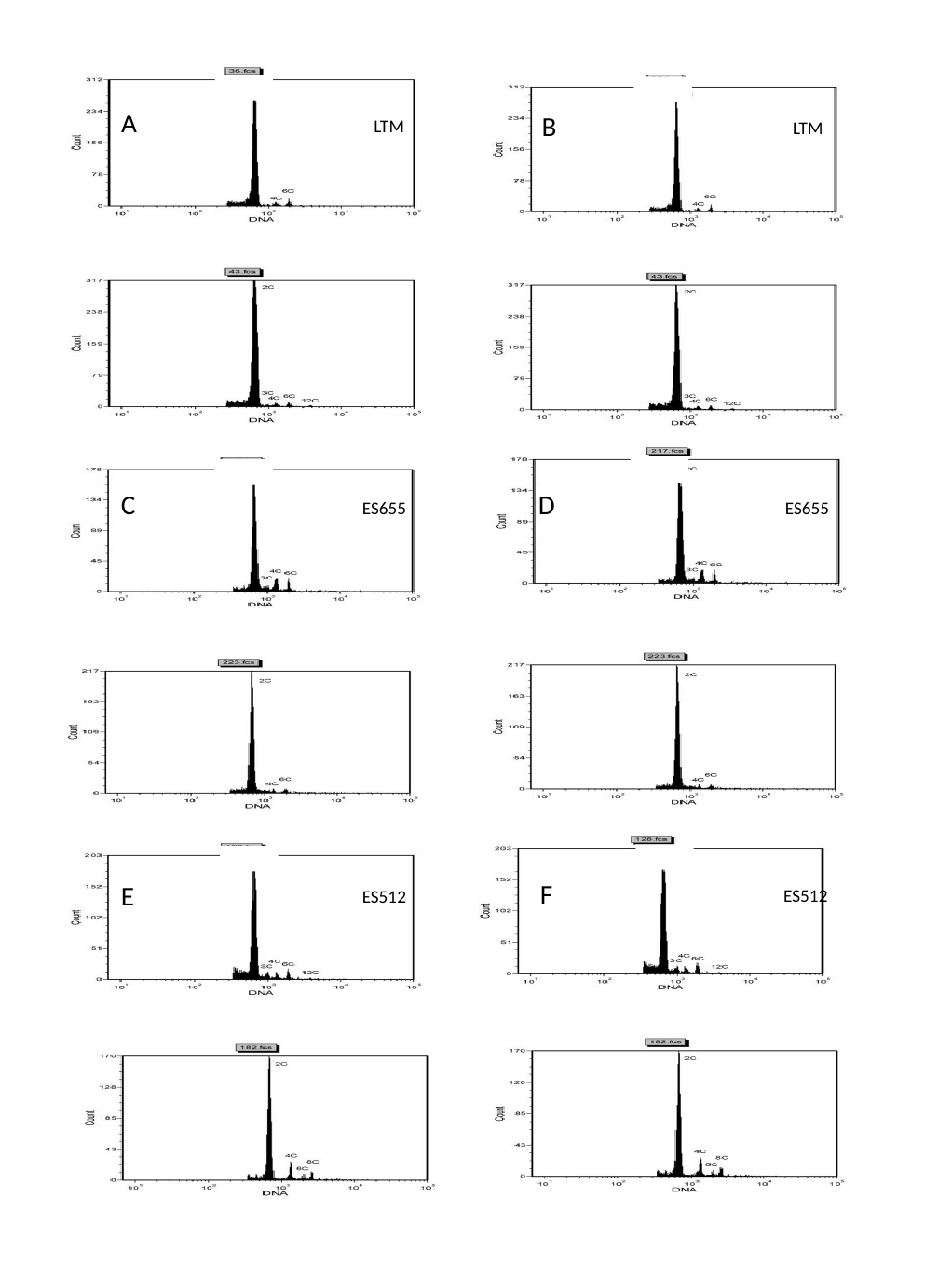

A
LTM
B
LTM
D
ES655
C
ES655
F
ES512
E
ES512

## Slide 28
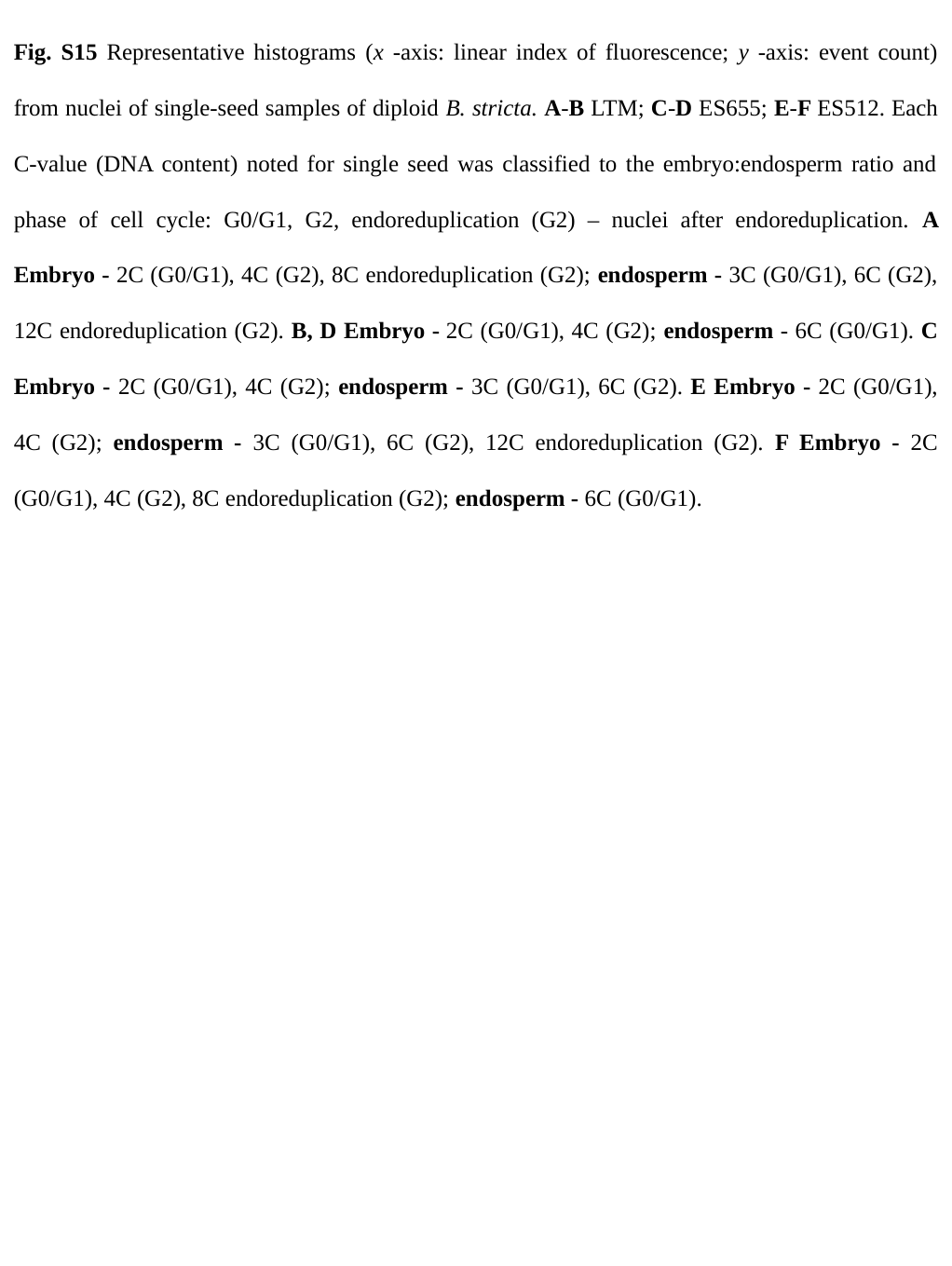

Fig. S15 Representative histograms (x -axis: linear index of fluorescence; y -axis: event count) from nuclei of single-seed samples of diploid B. stricta. A-B LTM; C-D ES655; E-F ES512. Each C-value (DNA content) noted for single seed was classified to the embryo:endosperm ratio and phase of cell cycle: G0/G1, G2, endoreduplication (G2) – nuclei after endoreduplication. A Embryo - 2C (G0/G1), 4C (G2), 8C endoreduplication (G2); endosperm - 3C (G0/G1), 6C (G2), 12C endoreduplication (G2). B, D Embryo - 2C (G0/G1), 4C (G2); endosperm - 6C (G0/G1). C Embryo - 2C (G0/G1), 4C (G2); endosperm - 3C (G0/G1), 6C (G2). E Embryo - 2C (G0/G1), 4C (G2); endosperm - 3C (G0/G1), 6C (G2), 12C endoreduplication (G2). F Embryo - 2C (G0/G1), 4C (G2), 8C endoreduplication (G2); endosperm - 6C (G0/G1).

## Slide 29
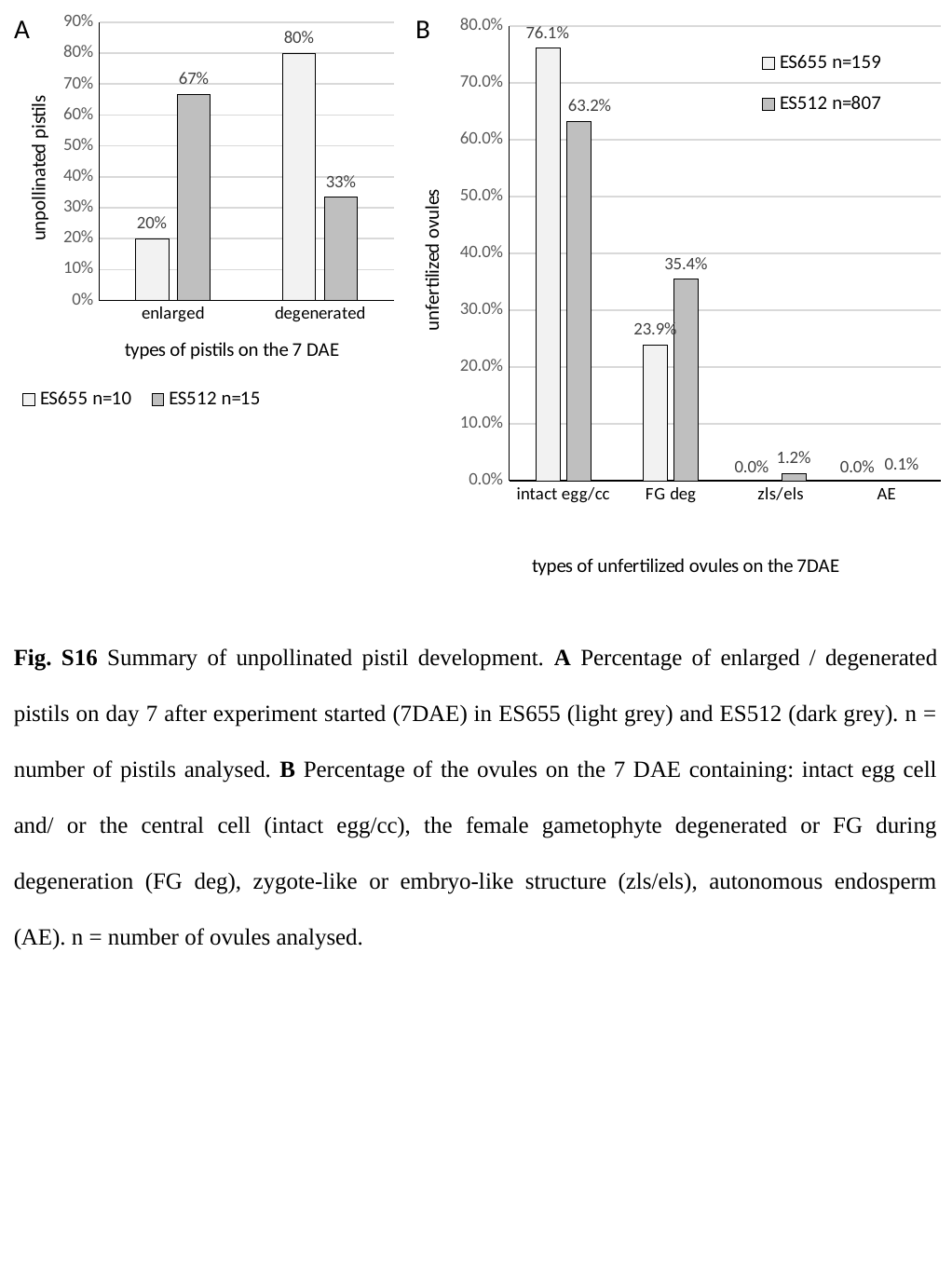

### Chart
| Category | ES655 n=10 | ES512 n=15 |
|---|---|---|
| enlarged | 0.2 | 0.6666666666666666 |
| degenerated | 0.8 | 0.3333333333333333 |
### Chart
| Category | ES655 n=159 | ES512 n=807 |
|---|---|---|
| intact egg/cc | 0.7610062893081762 | 0.6319702602230484 |
| FG deg | 0.2389937106918239 | 0.3543990086741016 |
| zls/els | 0.0 | 0.012391573729863693 |
| AE | 0.0 | 0.0012391573729863693 |A
B
Fig. S16 Summary of unpollinated pistil development. A Percentage of enlarged / degenerated pistils on day 7 after experiment started (7DAE) in ES655 (light grey) and ES512 (dark grey). n = number of pistils analysed. B Percentage of the ovules on the 7 DAE containing: intact egg cell and/ or the central cell (intact egg/cc), the female gametophyte degenerated or FG during degeneration (FG deg), zygote-like or embryo-like structure (zls/els), autonomous endosperm (AE). n = number of ovules analysed.
