# Supplementary material for: Establishing the cell biology of apomictic reproduction in diploid Boechera stricta (Brassicaceae)
Source: Ann Bot. 2018 Jul 6;122(4):513–39. doi: 10.1093/aob/mcy114 (PMC6153484; doi:10.1093/aob/mcy114)
Supplement: Supplementary Methods [file mcy114_suppl_supplementary_methods.docx]

**Materials and methods S1.** Tetrazolium chloride test (TTC) for seeds viability.

Four capsules were tested for seed viability by tetrazolium chloride method (TTC; Van Waes and Debergh, 1986; Rykaczewski *et al.*, UG, Gdańsk, Poland, unpubl. res., modified). After pre-treatment (1% of sodium hypochloride solution, pH 7.0, 15 minutes) seeds were incubated in 1% TTC in phosphate buffer saline at 37 °C for 24 hours. Seeds analyses were performed under a stereomicroscope (Nikon SMZ 1500).

**Van Waes JM, Debergh PC. 1986.** *In vitro* germination of some Western European orchids. *Physiolologia Plantarum* 67: 253–261.

**Materials and methods S2.** Flow cytometric seed screening (FCSS).

High-throughput flow cytometry seed screening was performed according to Aliyu *et al*., (2010; 46 seeds per genotype). For conventional method (Matzk *et al*., 2001), seeds were chopped with a sharp razor blade in a plastic Petri dish with 0.6 ml nucleus-isolation buffer (0.1 M Tris, 2.5 mM MgCl_2 ._6H_2_O, 85 mM NaCl, 0.1% Triton X-100; pH 7.0), supplemented with 4’,6-diamidino-2-phenylindole (DAPI; 2 µg/ml). After chopping, the suspension was passed through a 30 µm mesh nylon filter and incubated 10 min on ice before flow cytometric analysis. For each sample, 2000-4000 nuclei were analyzed, using a CyFlow Ploidy Analyzer flow cytometer (Sysmex Partec). A logarithmic scale was applied, and genotype LTM served as external standard. Analyses were performed on about 150 replicates (seeds) for each seed sample (genotype). Histograms were evaluated using CyFlow Cube 6 v. CFG computer program.
