# Supplementary material for: Establishing the cell biology of apomictic reproduction in diploid Boechera stricta (Brassicaceae)
Source: Ann Bot. 2018 Jul 6;122(4):513–39. doi: 10.1093/aob/mcy114 (PMC6153484; doi:10.1093/aob/mcy114)
Supplement: Supplementary Table S1 [file mcy114_suppl_supplementary_table_s1.docx]

Table S1. Summary of the main results from *B. stricta* study. n/a - not applicable

| genotype  type of analysis | LTM | ES655 | ES512 |
| --- | --- | --- | --- |
| **Phenotype identification** | | | |
| Leaf trichomes | sessile and 2-rayed | sessile and 2-rayed | sessile and 2-rayed |
| Flower morphology | *B. stricta* phenotype | *B. stricta* phenotype | *B. stricta* phenotype |
| Pollen morphology | ellipsoid with symmetric colpi | ellipsoid with symmetric colpi | ellipsoid with symmetric colpi |
| Number of seeds produced | 63-100 | 50-100 | from a few to 100 or more |
| Seedling germination | ±100% | ±100% | ±60% |
| Seed viability test | 100% viable | 80-90% viable | 80-90% viable, some seeds underdeveloped |
| **Genotype identification** | | | |
| ITS | n/a | L | L |
| cpDNA haplotype | AH^(a)^ | BF | DG |
| *APOLLO* alleles | n/a | Homozygous sex alleles | Homozygous sex alleles |
| Microsatellite loci | *B. stricta* “LTM” genotype | *B. stricta*;  *B. fendleri* x *B. spatifolia* x *B. stricta* | *B. stricta*;  *B. fendleri* x *B. stricta* |
| Chromosome counting | 2n=14 | 2n=14 | 2n=14 |
| Mode of reproduction | Sexual (2C:3C);  apomictic (2C:6C; 2C:4C) | Sexual (2C:3C);  apomictic (2C:6C) | Sexual (2C:3C);  apomictic (2C:6C; 2C:4C) |
| **Embryology assessment** | | | |
| Megasporogensis | MMCs, dyads, triads, tetrads, FM  (1-4) | MMCs, dyads, triads, tetrads, FM  (1-5) | MMCs, dyads, triads, tetrads, FM  (1-5) |
| Megagametogenesis | Undisturbed; Polygonum-type  (1-4) | Undisturbed; Polygonum-type  (1-6) | Undisturbed or disturbed; Polygonum-type  (1-6) |
| Microsporogensis | tetrads (a very few dyads, triads  (2-3) | tetrads (a very few triads)  (1-3) | tetrads (a very few triads)  (1-3) |
| Microgametogenesis | Undisturbed till maturity, bulgy 3-nucleate grains or sometimes collapsed ones  (2-3) | Undisturbed till maturity, bulgy 3-nucleate grains or collapsed ones  (1-3) | Undisturbed till maturity, bulgy 3-nucleate grains and collapsed ones; pollen sterility in a few individuals  (1-3) |
| Seed development | Undisturbed, Onagrad-type  (2) | Undisturbed, Onagrad-type  (1-2; 5-6) | Undisturbed, Onagrad-type; or disturbed, with embryo abortion  (1-2; 5-6) |
| Parthenogenesis occurrence | n/a | No autonomous development  (2) | Autonomous development of embryo and endosperm in a few ovules  (2) |

^(a)^ LTM has AH haplotype, according by Schranz *et al*. (2005, 2007); data not obtained in this study.

^(1-6)^ group of methods used for the type of analysis in this publication: 1- paraffin sections; 2- clearing technique, 3- callose detection; 4- Jim13 immunolabeling; 5- cytoskeleton structure; 6-transmission electron microscopy.

**Schranz ME, Dobeš C, Koch MA, Mitchell-Olds T**. **2005**. Sexual reproduction, hybridization, apomixis, and polyploidization in the genus *Boechera* (Brassicaceae). *American Journal of Botany* **92**: 1797–1810. doi: 10.3732/ajb.92.11.1797.

**Schranz ME, Windsor AJ, Song B-H, Lawton-Rauh A, Mitchell-Olds T**. **2007**. Comparative genetic mapping in *Boechera stricta*, a close relative of *Arabidopsis*. *Plant Physiology* **144**: 286–98. doi: 10.1104/pp.107.096685.
