# Supplementary material for: Establishing the cell biology of apomictic reproduction in diploid Boechera stricta (Brassicaceae)
Source: Ann Bot. 2018 Jul 6;122(4):513–39. doi: 10.1093/aob/mcy114 (PMC6153484; doi:10.1093/aob/mcy114)
Supplement: Supplementary Table S2 [file mcy114_suppl_supplementary_table_s2.docx]

**Table S2.** List of primer names, labels and sequences used in the SSR analysis

| **primer name** | **Label** | **Forward primer** | **Reverse primer** |
| --- | --- | --- | --- |
| **ICE3** | 6-FAM | GACTAATCATCACCGACTCAGCCAC | ATTCTTCTTCACTTTTCTTGATCCCG |
| **ICE14** | HEX | TCGAGGTGCTTTCTGAGGTT | TACCTCACCCTTTTGACCCA |
| **a1** | 6-FAM | GTCTATTCGAGGACGCC | AGGTTGGGTAGGTGAAG |
| **a3** | HEX | AGCTTTGTTTGCAATGGAG | GTGAGAATAATATTGACC |
| **b6** | HEX | GCAAAAGATCTTCATGGGAC | TGCCATTTCTTTCCCTAGTG |
| **c8** | 6-FAM | TTCCGGGTATCATTCCTAG | GTTGTAAGTTCTTTCTCAG |
| **e9** | HEX | GCGTATCTCGAATCACCTTTG | CTCCCCCTGAGTTTTTCAAG |
| **BF3** | 6-FAM | TTTTTAGACAGTAGTGGCTGTGAG | ACTTCGTTCCAGGCTCGTC |
| **BF9** | 6-FAM | AAACACATTCCCGTCAGCTC | TTGATTGAATCCTGCGTTTG |
| **BF11** | 6-FAM | TCCTCCATTGTAGAGCAGAGC | CCATTGCTTAAACCCTAAACC |
| **BF15** | 6-FAM | CAGCATCTCCTTTGGGTTTG | ACTTGCTCCTTTGCATGACC |
| **BF18** | HEX | AACCTCCCAAGATTCGCTTC | TTCGCCATTGTTGTGATTTG |
| **BF19** | HEX | ACCGCATTGGTGTTGTGTC | ATAACGGACGCGACCAAAG |
| **BF20** | HEX | TTCTCGGGAAAGTAATGAGGAG | GCAAATCTGACCAATGCAAG |
| **Bdru266** | HEX | TTTAATTTGTGCGTTTGATCC | CAAAATCGCAGAATGAGAGG |
| **H34/ICE4*** | 6-FAM | CACGAGGAATCTGGCATGGTCG | AGCGATTGCAAGCGGCTCAAG |
| **d3*** | 6-FAM | GGTTATGTGAGAGTTAAG | ATTGTTGAATGCAACAGG |
| **H105/SLL2*** | 6-FAM | CATGTACTGGGATTCAGTGTCC | CGTCCTTTGTGTGGTTACACG |

Primers data according by Li *et al*., 2017 and (*) Claus *et al*., 2002; Dobeš *et al*., 2004
