# Supplementary material for: Establishing the cell biology of apomictic reproduction in diploid Boechera stricta (Brassicaceae)
Source: Ann Bot. 2018 Jul 6;122(4):513–39. doi: 10.1093/aob/mcy114 (PMC6153484; doi:10.1093/aob/mcy114)
Supplement: Supplementary Table S3 [file mcy114_suppl_supplementary_table_s3.docx]

**Table S3.** Number of alleles observed at eighteen microsatellite loci in analysed individuals ES655 and ES512 accessions, and  *B. stricta*  “LTM” as a comparative line.

| **id**  **locus** | **LTM** | **ES655** | **ES512** |
| --- | --- | --- | --- |
| **ICE3** | 75 | 83 | 77 |
| **ICE14** | 223 | 223 | 223 |
| **a1** | 238 | 238 | 238 |
| **a3*** | 249 | 249; 255 | 249 |
| **b6** | 302 | 302 | 302 |
| **c8** | 238 | 262 | 262 |
| **e9** | 204 | 204 | 204 |
| **BF3** | 99 | 101 | 109 |
| **BF9** | 104 | 82 | 90 |
| **BF11** | 96 | 88 | 88 |
| **BF15** | 99 | 99 | 99 |
| **BF18** | 117 | 117 | 118 |
| **BF19** | 149 | 143 | 143 |
| **BF20** | 221 | 213 | 213 |
| **Bdru266** | 119 | 121 | 129 |
| **H34/ICE4** | 183 | 183 | 183 |
| **d3** | 166 | 163 | 166 |
| **H105/SLL2** | 314 | 314 | 314 |

***** **a3** locus can occasionally exhibit more than the expected number of alleles in plants of known ploidy and was therefore excluded from heterozygosity calculations (according by Li *et al*., 2017)
