# Supplementary material for: Establishing the cell biology of apomictic reproduction in diploid Boechera stricta (Brassicaceae)
Source: Ann Bot. 2018 Jul 6;122(4):513–39. doi: 10.1093/aob/mcy114 (PMC6153484; doi:10.1093/aob/mcy114)
Supplement: Supplementary Table S4 [file mcy114_suppl_supplementary_table_s4.docx]

|  |  | MMC vs ovule development | | | | | | Dyad vs ovule development | | | | | | | Triad/tetrad vs ovule development | | | | | | |
| --- | --- | --- | --- | --- | --- | --- | --- | --- | --- | --- | --- | --- | --- | --- | --- | --- | --- | --- | --- | --- | --- |
| Genotype | No. ovules at MMC | early | **%** | SE | late | **%** | SE | No. ovules at dyad | early | **%** | SE | late | **%** | SE | No. ovules at triad/tetrad | early | **%** | SE | late | **%** | SE |
| **LTM** | 106 | 106 | **100** | - | 0 | **0** | - | 27 | 27 | **100** | - | 0 | **0** | - | 365 | 311 | **85** | 0.02 | 54 | **15** | 0.05 |
| **655** | 40 | 40 | **100** | - | 0 | **0** | - | 116 | 109 | **94** | 0.01 | 7 | **6** | 0.03 | 244 | 199 | **82** | 0.01 | 45 | **18** | 0,02 |
| **512** | 40 | 34 | **85** | 0.05 | 6 | **15** | 0.11 | 160 | 132 | **83** | 0.02 | 28 | **18** | 0.05 | 271 | 166 | **61** | 0.02 | 105 | **39** | 0.02 |

Table S4. Meiotic stages occurrence (early or late) in relation to ovule (integument) development. The frequency was evaluated from the ovules used for callose detection. SE= standard error; at

*P* < 0.05 level.
